# Rubisco functional integrity requires a dedicated three-step maturation pathway

**DOI:** 10.1126/sciadv.aeh5060

**Published:** 2026-09-09

**Authors:** Dong Xie, Jens S. Mühlenbeck, Patrick Schall, Jean-Baptiste Boyer, Cyril Dian, Laila Sago, Yuwei Wang, Lucile Jomat, Juergen Eirich, Frédéric Frottin, Iris Finkemeier, Bernhard Grimm, Carmela Giglione, Thierry Meinnel

**Affiliations:** ^1^ Université Paris-Saclay, CEA, CNRS, Institute for Integrative Biology of the Cell (I2BC), 91198 Gif-sur-Yvette, France.; ^2^ Plant Physiology, Institute of Plant Biology and Biotechnology, University of Muenster, Schlossplatz 7, D-48149 Muenster, Germany.; ^3^ Humboldt-Universität zu Berlin, Institute of Biology/Plant Physiology, Philippstraße 13 (Building 12), 10115 Berlin, Germany.

## Abstract

Proteins generally begin with methionine, which is often removed to expose the second residue. RuBP carboxylase/oxygenase (Rubisco), the most abundant enzyme on Earth and a major carbon and nitrogen reservoir, escapes this paradigm. Its catalytic subunit, RbcL, undergoes unusual amino terminal maturation, including atypical initiator methionine-serine cleavage and proline acetylation, a modification not seen in other biological systems. We define a specialized pathway of two sequential aminopeptidases and an amino terminal acetyltransferase, dedicated to RbcL, that executes processing. This maturation is coupled to chaperone-assisted holoenzyme assembly, enhances activation by Rubisco activase, and shields the complex from degradation during senescence. Disruption of this pathway reduces Rubisco accumulation and catalytic competence, revealing an evolutionarily tailored mechanism integrating protein maturation, assembly, and stability of a central metabolic enzyme.

## INTRODUCTION

Photosynthetic organisms convert atmospheric CO_2_ into biomass, sustaining nearly all life on Earth. At the heart of this indispensable reaction is d-ribulose-1,5-bisphosphate carboxylase/oxygenase, Rubisco, the primary CO_2_-fixing enzyme of the Calvin-Benson cycle (*1*). Rubisco is also the most abundant protein on the planet and a major reservoir of nitrogen and carbon in leaves (*2*–*4*). Rubisco accounts for up to ∼70% of soluble leaf protein in crops, and its controlled mobilization during stress and senescence fuels nutrient recycling, plant development, and productivity (*5*–*8*). Therefore, understanding the factors that influence Rubisco’s catalytic efficiency, biogenesis, metabolic repair, and accumulation represents a critical challenge for humanity, particularly in the face of climate change, global warming, and the urgent need to feed a growing global population.

Plant and algal Rubisco is a hexadecamer (L8S8, ∼530 kDa) composed of eight large subunits (RbcL, ∼53 kDa) and eight small subunits (RbcS, ∼15 kDa). RbcS subunits are usually translated from several nuclear genes and imported into chloroplasts, where they are finally assembled with RbcL after cleavage of the chloroplast transit peptide (cTP). RbcL, the large subunit responsible for catalysis, is encoded by a single gene in the chloroplast genome. The functional pool of Rubisco holoenzyme is regulated by proteostatic control, including (i) cotranslational processing of the nascent RbcL; (ii) chaperonin-and assembly factor–assisted folding and formation of the active L8S8 complex; (iii) remodeling by Rubisco activase (Rca), the adenosine triphosphatase (ATPase) that releases inhibitory sugar phosphates from the active site and maintains Rubisco in a catalytically competent state; and (iv) monitoring and degradation to recycle damaged or unassembled Rubisco subunits (*9*–*13*). Despite decades of study, central features of Rubisco proteostasis—the integrated control of its maturation, activity, and selective degradation—remain unresolved.

A long-standing peculiarity of Rubisco is concealed at the highly conserved N terminus of RbcL. In plants and more broadly across green lineages, although the gene encodes an initial Met^1^-Ser^2^-Pro^3^ sequence (methionine-1, M1; serine-2, S2; proline-3, P3), the mature protein consistently carries an unusual N-terminally acetylated Pro, *ac-*Pro^3^ (*14*). This modification is notable because, while N-terminal acetylation is very common in the eukaryotic cytosol and contributes significantly to proteostasis (*15*–*17*), Pro acetylation at a protein N terminus has been documented only for RbcL to date. To better appreciate the uniqueness of the RbcL N terminus, one has to consider the general rules of plastid N-terminal processing. Protein synthesis in plastids, as in bacteria, begins with an N-formylated methionine (*fo*-Met^1^), which is typically removed by peptide deformylases (PDFs). Methionine aminopeptidases 1 (MetAP1s) in plastids may then excise the initiator methionine to reveal residue 2 (*18*–*21*). These canonical steps account for most N-termini encoded in the organelle, but they alone cannot generate a stable RbcL, which begins at Pro^3^, nor are they responsible for the acetylation of the neo N-terminal Pro. Accordingly, the uniqueness of RbcL processing reflects the absence—across all known organisms—of a defined enzymatic system capable of unmasking Pro^3^ and subsequently acetylating its α-amino group. Plastid N-terminal acetylation is catalyzed by a diversified set of *N*-acetyltransferases (NATs) known as NatGs or GNATs (*15*, *22*–*25*). Comparative genomics and proteomics have identified at least eight GNATs spanning two phylogenetic clades, NAA90 and NAA70 (*22*, *23*). GNATs target both plastid-encoded and plastid-imported nuclear-encoded proteins, including subunits of the core photosynthetic complexes (*22*, *23*, *26*). Despite this repertoire, substrate rules remain poorly defined, and none of the yet characterized GNATs have been shown to acetylate an N-terminal Pro of a plastid-localized protein. With its conserved N-terminal *ac-*Pro^3^, RbcL maturation thus defies established processing rules, suggesting a dedicated and previously uncharacterized pathway. Elucidating how this modification arises, and why it is maintained across green lineages, is essential for understanding Rubisco biogenesis, stability, and regulation.

In this study, we unravel the unique and specific pathway leading to RbcL N-terminal processing and show that it is crucial for most of the features leading to Rubisco as a highly abundant and reactive protein catalyst in plant leaves. We propose that these modifications strengthen Rca interaction, and that interfering with this pathway reduces Rubisco accumulation and promotes early senescence.

## RESULTS

### RbcL predominantly, but not exclusively, accumulates with an *ac*-Pro^3^ N terminus

**Fig. 1.**
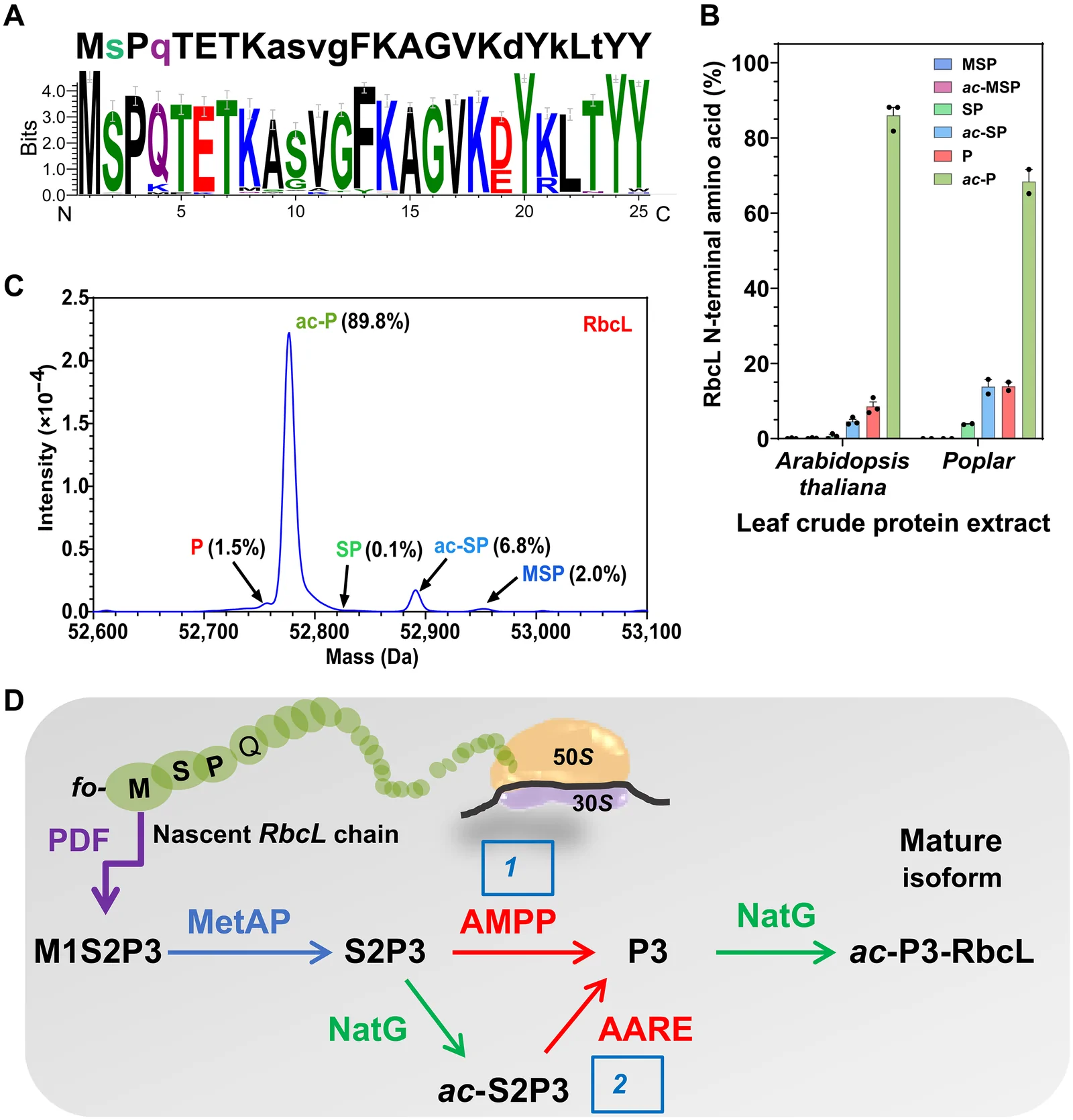
Progressing toward the actual pathway of RbcL N-terminal processing in the plastids of green plants. (**A**) Top: Conserved residues of the alignment (*N* = 30) displayed in fig. S1A are shown in uppercase letters, while highly conserved residues are displayed in lowercase. Residues that differ from the consensus sequence are highlighted by color. Bottom: WebLogo (https://weblogo.threeplusone.com/) generated from an alignment of RbcL random sequences (*N* = 200) illustrates the frequency of occurrence for each residue. (**B**) Relative abundance of RbcL N-termini in *A. thaliana Col-0* lines (left, *N* = 3) and in poplar (right, *N* = 2). A crude protein extract was analyzed by SDS-PAGE. The 55-kDa region band was cut and hydrolyzed with chymotrypsin. Each band (200 ng) was analyzed on a Bruker timsTOF Pro 2. Raw data were processed with FragPipe, and the MaxLFQ intensity of each N-terminal isoform was used to quantify their relative abundance. For each sample, ion intensities of N-terminal peptides of RbcL (positions 1 to 3) were summed, and the relative distribution of each ion is reported. (**C**) Intact mass analysis after deconvolution of purified Rubisco from WT *A. thaliana* line. The spectrum is a zoom of the full mass spectrum centered on RbcL. The various isoforms are indicated with arrows. (**D**) Hypothetical models best explaining RbcL processing. The two alternative pathways (model 1: “1”; model 2: “2”) start with *fo*-MSP, the N terminus resulting from ribosomal RbcL mRNA translation (fig. S1B). The two pathways recapitulate the putative catalysts potentially involved. Both pathways converge on a common same final step catalyzed by a NatG (also known as GNAT). The two NatGs reported here may or may not correspond to the same enzyme form. *fo* is N-formyl, *ac* is N-acetyl. PDF, peptide deformylase; MetAP, methionine aminopeptidase; AMPP, aminopeptidase; NatG, N-terminal acetyltransferase G; AARE, acylamino acid releasing enzyme.

Relative to the initiator Met (AUG^1^), which is the bona fide start codon, the third codon of plant RbcL encodes Pro. An in-frame AUG^2^ alternative start seven codons downstream is often present but lacks Shine-Dalgarno features and is not conserved (fig. S1). During translation, RbcL thus always starts as *N*-formyl-Met^1^-Ser^2^-Pro^3^, *fo*-M1S2P3 (Fig. 1A and fig. S1B). To characterize and quantify the mature N-termini of RbcL in *Arabidopsis thaliana* leaves of 3-week-old (W3) plants, we first used a combination of bottom-up mass spectrometry (MS) investigations. Together, the data revealed that the N terminus of RbcL consisted of 86.0% *ac-*Pro^3^, 8.6% Pro^3^, 4.6% *ac*-Ser^2^, and 0.6% Ser^2^ (Fig. 1B, left). A similar result was obtained with another land plant, poplar (Fig. 1B, right). Moreover, the intact mass analysis of purified Rubisco from *A. thaliana* Col-0 [wild type (WT)] leaves resolved 89.8% *ac-*Pro^3^, 1.5% Pro^3^, 6.8% *ac*-Ser^2^, 0.1% Ser^2^, and 2.0% Met^1^ (Fig. 1C). These two datasets are consistent, with the Met^1^ start species identified as an additional, albeit minor, accumulating isoform of RbcL. Accordingly, the peptide Atlas database (https://peptideatlas.org/builds/arabidopsis/) reports the identification of four RbcL proteoforms—*ac-*Ser^2^, Ser^2^, *ac-*Pro^3^, and Pro^3^—with *ac-*Pro typically prevalent www.psb.ugent.be/webtools/ptm-viewer/protein.php?id=ATCG00490.1). Overall, the data indicated that the acetylation yield of both Ser^2^ and Pro^3^ was almost complete (∼95%) and that Pro^3^ was the predominant N terminus (>90%).

The detection of *ac*-Ser^2^ and Ser^2^ together with Met^1^, all at low abundance at the N terminus of RbcL, supports the sequence in which canonical deformylation and Met^1^ excision occur before exposure of Pro^3^ and subsequent acetylation to *ac*-Pro^3^. This observation suggests that models 1 and 2, as shown in Fig. 1D, could account for RbcL processing. Met^1^ cleavage by plastid MetAPs followed by acetylation by an as-yet unidentified plastid GNAT might generate *ac*-Ser^2^. *ac*-Ser^2^ could then be converted by an Acylamino-acid-releasing enzyme (AARE), which would release *ac*-Ser^2^ to promote the formation of Pro^3^ (model 2). Alternatively, if acting before any acetylation may occur, then an aminopeptidase (AMPP; model 1) would release the neo-amino acid—Ser^2^ in all land plants—to unmask the N-terminal Pro before N-terminal acetylation (Fig. 1D). This pathway would involve acetylation of both Ser^2^ and Pro^3^ species with the Ser^2^ pathway acting as a minor route. Although both models 1 and 2 are the most likely, as they closely fit the data, it cannot be formally excluded that they act simultaneously, possibly compensating for each other. The identification of the Met^1^ species strongly suggested however that PDF does act on RbcL maturation. These data are in agreement with previous investigation showing that *PDF1B* knockout (KO) *A. thaliana* lines or *Chlamydomonas reinhardtii* cells treated with actinonin, a potent PDF inhibitor (*27*), both exhibit RbcL isoforms with a pronounced migration delay of RbcL on SDS–polyacrylamide gel electrophoresis (SDS-PAGE), an effect attributed to the retention of an additional N-terminal *fo*-Met^1^-Ser^2^ dipeptide (*21*).

### AtMetAP1C drives initiator methionine processing of RbcL

**Fig. 2.**
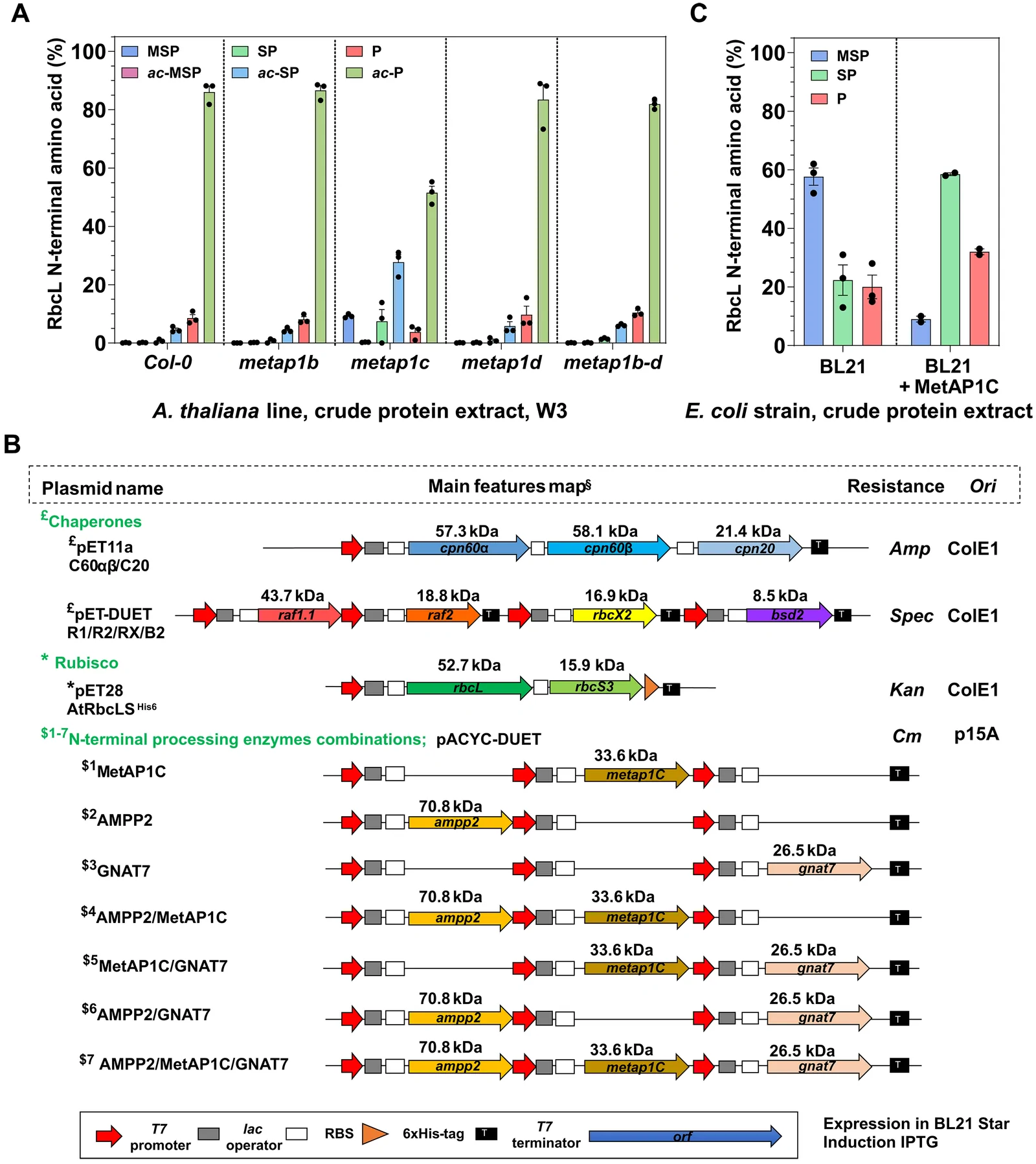
Plastid MetAP1C displays Met excision activity on RbcL processing both in vivo and in vitro*.* (**A**) Relative abundance of RbcL N-termini in *Arabidopsis thaliana* plastid MetAP mutant lines (*N* = 3). The sample preparation method is same as that described in Fig. 1B. (**B**) Plasmids used to express and assemble function Rubisco in *E. coli*. * means molecular chaperones that ensure proper assembly of *At*Rubisco (*28*). £, His-tagged *At*Rubisco expression plasmid (*29*). $1-7, this work, N-terminal processing enzymes combinations, the plasmid series mainly contains one, two, or three of these ORFs, in this order. § denotes all ORFs are devoid of their plastid N-terminal targeting signal. Only RbcL sequence is WT—unless otherwise stated—as this one gene is plastid-encoded. (**C**) Plant MetAP1C display N-terminal RbcL Met cleavage activity in *E. coli*. The Rubisco’s complementation was conducted by AtMetAP1C expressed from pACYC-Duet-MetAP1C in the ERBP (Fig. 2B, $1). Sample analysis was as in (A). Only the RbcL N-terminal peptides are displayed (*N* = 3).

Three MetAPs are involved in plastid Met^1^ cleavage in *A. thaliana* (*19*), i.e., MetAP1B (At3g25740), MetAP1C (At1g13270), and MetAP1D (At4g37040) (see Materials and Methods for updated nomenclature of plant MetAPs). We examined RbcL N-terminal processing in KO *Arabidopsis* lines of each of the three plastid MetAPs. The N-terminal status of RbcL in both *metap1b* and *metap1d* lines was indistinguishable from WT ( Fig. 2A). By contrast, the N terminus of RbcL was markedly altered in the *metap1c* background, with total accumulation of the *ac*-Ser^2^ and Met^1^ species reaching levels comparable to those of the *ac*-Pro^3^ isoform (Fig. 2A). These data indicate that among the three plastid MetAPs, MetAP1C is the main enzyme that contributes significantly to RbcL Met excision in vivo, since MetAP1B and MetAP1D only partially compensate for RbcL N-terminal processing. Consistently, a *metap1b-metap1d* double KO mutant line shows normal RbcL processing, demonstrating that MetAP1C alone is sufficient for complete Met^1^ removal (Fig. 2A). Notably, MetAP1C is the only MetAP that is exclusively localized to the plastid, whereas MetAP1B and MetAP1D are dual-targeted to both plastids and mitochondria (*19*). Thus, MetAP1C is critical for the maturation of RbcL and promotes its initiator methionine (Met^1^) removal.

To further assess whether MetAP—or any other catalyst—can process Rubisco, we engineered a compatible vector (Fig. 2B) to express up to three different open reading frames (ORFs) along with both Rubisco subunits in *Escherichia coli* BL21 cell derivatives, which provide the full chaperone machinery for proper folding of functional complexes (*28*, *29*). This in vivo reconstitution of the Rubisco biogenesis pathway in *E. coli* is hereafter referred to as the *E. coli*–based Rubisco biogenesis platform (ERBP). In the absence of any additional ORF, crude protein extracts from the ERBP exhibited both the Pro^3^ and Ser^2^-starting isoforms of RbcL at a minor level (∼20%), while the Met^1^ isoform predominated, accounting for ∼55% of all N-termini (Fig. 2C, left). Upon the expression of MetAP1C in the ERBP, the Met^1^ isoform was almost completely converted into the Ser^2^ form (Fig. 2C). These observations confirm MetAP1C can also mediate the removal of the initiator methionine from RbcL in the ERBP and establish this system as a robust platform to study the N-terminal processing properties of RbcL in the context of any ORF.

### Proline-specific aminopeptidase AMPP2 is the plastid homolog of *E. coli* PepP and catalyzes RbcL Ser^2^ removal

**Fig. 3.**
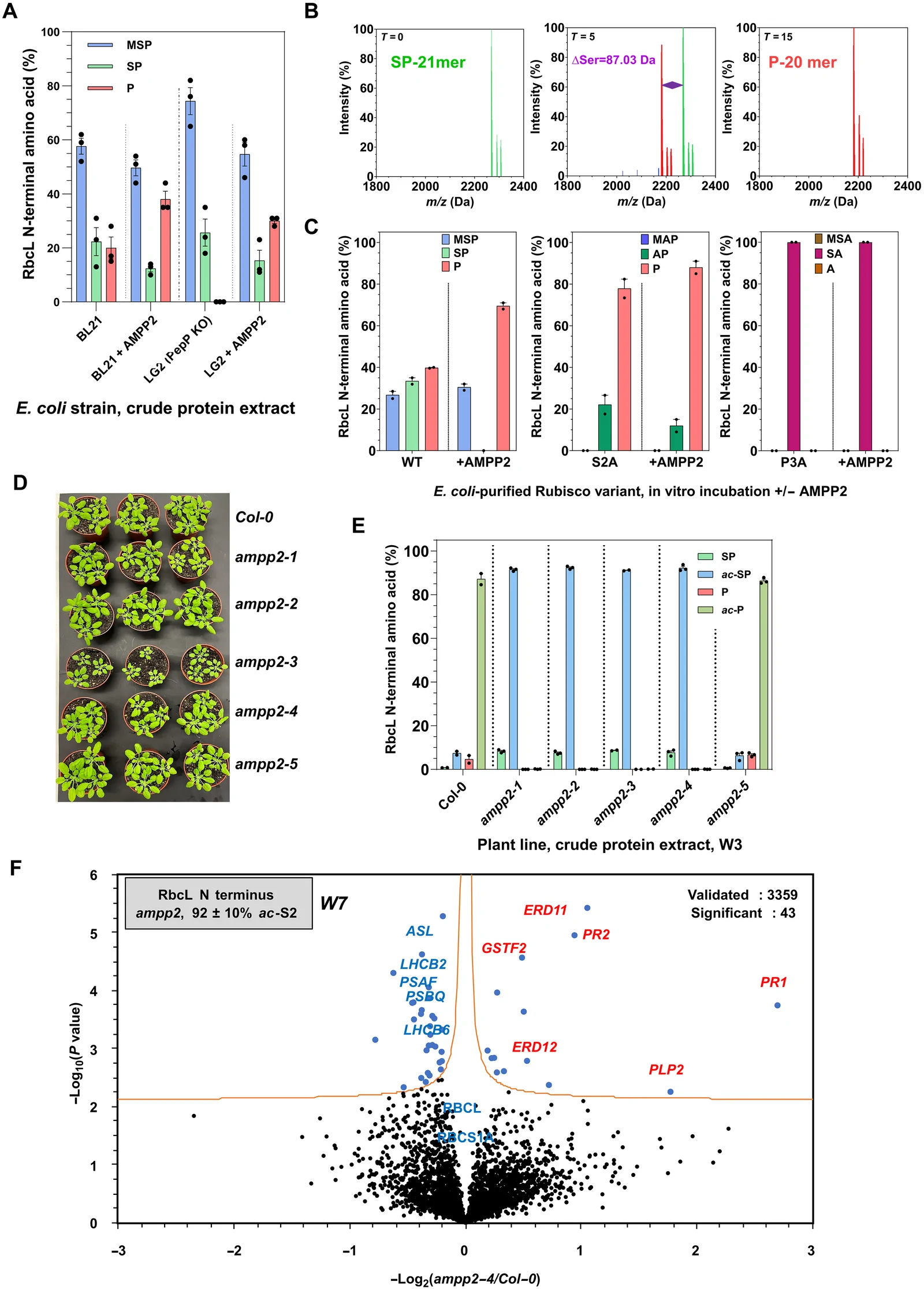
AMPP2 displays similar aminopeptidase activity as *E. coli* pepP and acts on RbcL processing and its functional impact in *A. thaliana.* (**A**) EcPepP and AtAMPP2 display similar RbcL cleavage activity in *E. coli*. Both Rubisco subunits were overexpressed from pBAD33-RbcLS in LG-2 and BL21 in the absence of chaperones. The *pepP* gene is deleted in strain LG-2 (*pepP*) and is functional in BL21. Each strain was complemented or not by AMPP2 expressed from pTrc99A-AMPP2 (fig. S4A). The sample was analyzed as in Fig. 2C (*N* = 3). (**B**) Time-course survey of the cleavage by AMPP2 of a 21-long peptide derived from RbcL as assessed by MALDI-TOF MS. The incubation time in minutes with AMPP2 is indicated on the left top of each spectrum. (**C**) In vitro cleavage of RbcL by AMPP2 and RbcL-Ala^2^ (S2A) and P3A Rubisco variants from *E. coli*. Ten micromolar of Rubisco was incubated with or without 0.5 μM AMPP2 for 15 min and analyzed by intact MS. The relative abundance of each species was then calculated as its proportion of the total signal (100%) and displayed (*N* = 2). (**D**) Phenotypes of five independent *ampp2* insertion lines compared to WT at W3. Line *ampp2-3* is smaller as it displays a 2-day longer germination time than the other lines for yet unknown reasons. Lines *ampp2-4* are KO lines, while *ampp2-5* is WT, as shown in fig. S5C. (**E**) Five independent lines with insertions in the AMPP2 gene were analyzed by N-terminomics. Quantification was as in Fig. 1B (*N* = 3). (**F**) Volcano plot of line *ampp2-4* grown at W7 (*N* = 4) compared to WT. Proteins with significant increased accumulation in the mutant line are indicated with blue dots, and relevant entry names are displayed in blue (down) or red (up). A list of significant changes is provided in data S1.

At this stage, we showed that an AARE was not involved in the pathway, thereby excluding model 2 (Fig. 1E, fig. S2, and Supplementary Text). We therefore challenged model 1 and searched an aminopeptidase (AMPP) specific for the N terminus that could account for residue 2 processing. This residue is not only Ser in land plant RbcL but also Ala or Val in chlorophytes (*18*). We failed to identify such an aminopeptidase, as all known plastid AMPP with specificity for the N-terminal residue correspond to large residues [Tyr/Asp/Glu/Leu/Met/Arg; see table 1 in (*18*)]. We then focused our attention on the penultimate residue resulting from MetAP cleavage, the strictly conserved Pro^3^. It had previously been observed that, when human cationic trypsinogen starting with Met^1^-Ala_2_-Pro^3^ was expressed in *E. coli*, both Met^1^ (70%) and Pro^3^ (30%) species accumulated (*30*). This description suggested that an AMPP was able to remove the N-terminal Ala_2_ in *E. coli* and generate an N-terminal Pro^3^ residue. The corresponding *E. coli* AMPP gene was identified as *pepP* (EC:3.4.11.9; UniProt ID P15034; Xaa-Pro aminopeptidase/AMPP) (*31*). *pepP* inactivation in the *E. coli* LG-2 strain background inhibited the cleavage, leading to N-terminal Pro^3^ from trypsinogen (*30*). We hypothesized that *E. coli* AMPP activity, encoded by *pepP*, also processes the Ser^2^ residue of RbcL, thereby promoting the occurrence of Pro^3^ as N-terminal residue. Analogous to trypsinogen, the expression of Rubisco in a PepP^+^ strain (BL21) led to marked accumulation of the Pro^3^-starting isoform (Fig. 3A, left). In contrast, similar experiments carried out with the *pepP*-deficient strain LG-2 led to accumulation only of the Met^1^ and the Ser^2^ isoforms (Fig. 3A, right). The Pro^3^ form could not be identified in this background, indicating that PepP processed the Ser^2^ of RbcL into Pro^3^ in *E. coli*.

We searched the *A. thaliana* proteome for PepP homologs and identified At3g05350 (AMPP2; Q8RY11) as a likely candidate (fig.S3A and Supplementary Text). The gene encoding the mature AMPP2 protein (73 kDa) was cloned into a bacterial expression vector compatible with expression of both Rubisco subunits in LG-2 and BL21 strains (fig. S4A), and its ability to restore RbcL processing to Pro^3^ was assessed by complementation. Upon AMPP2 expression in LG-2 (fig. S4B), the Pro^3^ form of RbcL accumulated to the expense of the Met^1^ and Ser^2^ forms, mimicking the situation of the PepP-expressing strain (Fig. 3A, right). The data show the ability of AMPP2 to remove the N-terminal Ser^2^ from RbcL and to act as a plant homolog of AMPP2 in *E. coli*.

To assess and characterize the associated enzyme activity, purified AMPP2 (fig. S4C) was incubated with peptides derived from the RbcL N terminus (i.e., Ser^2^ starting), which may serve as substrates in the model 1 processing pathway. The time course analysis of reaction products by matrix-assisted laser desorption/ionization (MALDI) MS, both in MS and MS/MS mode, revealed a progressive removal of the N-terminal Ser (Fig. 3B and fig. S4D). Initial rates of catalysis could be determined (Table 1 and fig. S4E). With a catalytic rate greater than 5 s^−1^, AMPP2 proved to be highly active using peptides with a length of only 14 residues, derived from the N terminus of premature RbcL and beginning with Ser_1_-Pro_2_ (Table 1), thereby further supporting the penultimate step of model 1. Using the 21–amino acid–long substrate, we observed that the AMPP2 activity was inhibited when Pro_2_ was substituted by Ala but stimulated upon N-terminal Ser substitution to Met (Table 1). This indicates that AMPP2 shows strong specificity toward Xxx-Pro-starting polypeptides. Therefore, AMPP2 also exhibits MetAP activity with strict specificity for Pro^2^, in contrast to canonical MetAPs that accept a broader spectrum of small residues, including Pro^2^. The Met cleavage activity of AMPP2 was even more efficient with a catalytic rate exceeding 37 s^−1^. This is consistent with the known properties of other AMPP family members, which preferentially cleave substrates with large N-terminal residues, such as Arg or Met. However, as Met is first removed by MetAP on RbcL, a small residue needs to be located at position 2 for AMPP2 to act. This likely imposes a restraint and a compromise and suggests adaptation of a preexisting machinery to RbcL processing. We lastly checked that AMPP2 has no AARE activity and displays a strong preference for RbcL sequence as verified when residues beyond Pro^2^ were substituted (Table 1). AMPP2 behaves therefore as a typical AMPP, exhibiting a strong preference for the N-terminal sequence of RbcL. Thus, AMPP2 aminopeptidase activity is essential for the Ser^2^ processing of N-terminal RbcL.

**Table 1.** Cleavage efficiency of AMPP2 is highly dependent on Pro^2^ and the downstream sequence. Peptides (0.5 mM) were incubated with 0.5-10 μM AMPP2 for 5 to 120 min. Residues 2 to 18 of the plastid-encoded PsbL protein (UniProt: P60129; gene: AtCg00560) were used for the replacement. A C-terminal Ala-Ala dipeptide was added to improve the solubility of the resulting peptide.

Sequence	Peptide length	Variation/changes	Relative rate (%)*
SPQTETKASVGFKAGVKEYKL	21	–	100
*ac*-SPQTETKASVGFKAGVKEYKL	21	*N*-acetyl	0
SPQTETKASVGFK	13	Shortened to 13 residues	29
SPQTETKASVGFKAGVK	17	Shortened to 17 residues	18
SAQTETKASVGFKAGVKEYKL	21	P2A	0.01
MPQTETKASVGFKAGVKEYKL	21	S1M	652
SPTQSNPNEQSVELNRTSLAA	21	Amino acids 3–21 replaced	0.3
SPQTETKAS	9	Shortened to 9 residues	†

* A value of 100 was given to the WT 21 mer (5.78 s^−1^).

† Low signal-to-noise ratio associated with this peptide.

### AMPP2 specifically converts RbcL’s Ser^2^ to Pro^3^ in a bacterial expression system

Rubisco purified from the ERBP consisted of a mixture of isoforms present in similar proportions and starting at Met^1^, Ser^2^ and Pro^3^ [Fig. 3C (left) and fig. S3B]. We observed that the proportion of the Met^1^ isoform of RbcL was two- to threefold lower in assembled Rubisco complexes purified from the ERBP than in the crude total extract, which predominantly contained unassembled subunits (Fig. 2C, left).

The incubation of purified Rubisco from the ERBP with AMPP2 in vitro led to the disappearance of the Ser^2^ isoform in favor of the Pro^3^ form, while the Met^1^ form remained unchanged (Fig. 3C, left). Upon the coexpression of AMPP2 in the ERBP, the Ser^2^ isoform was converted into the Pro^3^ form. However, this conversion was incomplete, as the Ser^2^ form still accumulated to a low level (5 to 10%), suggesting that Met cleavage was a limiting step (Fig. 3A). Notably, *E. coli* MetAP (*Ec*MetAP) is known to process substrates more efficiently when Ala occupies position 2 than when Ser is present (*32*). The aforementioned data indicate that AMPP2 displays an activity poorly sensitive to the nature of the penultimate cleaved residue (here Ser^2^). We therefore purified Rubisco from the ERBP expressing a Ser^2^Ala substitution variant of RbcL (RbcL-S2A) and compared its final processing to that of the WT. The S2A substitution led to the complete disappearance of the Met^1^ variant, favoring the Ala^2^ and Pro^3^ forms, and Pro^3^ emerging as the dominant species. This confirmed that Met cleavage of RbcL was limiting in the ERBP. Upon incubation in vitro with AMPP2, we observed a strong reduction in the accumulation of the Ala^2^ isoform (Fig. 3C, middle). Together, the data from both RbcL isoforms indicate that AMPP2 is active and highly specific, not only on model peptides but also on the fully mature assembled Rubisco complex in the bacterial reconstitution system. We lastly generated a P3A variant and observed that the Ser^2^ isoform accumulated in the ERBP, while the Ala^3^ isoform was not observed even upon the overexpression of AMPP2 (Fig. 3C, right). This is entirely consistent with both PepP and AMPP2 being unable to cleave a Ser^2^-Ala^3^ bond of RbcL and confirms the tight specificity of AMPP2 toward the Xxx/Pro cleavage previously observed in vitro (Table 1). Notably, the absence of the Met^1^ isoform in the P3A background further indicates that Pro at position 3 inhibits *Ec*MetAP-mediated RbcL cleavage, as reported earlier (*32*). Together, these findings demonstrate that AMPP2 is sufficient to drive the conversion of RbcL Ser^2^ to Pro^3^ both in vitro and in the ERBP.

### AMPP2 specifically processes RbcL in planta

To uncover additional substrates of AMPP2, we screened the 80 plastid-encoded ORFs in the *A. thaliana* proteome. Together, this analysis pointed to RbcL being the only plastid-encoded protein that specifically requires AMPP2 (Supplementary Text). To assess *AMPP2* function directly, we examined five independent insertion lines distributed along the At3g05350 gene map (Fig. 3D and fig. S5, A and B). Four of them (*ampp2-1*/*ampp2-2*/*ampp2-3*/*ampp2-4*) lacked detectable full-length *AMPP2* transcript (fig. S5C). N-terminomics profiling identified 1732 N-termini across all lines. RbcL was the only protein showing altered processing (Fig. 3E and data S1). In WT, the Pro^3^ isoform accumulates, whereas in AMPP2 KOs, mutant lines only the Ser^2^ isoform was detected, with >96% acetylation (e.g., *ampp2-4*: 98.7 ± 0.7%). The bottom-up analysis of the crude extract yielded the same conclusion (Fig. 3F, fig. S5D, and data S1). Notably, the N-termini of other putative substrates, such as PsaE, were unaffected in the *ampp2* background and remained identical to WT, confirming that they are processed by proteases other than AMPP2. These results support model 1, demonstrating that AMPP2 acts specifically after MetAP cleavage to generate the Pro^3^ form of RbcL.

Last, comparative label-free quantification (LFQ) of the proteome showed that overall protein abundance of adult plants was not impaired in the mutant lines compared to the WT (W3, fig. S5D). Aging *ampp2* plants grown under the same conditions (W7) showed a more pronounced decrease in plastid marker proteins (32, including Rubisco subunits), along with increased levels of stress and secretory protein markers (*14*) as revealed by Gene Ontology (GO) analysis (fig. S5E and data S2). Even without a visible phenotypical difference to WT at W3, the accumulation of GSTF2, ERD11, ERD12, PR1, PR2, and PLP2 at W7 suggests a molecular prelude to senescence.

We conclude that AMPP2 is the sole aminopeptidase responsible for Ser^2^/Pro^3^ cleavage of RbcL. In its absence, a plastid GNAT acetylates the remaining Ser^2^ of the RbcL isoform. AARE does not act on the *ac*-Ser^2^ terminal isoforms that accumulate in the *ampp2* background, consistent with its activity being limited to short peptides. The model 1 displayed in Fig. 1D therefore best explains RbcL N-terminal maturation, highlighting a competition between AMPP2 and plastid GNAT activity: AMPP2 generates the mature Pro^3^ form. Last, the loss of AMPP2 triggers stress-related pathways in older plants, potentially reflecting an early, presymptomatic stage of senescence.

### GNAT7 is the only acetyltransferase in charge of RbcL N-terminal Pro^3^ acetylation

**Fig. 4.**
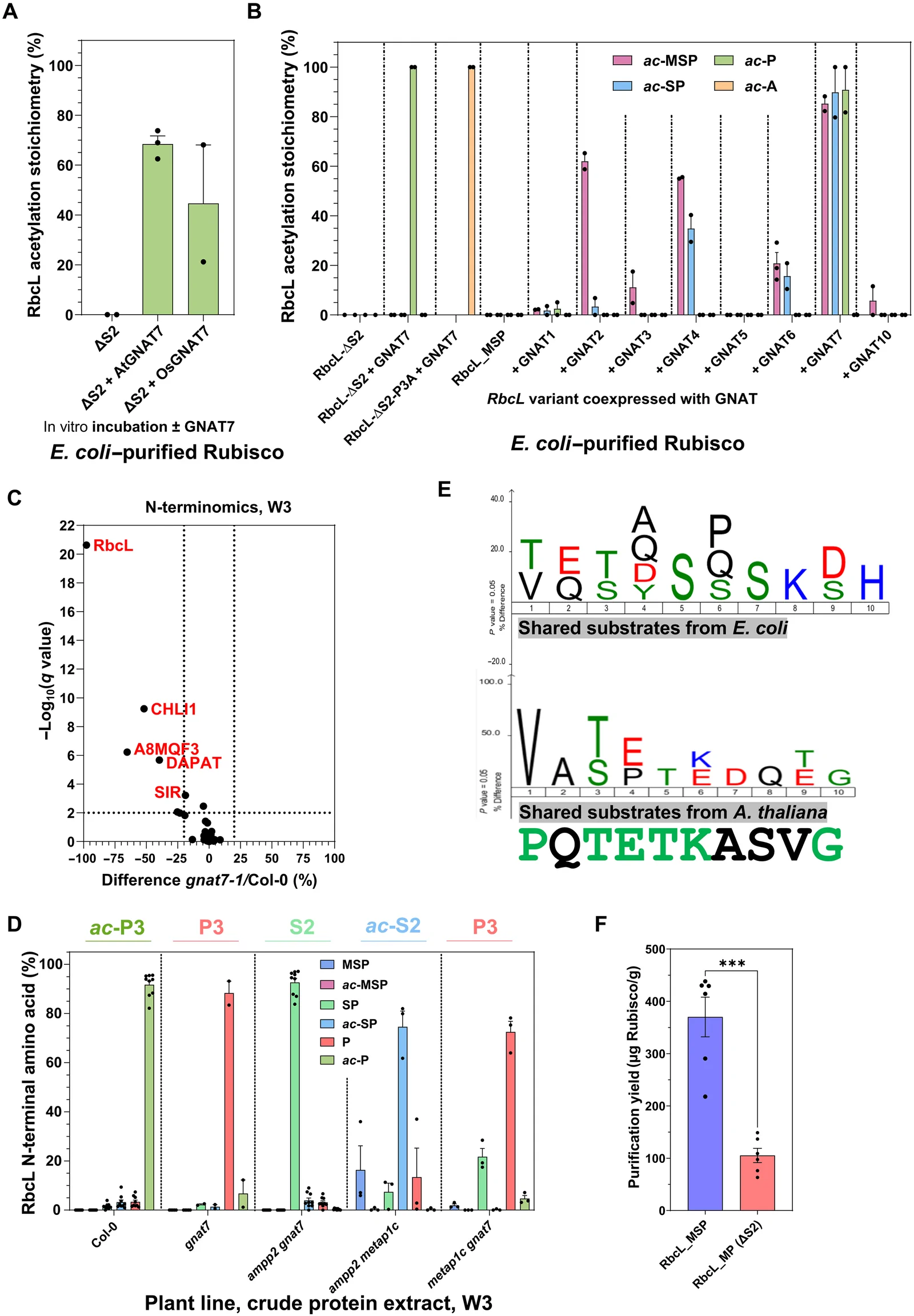
GNAT7 encodes a plastid acetyl transferase that specifically targets RbcL. (**A**) In vitro acetylation of 10 μM RbcL-∆S2 purified complex incubated in the presence of Ac-CoA (100 μM), and 1 μM AtGNAT7 or OsGNAT7. The effect was quantified by intact MS as in Fig. 3C (*N* = 3). (**B**) GNAT7 specifically acetylates RbcL in *E. coli*. Rubisco was purified from the extract arising from the ERBP described in Fig. 2B. MS was performed and data quantified as detailed in Fig. 3C; the relative amount of each deduced isoform is displayed (*N* = 2). See fig. S6B for quantified analysis of each N-terminal amino acid. (**C**) Impact of GNAT7 KO in the *gnat7-1 A. thaliana* line compared to WT (*N* = 4). The N-terminal protein acetylation yields from *gnat7-1* protein N-termini is displayed. (**D**) N-terminal residue distribution of RbcL in *gnat7* and in double mutant lines. Same analysis as in Fig. 2A but with crude extracts obtained from *gnat7* and from the three double mutant lines combinations resulting from the three crossing combinations of the specific catalysts of the RbcL modification pathway. The indications on the top reveal the prevalent amino acid as well as its modification status. (**E**) N-terminal pattern of GNAT proteins substrates as determined from different datasets (see section “Genetic evidence confirms that GNAT7 uniquely acetylates RbcL in planta”). Protein logo representations were displayed using IceLogo (*75*). Top: The *E. coli* protein modification pattern by GNAT7 as the well-fitted for RbcL-specific acetylation from (*23*). Middle: Shared N-terminal pattern of substrates of GNAT7 in planta from (C). Bottom: RbcL N-terminal sequence (see also Fig. 1A and fig. S1A). The sequence features common to those retrieved in the top and middle panels are colored in green. (**F**) Purifications yield of Rubisco from *E. coli* WT (MSP start) and MP-starting RbcL. Statistical analysis was performed using Welch’s test. ***P value = 0.0005.

To determine which of the plastid GNAT(s) (*23*) catalyze RbcL N-terminal acetylation, we combined the ERBP with the global acetylation profiling (GAP) assay (*33*). To avoid heterogeneity caused by partial PepP cleavage of Ser^2^, we first expressed an RbcL variant devoid of Ser^2^ (RbcL-∆S2). As expected, this construct produced a uniform Pro^3^-starting species (fig. S6). This approach enabled us to exploit the previously reported catalytic activity of all *Arabidopsis* GNATs in *E. coli* (*23*) to investigate their N-terminal acetylation specificity toward RbcL. Screening all eight *Arabidopsis* plastid GNATs revealed that only GNAT7 (At4g28030) efficiently acetylated Pro^3^ (fig. S6A). In *E. coli* extracts, GNAT7 yielded ∼80% Pro^3^ acetylation, which reached 100% when Rubisco chaperones were involved. This indicates that GNAT7, same as AMPP2 (Fig. 3A), can act before chaperone-mediated folding and assembly. The intact MS of purified Rubisco further confirmed the modification by GNAT7 in vitro: The nonacetylated RbcL-ΔS2 species (52,735 Da) gained a +42-Da shift (*N*-acetyl mass increment) upon in vitro incubation with purified GNAT7 and acetyl–coenzyme A (Ac-CoA) as a cofactor (Fig. 4A). N-terminal acetylation was specific for RbcL, as no modification was detected on RbcS. To test evolutionary conservation, the rice GNAT7 homolog (*Os*GNAT7) was heterologously expressed, and the protein was purified. *Os*GNAT7 efficiently acetylated RbcL in vitro (Fig. 4A), suggesting that GNAT7 orthologs in land plants share the specific ability to acetylate the N-terminal Pro of RbcL.

As mentioned above, upon expression in the ERBP, WT RbcL accumulated in the Rubisco complex as three different isoforms of similar intensity each corresponding to a start at position Met^1^, Ser^2^, and Pro^3^, respectively. Upon the expression of each of the eight GNATs with WT RbcL in the ERBP, we observed again that only GNAT7 meditated complete acetylation of Pro^3^. However, in *E. coli*, GNAT7 does not appear to be strictly specific for the N-terminal Pro^3^, as acetylation of RbcL was equally efficient when Pro^3^ was converted to Ala (Fig. 4B). This is consistent with the GAP assay data showing that GNAT7 acetylates a variety of protein N-termini of *E. coli* proteins including those containing Pro (*23*). Unexpectedly, GNAT7 also acetylated both the Met^1^ and the Ser^2^ forms of RbcL with similar high efficacy (Fig. 4B). The screening of the expression of the other seven plastid GNATs indicated that acetylation of Ser^2^ could only be obtained with GNAT4 and GNAT6. In addition to GNAT7, the acetylation of Met^1^ could be observed with GNAT2, GNAT4, and GNAT6. In both Met^1^ and Ser^2^ acetylation, GNAT7 appeared as the most efficient catalyst. GNAT1, GNAT3, GNAT5, and GNAT10 showed negligible N-terminal acetylation activity (Fig. 4B).

It was notable that the acetylation of Met^1^ or Ser^2^ in *E. coli* consistently reduced the abundance of the Pro^3^ isoform. This suggests that premature acetylation by certain GNATs can impair the normal sequential processing by MetAP and AMPP2. The effect was observed with GNAT2, GNAT4, and GNAT7, but not with GNAT6, implying that GNAT6 acts more slowly. Met^1^- and Ser^2^-acetylated RbcL species are rare in planta, indicating that their formation is prevented by the fine-tuned activities of GNATs, MetAP, and AMPP2 and/or that these improperly processed variants are disfavored in chloroplasts. Together, these data identify GNAT7 as the primary acetyltransferase responsible for RbcL Pro^3^ acetylation in the ERBP.

### Genetic evidence confirms that GNAT7 uniquely acetylates RbcL in planta

To test the role of GNAT7 in vivo, we analyzed two independent *A. thaliana* KO lines (*gnat7-1* and *gnat7-3*) using both classic MS investigation and N-terminomics at W3. In both lines, RbcL acetylation at Pro^3^ was reduced to <2% of WT levels (Fig. 4, C and D; fig. S6C; and data S3). No other processing isoform of RbcL was detected (including Ser^2^) as in the *ampp2* background. This finding establishes GNAT7 as the sole enzyme responsible for RbcL acetylation in planta.

Across both *gnat7* backgrounds, 1685 N-termini were identified. The quantitative determination of the acetylation yield compared to WT could be achieved for 610 and 423 of them in each line, respectively. Besides RbcL, only four nuclear-encoded, plastid-localized proteins—CHLI1 (At4g18480), A8MQF3 (At2g21385), DAPAT (At4g33680), and SIR (At5g04590)—showed significant reductions (>20%) in N-terminal acetylation (Fig. 4C and fig. S6C). Their yields of N-terminal acetylation were reduced to varying extents (15 to 60%) but not abolished, indicating a compensation of GNAT7 deficiency by other GNATs. Unlike RbcL, the modified residues of these targets were Val rather than Pro, suggesting that GNAT7 activity extends beyond RbcL but with weaker specificity. The Val residue is the most similar amino acid to Pro (i.e., side chain featuring three carbons). Sequence analysis revealed that GNAT7 substrates share a conserved N-terminal motif [V3A(S/T)(E/P)T(E/K)], reminiscent of the T5ETK element conserved in plant RbcL (Fig. 4E). This may explain the capacity of GNAT7 to acetylate Pro^3^ of RbcL with high specificity.

To conclude, our proteomic survey (∼700 plastid N-termini, ∼300 quantified in both WT and *gnat7*) shows that RbcL is the sole plastid-encoded GNAT7 substrate, whereas a small number of plastid-translocated proteins display partial dependence on GNAT7 and other GNATs. Overall, the total number of GNAT7 targets in plastids is unlikely to exceed a few dozen, with RbcL appearing to be the only specific target.

### GNAT7 is the sole catalyst of RbcL N-terminal acetylation in planta, irrespective of residue position

To investigate whether other GNATs could compensate for GNAT7, we crossed the *gnat7-1* and *ampp2-4* lines. MS-driven analysis revealed that RbcL accumulated predominantly as the unacetylated Ser^2^ isoform (Fig. 4D). This indicates that GNAT7 is the only GNAT that acetylates Ser^2^-RbcL. These in planta results differ from those observed in the ERBP, where the other *Arabidopsis* GNATs, such as GNAT4 or GNAT6, contribute to N-terminal acetylation of RbcL at Ser^2^ (Fig. 4A). It is known that this RbcL form is rarely detectable in WT (Fig. 1B). Nevertheless, neither GNAT4 nor GNAT6 could even partially compensate for the GNAT7 KO in planta.

To further probe GNAT7 activity, we crossed the *metap1c* KO line with either the *ampp2-4* or *gnat7-1*. In the *ampp2-4 metap1c* double mutant, RbcL accumulated mainly as a mixture of N-terminal Met^1^- and Ser^2^-variants (Fig. 4D), closely resembling those RbcL of the *metap1c* single mutant (Fig. 2A) and suggesting that Met^1^ removal is the primary determinant of the N-terminal fate of RbcL. The acetylation of Met^1^ of RbcL was detected, indicating that this event is possible but likely disfavored by the relative kinetics of MetAP1C and AMPP2 processing. In contrast, RbcL in the *gnat7-1-metap1c* background showed similar N-termini, but the acetylation of both Met^1^ and Ser^2^ was markedly reduced (Fig. 4D). Together, these genetic analyses demonstrate that GNAT7 is uniquely responsible for N-terminal acetylation of RbcL in planta, regardless of whether the mature protein begins with Pro^3^, Ser^2^, or Met^1^.

### Rubisco assembly in *E. coli* preferentially selects *ac*-Pro^3^ isoforms of RbcL

**Fig. 5.**
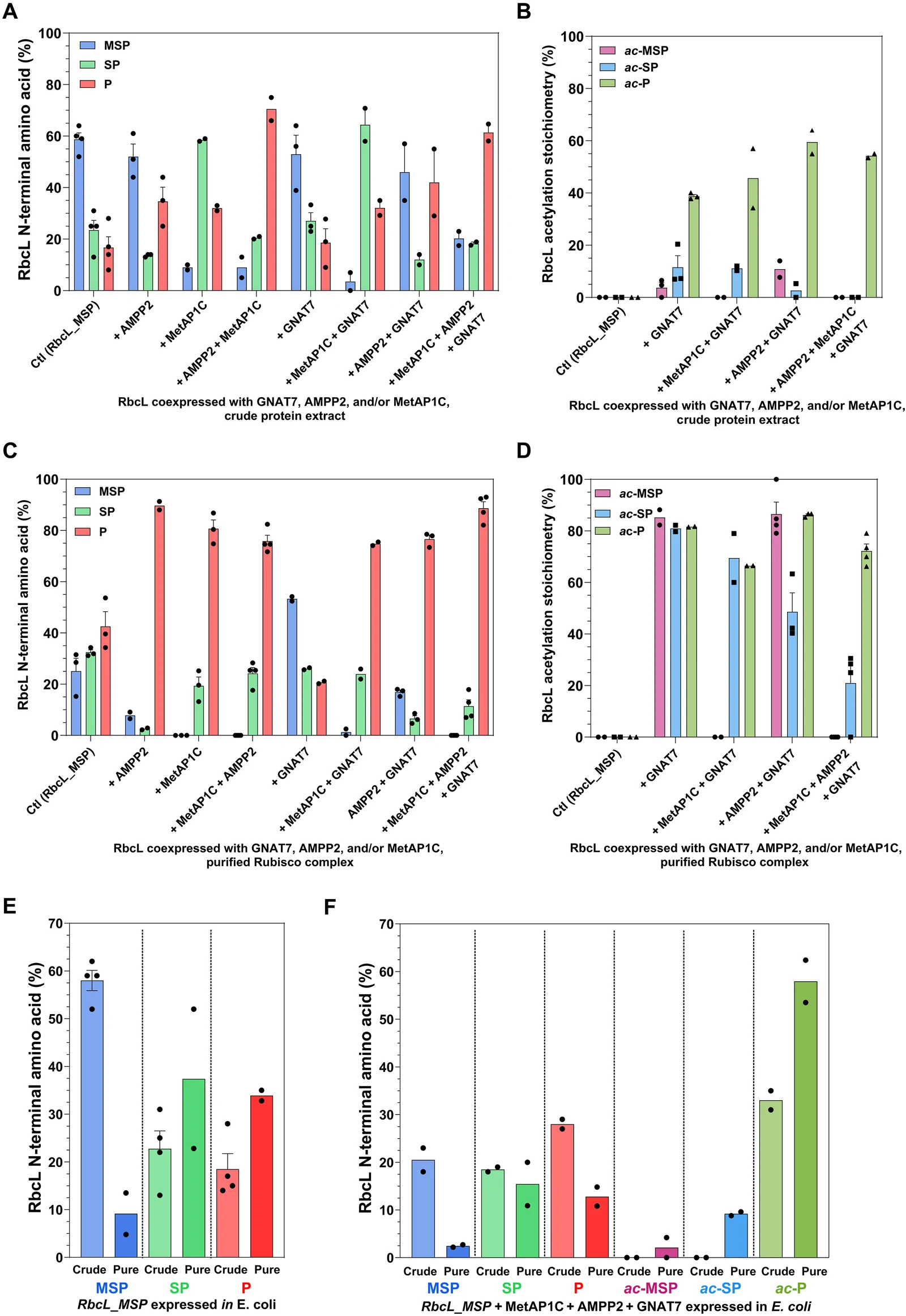
Complete processing of RbcL in *E. coli* upon co-expression of its dedicated modifiers and preferred accumulation of properly processed RbcL in the holoenzyme. (**A**) Nature of the N-terminal residue (acetylated or not) observed with RbcL isoforms in crude bacterial extracts (i.e., whatever the assembly state). Quantification was achieved by MS as in Fig. 1B (*N* = 3). (**B**) Acetylation yields of RbcL isoforms in the crude extract from panel A sample in which GNAT7 is associated (*N* = 2, except GNAT7, *N* = 3). (**C**) Same as (A) but with purified Rubisco from the same extracts (*N* = 3). Quantification was derived from intact mass analysis and quantified as in Fig. 3C. (**D**) Acetylation yields of RbcL isoforms in the crude extract from (C) sample in which GNAT7 is associated (*N* = 3). (**E**) Quantitative comparison of the nature of the N-termini accumulating in RbcL in both crude extract and purified protein samples. Quantification was derived identically after chymotrypsin cleavage of the RbcL band in either sample as in (A) and (B) (*N* = 4, crude; *N* = 2, pure). (**F**) Same as (E) but with samples from *E. coli*–expressing MetAP1C, AMPP2, and GNAT7 to favor N-terminal acetylation (*N* = 2).

As shown earlier, in the absence of any GNAT in BL21 cells expressing the WT RbcL, the RbcL N terminus could be observed as three different major isoforms (M1S2P3, S2P3, and P3). In the ERBP, we observed that Rubisco accumulation was significantly improved (i.e., ∼3.5-fold) with the RbcL variant with MSP compared to that with the MP(ΔS2) start (Fig. 4F). This suggests that the three N-terminal MSP codons were more favorable for enhanced translation of the mRNA than MP, although it eventually led to heterogenous N-termini (Fig. 2E). All seven coexpression combinations (three individual, three dual, and one triple) of MetAP1C, AMPP2, and GNAT7 with the complete Rubisco assembly machinery were next achieved in *E. coli* with the ERBP (Fig. 2B and fig. S7). The analysis of RbcL N-termini of each combination indicated that the triple combination was the best to promote *ac*-Pro^3^ as the dominant species in the crude extract (Fig. 5, A and B). The comparison of the N-termini of RbcL subunit-assembled complexes (Fig. 5, C and D) with those of the crude extract, which contains both unassembled and assembled subunits, revealed a notable bias. Assembled L8S8 consistently disfavored the Met^1^ isoform of RbcL and preferentially contained acetylated variants, predominantly *ac-*Pro^3^ when acetylation was allowed (Fig. 5, E and F, and fig. S7B). This enrichment strongly suggests that Rubisco chaperones selectively incorporate *ac*-Pro^3^ during assembly, while unacetylated or alternative N-terminal forms are excluded or destabilizing. The N-terminal pattern of purified Rubisco expressed together with the three modifiers tightly mimicked the actual pattern observed from plant-purified complexes (compare Fig. 5D with Fig. 1B).

Together with the mutant analyses, these reconstitution experiments explain why Rubisco accumulates in planta almost exclusively as *ac*-Pro^3^: The combined action of MetAP1C, AMPP2, and GNAT7 ensures correct maturation, and the assembly machinery itself further promotes the selection of the *ac*-Pro^3^ form. This dual safeguard combines precise N-terminal processing with the stability, abundance, and regulation of Rubisco.

### Loss of GNAT7 destabilizes the chloroplast proteome and triggers senescence programs

**Fig. 6.**
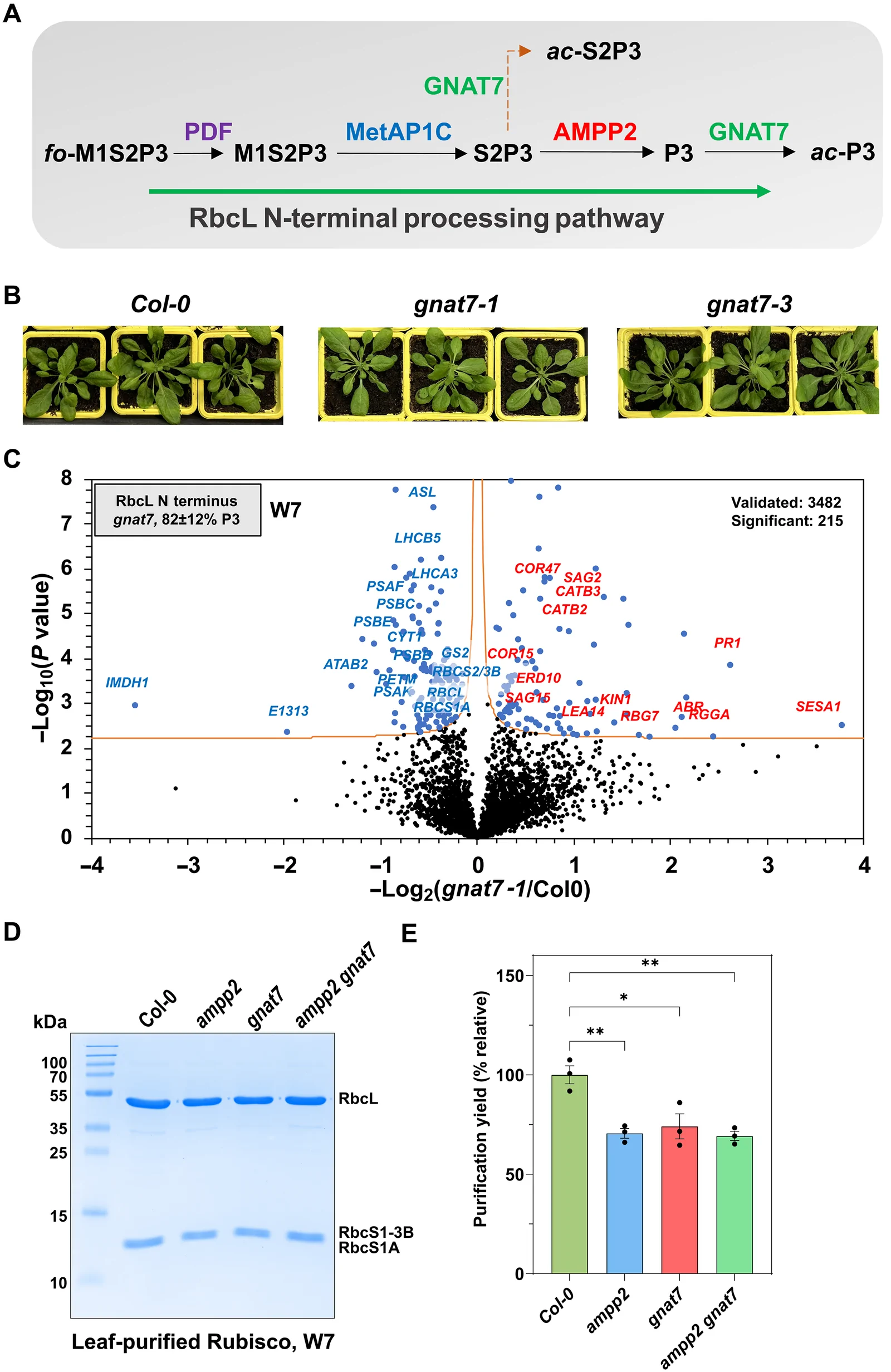
*GNAT7* inactivation causes stress-related proteomic alterations in *A. thaliana.* (**A**) Processing pathway of RbcL as evidenced in the current study. The various catalysts involved including MetAP1C are named with their respective gene names as used in the study. The minor route is tagged with a dashed line show that the reaction is possible but not favored in planta grown under standard conditions. GNAT7 is the sole GNAT that acetylates both Ser^2^ and Pro^3^. (**B**) Phenotypes at W3 of two independent *gnat7* mutant lines (*gnat7-1* and *gnat7-3*) compared to WT. (**C**) Impact of *GNAT7* inactivation on the proteome of line *gnat7-1* compared to WT. An LFQ experiment was conducted at W7 old shoots (*N* = 4). LFQ samples were analyzed on a timsTOF PRO 2 and data processed with FragPipe and Perseus. Proteins quantified in at least three replicates per group and either exceeding the significance threshold (orange line) or validated in the Student’s *t* test were considered significant. Proteins with significant increased accumulation in the mutant line are indicated with blue dots, and relevant entry names are displayed in blue (down) or red (up). A list of significant changes is provided in data S2. The complete set is provided in data S3. (**D**) SDS-denaturing PAGE (15%) of various purified Rubisco complexes reported in this study (1 μg/lane) from WT and the single and double *AMPP2* and *GNAT7* KO lines. The two types of RbcS (A/B) were resolved; their intensity is about the same as confirmed by intact mass analysis [see also (D) and fig. S3A for an example of a full mass range deconvoluted spectrum]. (**E**) Purifications yield (mg protein per g leaf) from *A. thaliana gnat7* and *ampp2* single and double lines. A value of 0.686 mg protein/g leaf was given to the WT.

Taken together, our previous data support the pathway illustrated in Fig. 6A. Consistently, *ampp2* plant lines lead to accumulation of *ac*-Ser^2^ RbcL while *gnat7* variants lead to unacetylated Pro^3^ isoforms. Under short-day conditions, same as the *ampp2* lines, the *gnat7* mutant lines displayed no obvious morphological differences to WT (W3; Fig. 6B). To assess whether molecular changes preceded visible phenotypes, we performed LFQ proteomics. At W3, *gnat7* plants showed only minor proteome changes (fig. S8A). We however noticed that the senescence marker SEN2 (At1g20620) accumulated together with oxidative stress markers. By contrast, at W7 the *gnat7* mutant line exhibited extensive proteome remodeling (Fig. 6C). About 6% (215 of 3482) of the quantified proteins were significantly altered in the *gnat7* background. The most notable reduction in content (129; data S2) concerned plastid-localized proteins (93; 78%), including very abundant proteins such as Rubisco (−15% for both RbcL and RbcS), glutamine synthase GS2 (−14%), and 5 subunits (including three core) of photosystem I (−40%), 8 subunits (including 5 core subunits) of photosystem II (−35%), 5 subunits of cytochrome b_6_f (−40%), and 12 components of light harvesting complexes (−34%). Although the average decrease in protein content was moderate, the pattern pointed to premature chloroplast proteolysis. Concomitantly, the accumulation of numerous stress- and senescence-related proteins increased significantly in the *gnat7* line (Fig. 6C and data S1), including oxidative and osmotic stress (proteins P5CS1 and GSTs) and cold stress (COR15, COR47, and KIN1) markers and vacuolar proteases (CATB, APA1, SAG2, and XBCP3). The strong reduction of the vacuolar cysteine protease inhibitor CYT1 suggests additional activation of autophagy and senescence signaling pathways (*34*). The GO profiling of the significant protein set analysis supported plastid and photosynthesis impairment together with response to stress, protein folding, and vacuolar and cytosolic induction (fig. S8B and data S1). Overall, these results indicate that GNAT7 deficiency in aging plants—similarly to the *ampp2* line but even more markedly—perturbs the chloroplast proteome and triggers stress and senescence responses in the cytosol and vacuole. We also observed that the N-terminal pattern of RbcL, including the complete loss of Pro^3^ acetylation, remained unchanged in *gnat7* at both W3 and W7 (Figs. 4C and 6C). This persistent N-terminal pattern suggests that the molecular phenotypes we detected arise directly from the RbcL processing defect caused by the KO, rather than reflecting a compensatory adaptation during aging.

### GNAT7- and AMPP2-dependent processing drives Rubisco accumulation and Rca activity

Because the loss of GNAT7 destabilizes the chloroplast proteome and triggers stress and senescence programs (Fig. 6C and fig. S8B), we asked whether Rubisco itself was directly affected in *ampp2* and *gnat7* mutants. To address this, we analyzed Rubisco accumulation, activity, and structural integrity in single and double KO plant lines.

**Fig. 7.**
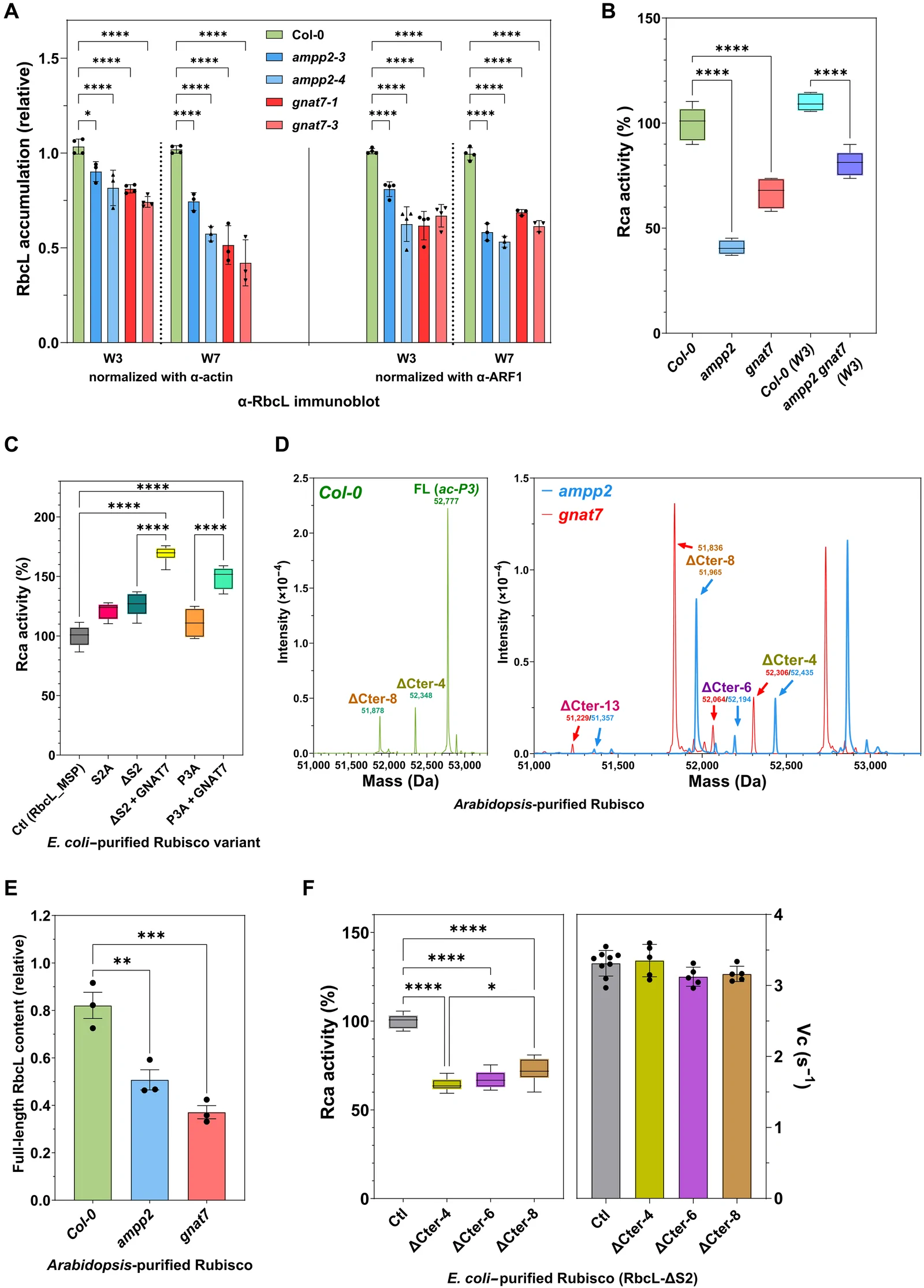
Rubisco isoforms from lines with defective N-terminal processing display unusual accumulation, cleavage and kinetic properties. (**A**) Accumulation of RbcL in leaves crude extracts at W3 and W7 (*N* = 4). A sample containing 6 μg of protein was analyzed by immunoblotting. Antibodies against RbcL, ARF1, and actin were used to obtain a ratio and normalize quantification of RbcL to a value of 1 for WT in each condition. (**B**) Rca activity of Rubisco purified from various lines devoid or not of the N-terminal processing pathway at stages W3 and W7 (*N* = 4). (**C**) Rca activity of Rubisco isoforms purified from *E. coli* cells expressing the indicated variants of RbcL—i.e., S2A (mix start of AP and P), ΔS2 (start on P), and P3A (start on SA)—and coexpressing or AtGNAT7. A value of 100 was given to WT RbcL (mix of M1, S2, and P3) (*N* = 6). (**D**) Intact mass pattern of RbcL from purified Rubisco at W7. The full-length (FL) and the main C-terminal deletions are displayed (ΔC-x, with x the number of deleted amino acids). Left: Pattern of WT is displayed (green line). Right: Both the *ampp2* and *gnat7* RbcL patterns (blue and red lines, respectively) were superimposed and displayed. The shift (129 ± 1 Da) between the two profiles corresponds to the additional N-terminal *ac*-Ser in the RbcL isoform from *ampp2* compared to *gnat7* (see Figs. 3E and 4, C and D). (**E**) Relative content (mg pure Rubisco/g leaf) of RbcL full-length isoform of each line is compared to WT [data from (D)]. Quantification was as in Fig. 3C (*N* = 3). (**F**) Kinetic properties of Rubisco with C-terminal deletions (*N* = 5). Each Rubisco form was purified from an MP (ΔS2) version expressed in *E. coli*. The Rca activity is shown on the left and the maximum velocity is displayed on the right.

Rubisco was purified to homogeneity from single and double *ampp2* and *gnat7* lines (Fig. 6D). We consistently observed that the yield of purified Rubisco complexes in the various KO lines were reduced by a factor of ∼30% compared to the WT (Fig. 6E). The native analysis of crude leaf extracts of single mutant lines revealed no intermediate, unassembled, or partially degraded complexes, indicating that RbcL was entirely found in the L8S8 complex (fig. S9, A and B). To independently quantify the actual changes in RbcL accumulation in the background of each plant line, immunoblotting was performed with two independent antibodies (actin and ARF1) as reporters for normalization. We observed in both *ampp2* and *gnat7* backgrounds a significant decrease of Rubisco abundance (Fig. 7A and fig. S9C). The decline was stronger in *gnat7* and induced more significantly during the older developmental stages. The decreased accumulation of proteins reached about 30%, on average, a value consistent with the LFQ quantification (Fig. 6C), and the Rubisco purification yields (milligram/gram fresh leaf) in each background.

We then asked whether altered N-terminal processing affects Rubisco function. To this end, we measured the associated kinetic parameters of the purified Rubisco from the different mutant lines (table S1) and observed no change of the *k*_cat_ values for ribulose-1,5-bisphosphate (RuBP); the *K*_m_ value for RuBP (*K*_m_^RuBP^) was not modified for Rubisco of *gnat7* but increased twice for *ampp2* single and the double KO lines. In contrast, Rca activity was significantly decreased in both N-terminal modifier-deficient Rubisco samples. The reduction was approximately twice as pronounced in protein purified from *ampp2* compared with *gnat7* (Fig. 7B). The effect was, however, less pronounced with the isoform purified from the *ampp2*-*gnat7* double mutant, suggesting that the presence of an unacetylated Ser residue at the N terminus may partially compensate for the loss of acetylation. To directly evaluate these findings, RbcL variants (ΔS2, S2A, and P3A) were engineered and each corresponding Rubisco variant was purified from the ERBP with or without GNAT7. These forms recapitulated the isoforms occurring in various plant genetic backgrounds. Rca activation was consistently enhanced when the N terminus carried Pro^3^ and when it was acetylated by GNAT7 (Fig. 7C). These data confirmed the observations made with plant-purified Rubisco.

We also characterized the Rubisco complexes from the various KO plant lines by intact MS. As expected, the canonical N-terminal processing pattern was detected. Unexpectedly, ∼20% of Rubisco in WT carried short C-terminal deletions (Fig. 7D, left). This observation was independent of the purification strategy. The population of truncated subunits increased dramatically in the mutants lines (Fig. 7, D and E). Last, to assess their functional impact and because truncated Rubisco versions could not be separated from the full-length isoform by purification, we separately expressed the major truncated isoforms (ΔCter-4, -6, and -8) in *E. coli* in RbcL-ΔS2 to obtain homogeneous Pro-starting N-termini (see Fig. 4B). These forms accumulated normally into L8S8 holoenzymes, indicating that Rubisco assembly was unaltered by such C-terminal deletions (fig. S9D). In addition, Rubisco complexes displaying these truncated RbcL isoforms could be purified to homogeneity. These complexes showed similar catalytic performances as the full-length holoenzyme for RuBP carboxylation but displayed significantly decreased performances for Rca activation (Fig. 7F).

Together, these results indicate that defects in N-terminal processing not only lower Rubisco accumulation and Rca activity but also promote the accumulation of C-terminally truncated isoforms. These truncated forms remain assembled and functional, albeit as compromised intermediates with reduced activation efficiency. These forms are most likely generated by carboxypeptidases such as CATB2/3 (*35*, *36*), which are up-regulated in the GNAT7 mutant backgrounds (data S1).

## DISCUSSION

### A dedicated machinery ensures precise N-terminal maturation of Rubisco

Our results establish that plant Rubisco is processed by a unique N-terminal processing pathway, involving sequential action of MetAP1C, AMPP2, and GNAT7 (Fig. 6A). The remarkable conservation of the N-terminal RbcL ORF (fig. S1), its final processing [this work and (*14*)] and the associated catalysts throughout the plant lineage [fig. S3 and (*22*)], suggest that the underlying mechanism is conserved in all plants. This pathway specifically generates the *ac*-Pro^3^ isoform of RbcL, which represents more than 90% of the mature enzyme in vivo. AMPP2 and GNAT7 emerge as highly specialized factors, with RbcL as their principal substrate. This substrate exclusivity is unusual for plastid proteases and GNATs, which typically act on multiple targets (*22*, *23*), and highlights how evolution has dedicated two enzymes to secure the maturation of a single, but exceptionally abundant and essential, protein. The conservation of Pro^3^ and its acetylation is particularly notable, as Pro is the only proteogenic residue with a secondary amine. However, the functional significance of the conserved Pro^3^ acetylation in RbcL has so far remained unclear. Structural models suggest that the N-terminal tail of RbcL is disordered and not stably engaged in the L8S8 complex (*37*), yet our data reveal profound functional consequences when processing is perturbed, pointing to regulatory rather than structural constraints.

### Chaperone-assisted Rubisco assembly favors properly processed N-terminal forms

Our current data taking advantage of the *E. coli* reconstituted system (*28*, *29*) indicate that appropriate N-terminal Proline acetylation favor chaperone mediation and RbcL incorporation into the final active fully assembled holoenzyme, whereas the unprocessed subunits are left as unassembled subunits. In planta, this selective assembly likely contributes to the decreased accumulation of Rubisco in *ampp2*, *gnat7*, and double mutants. The progressive drop in holoenzyme abundance during aging suggests a dual mechanism: the inefficient assembly of noncanonical isoforms combined with premature degradation of unstable complexes. This is consistent with prior work showing that Rubisco assembly intermediates are rapidly cleared if they are not stabilized by chaperones (*28*). Rca interactions involving N-terminal modifications drive Rubisco enhanced activity

Rca is a hexameric complex that restores Rubisco inhibition by promoting the release of tightly bound sugar phosphates from the active site of Rubisco (*38*). Rca functions as a Rubisco-specific chaperone whose ATPase activity enables it to remodel the holoenzyme itself. Quantitative proteomic studies revealed that Rca can accumulate to high levels in *Arabidopsis* leaves (*39*). Although the Rca-Rubisco binding constant lies in the micromolar range (*40*, *41*), the high concentrations of each of the two partners in the plastid stroma shift the equilibrium toward the bound state within the compartment. Structural data obtained with the Rubisco:Rca complex from *Nostoc* sp. PCC 7120 (a cyanobacterium which RbcL subunit is highly similar to that of plants) indicate that the binding surface of Rubisco on Rca involves both the N- and C-terminal sides of RbcL (*40*). RbcL deletions as short as two amino acids (e.g., P3Q4) and analysis of Rubisco variants with unacetylated RbcL generated via the ERBP demonstrated that the N-terminal region is critical for Rca:Rubisco interaction (*42*). In the absence of Rca, this domain appears disordered as no crystallographic density is observed upstream of Ala_9_ in any available Rubisco structure (fig. S10). It is located on the outside of the complex and is fully available for interaction with the Rca ring (fig. S11). Our data indicate that aberrant N-terminal processing leading to short extensions or deacetylation of RbcL also significantly alters Rca activase action. In agreement, *Nostoc* RbcL starts on S2YAQTET, while plant RbcL starts on *ac*-P3QTET, and the major interactions occur on the A4-T8 tetrapeptide (*40*, *42*). These residues correspond to Pro^3^-Thr^7^ of the plant forms. The *ac*-Pro^3^ moiety of RbcL might be involved in tight contacts with residues of Pore-loops 1/2 (fig. S10). It is known that the N terminus of RbcL is threaded through the axial pore of the Rca hexamer and thus modifications of the N terminus can affect this process.

### RbcL: A Pro/N-degron pathway target protected by N-terminal acetylation

In *Saccharomyces cerevisiae*, the Pro/N-degron pathway was identified and shown to target proteins bearing nonacetylated N-terminal prolines (*43*). The N-recognin Gid4, a subunit of the oligomeric glucose-induced degradation ubiquitin ligase, recognizes these N-terminal prolines and directs the corresponding proteins for degradation. It has also been reported that yeast AMPPs (displayed in fig. S3B)—including AMPP1 (Fra1), the cytosolic homolog of plastid AMPP2, and Icp55, the mitochondrial homolog of PepP—are involved in the Pro/N-degron pathway (*44*). By trimming residue 2 of several proteins, after Met^1^ cleavage by MetAPs, these AMPPs unmask an N-terminal proline, thereby initiating degradation via the Pro/N-degron pathway.

The RbcL processing mechanism described in the present report is remarkably similar, yet it includes an additional step—proline N-acetylation by GNAT7—which prevents RbcL from premature degradation. A protective role of N-terminal acetylation by NatA in preventing degradation and promoting protein stability has recently been demonstrated in the cytosol of plants (*45*). Moreover, it has been reported that the cytosolic N-degron machinery in plants differs substantially from that of yeast and cannot be directly extrapolated (*46*). Nevertheless, it is remarkable that RbcL appears to use a strategy reminiscent of the yeast Pro/N-degron system.

### C-terminal trimming as a marker of rubisco turnover

Plant RbcL subunits are highly conserved (∼95% identity), but the last 15 C-terminal residues are the least conserved and form an extended strand at the outer part of the complex (*37*, *47*). *A. thaliana* is one of the plants whose RbcL sequence has four additional residues compared to the classical length of 475 aa residues of the unprocessed protein (fig. S10B). The crystal structure of *A. thaliana* Rubisco does not show the last four residues, as if they were flexible (*37*). As mentioned, the C terminus of RbcL was also shown to interact with Rca (Fig. 7F). In this work, we unexpectedly observed that the C-terminal peptide of about 20% of RbcL subunits is trimmed in WT leaves. This observation was only possible thanks to intact mass analysis of the complex and would otherwise have remained undetected, as no significant migration differences were observed in either denaturing or native PAGE, including the various C-terminal deletion variants we produced in *E. coli*. This C-terminal trimming has never been observed in any of the numerous Rubisco complexes produced in *E. coli* during this study. These findings suggest that the phenomenon is physiologically relevant and highlight the sensitivity of Rubisco to progressive trimming of its C-terminal regions, which are accessible to proteases such as carboxypeptidases.

One can exclude that the *ac*-Pro^3^ N terminus directly protects the N terminus from trimming as when the N terminus of RbcL was altered in *gnat7* and *ampp2* KO mutants, we did not observe any N-terminal cleavages. Whereas the N terminus remained unaffected, C-terminal deletions accumulated to higher levels, predominantly in even-numbered deletions (4, 6, and 8 deletions) along with additional longer deletions. In this context, we hypothesize that the N-terminal side is protected mostly as it is largely engaged in the interaction with Rca, preventing easy access to protease. When the interaction with Rca is lowered as a result of alterations of the N-terminal processing, the residence time of the complex is decreased, and the complex is likely more prone to damages including oxidation due to the strongly oxidative environment of the stroma. The damaged proteins therefore increase and are more prone to degradation.

**Fig. 8.**
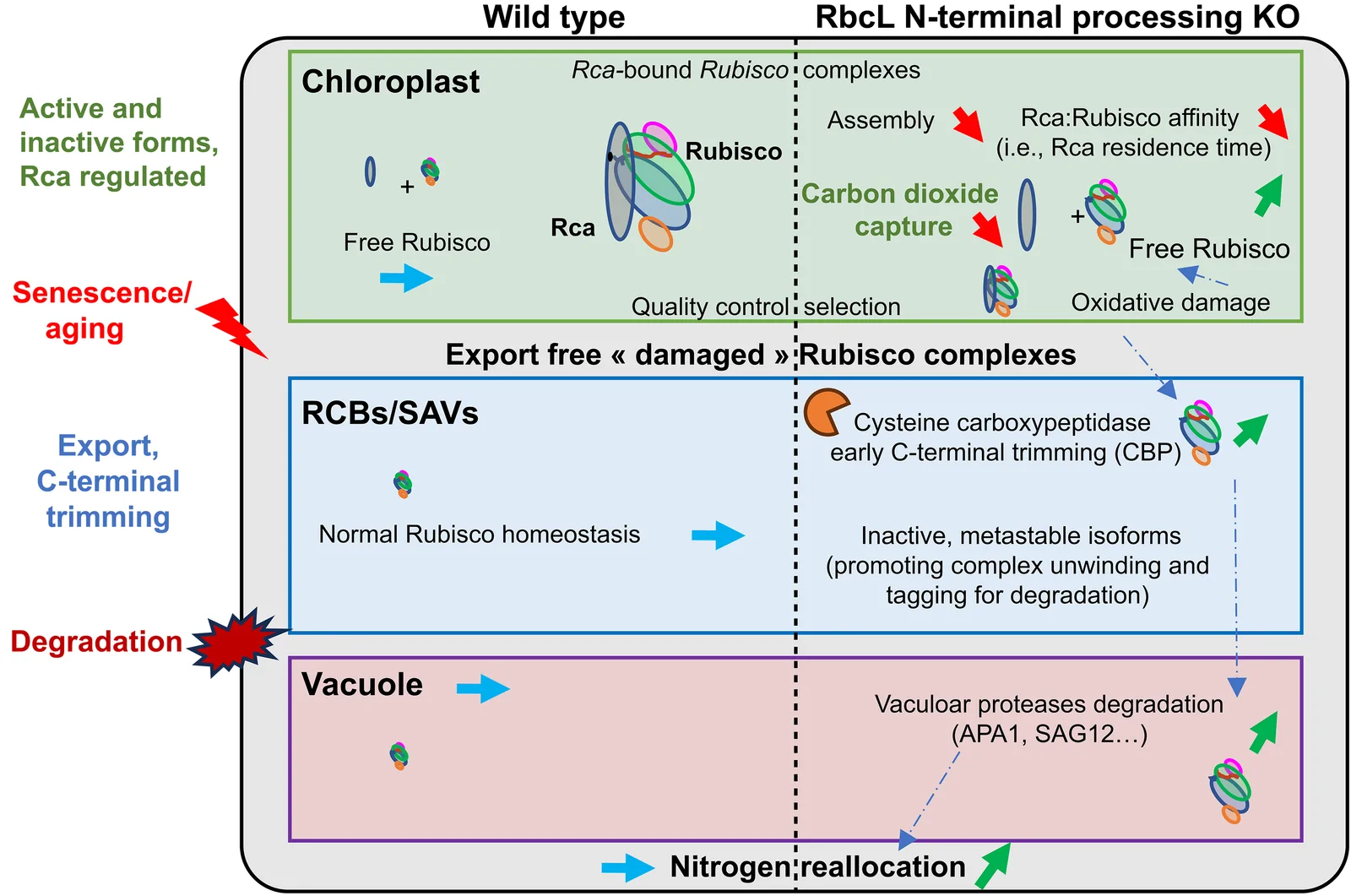
Proposed mechanism linking reduced RbcL N- and C-terminal interactions with Rca to enhanced degradation in senescence. Model of the functional impact of impairment of the RbcL N-terminal pathway in relationship with Rubisco activity, degradation and ageing. Cartoon showing the different compartments where Rubisco particles can be retrieved in plants and the effects observed in AMPP2 or GNAT7 KO lines. The left part relates to the WT and the right to the KO lines. Red arrows indicate down-regulated, green arrows indicate up-regulated, and light blue arrows refer to normal regulation.

As there is no carboxypeptidase (CBP) described so far in plastids, the observed cleavages might instead originate from chloroplast protein-trafficking vesicles, such as Rubisco-containing bodies (RCBs) or senescence-associated vacuoles (SAVs), which protrude from the plastid and target damaged holoenzymes to degradation (*48*–*50*). SAVs and RCB contain CBPs and are eventually internalized in the vacuole (*51*). One could therefore consider the C-terminally deleted isoforms as early markers of Rubisco complexes tagged for degradation. The acidic environment of RCBs and the associated protease content both favor CBP cleavage of RbcL (*52*). Our LFQ proteomic data in the *gnat7* background show that other marker of early senescence and proteases are strongly accumulated. Because there is neither RuBP nor Rca in RCBs, the C-terminally cleaved Rubisco should be considered as inactive in these small spherical double-membrane structures. This complements the observation that N-terminal processing forms in *ampp2* or *gnat7* lines account for an ∼30% decrease of the steady-state Rubisco content, a reduction that progressively increases with plant aging. Considering both the overall RCB content and the decrease in the pool of active Rubisco (i.e., only full length), it can be concluded that alterations in RbcL N-terminal processing reduce the plant’s Rubisco catalytic capacity by at least threefold. A schematic summarizing this model is shown in Fig. 8.

### Why does RbcL start on an MSP and not simply on an MP codon?

An intriguing discovery of our study involves the demonstration that deletion of Ser^2^ of RbcL, allows easier processing to *ac*-Pro^3^ with MetAP1C and GNAT7. One can wonder why the presence of the Ser^2^ codon is conserved in plants, especially as its removal requires to express two dedicated aminopeptidases, MetAP1C and AMPP2. We noticed however that when producing the ΔS2 variant in *E. coli*, the accumulation of Rubisco was significantly reduced to as little as 30% of the WT. Unexpectedly, the U_+4_ of Ser codons versus C_+4_ of Pro codons are not known to influence merely the translation efficiency in *E. coli* (*53*). In any case, if this enhanced translation also occurs in plastids—which is very likely given the close similarity of all characteristics related to translation initiation in the organelles of plants and in bacteria (*54*)—then it could significantly contribute to protein synthesis, allowing Rubisco to reach the high concentration required in leaves to sustain plant photosynthesis. Another plausible reason why the Ser^2^ codon was kept through evolution could also be linked to a specific role of the *ac*-Ser^2^ starting variant. In our study, we have not identified physiological conditions in which the *ac-*Ser^2^ isoform shows significant accumulation. However, *ampp2* KO lines are functional and the resulting Rubisco complex displays remarkable features including a higher *K*_m_ value for RuBP carboxylation (*K*_m_^RuBP^), a lower Rca activation ability together with a reduced accumulation. Such a high *K*_m_^RuBP^ makes the catalytic properties of plant Rubisco similar to those of cyanobacterial forms (*55*), with moderate response to a higher metabolic load. Although millimolar concentrations of RuBP in the stroma typically saturate Rubisco under steady-state high-light conditions, the observed increase in *K*_m_^RuBP^ could become limiting during photosynthetic induction or under fluctuating light, when substrate availability is reduced. It is therefore most plausible that some plants or some physiological conditions, under defined circumstances, might down-regulate AMPP2 gene expression and promote the *ac-*Ser^2^ version of RbcL as a major isoform. It should be mentioned however that complete turnover of Rubisco from one isoform to another might be delayed for several days at least because of its strong abundance and stability. This would complicate identification of the necessary conditions in a screening approach.

The two hypotheses above are not mutually exclusive and could explain why the MXP start has been selected over MP and kept throughout evolution. Now why Ser and not another residue? Keeping a small residue (Ala, Cys, Gly, Pro, Ser, Thr, or Val) at this position allows MetAP to cleave and allows another aminopeptidase like AMPP2 to cleave the X-Pro bond. Most AMPPs display preference for N-terminal large residues, as confirmed with Met in our current study with AMPP2 (Table 1). Among MetAP-compatible small residues, AMPP2 exhibits lower efficiency on Ala-starting sequences compared with those starting with an N-terminal Ser. Therefore, the use of residues at position 2, such as Ser or Val (as observed in green algae), which have medium-sized side chains, could represent a compromise that allows optimal coupled cleavages by both MetAP1C and AMPP2.

### Evolutionary convergence of RbcL and actin processing in plants and animals

The specialization of AMPP2 and GNAT7 for Rubisco parallels the situation of actin in animals. Actin is another very abundant intracellular protein on Earth, found in all eukaryotes and in all cell types (1 to 5%) with tissues accumulating more than 10% of actin (*56*). Unlike other actin isoforms, muscle actins of animals (i.e., isoforms α, β, and γ) feature an unusual N-terminal processing pathway showing notable similarity to that of RbcL as it involves N-terminal acetylation of an unusual residue (here, Asp or Glu) also at position 3. The cysteine protease ACTMAP acts as the specific actin maturation aminopeptidase removing the N-acetylated amino terminal residue (*57*). This residue may originate or not from cytosolic MetAP cleavage of the N-terminal methionine, and it is N-acetylated first by the classic NatA or NatB cytosolic N-acetylation machinery (*58*). After ACTMAP cleavage, the unmasked residue (i.e., the second or third residue) systematically corresponds to a negatively charged Glu or Asp residue. The final acetylation on Glu/Asp residues involves a dedicated NAT, NAA80 (also known as NatH). ACTMAP impairment is associated with abnormal muscle physiology, while NAA80 depletion shows cytoskeleton disorganization. As expected, this animal-specific processing pathway in muscles does not occur in plants, which display neither ACTMAP nor NAA80 (*18*). In parallel, plants display two analog catalysts, AMPP2 and GNAT7, for complete processing of Rubisco, a complex that animals do not have.

Muscles are associated with the unique ability of animals of locomotion on terrestrial environments and the search for food supply. Our current study reveals that the specific N-terminal pathway of RbcL also contributes to adaptation of plants to terrestrial conditions such as its use as a major nutrient storage. Together, this similarity in the maturation processing of actin and Rubisco reveals a notable case of evolutionary convergence. Thus, animals and plants have both developed specialized N-terminal modification pathways targeting their most abundant and essential proteins highlighting a shared adaptive strategy finely tuned to their respective life modes and environmental demands. This underlines how crucial and central N-terminal processing mechanisms are in the overall frame of proteostasis (*25*).

## MATERIALS AND METHODS

### Chemicals

All chemicals were purchased from Sigma-Aldrich unless otherwise stated. All peptides (>95% purity) were purchased from Genscript Biotech Corporation (Rijswijk, Netherlands).

### Bacterial strains

BL21Star(DE3) {F^–^ *ompT gal dcm rne131 lon hsdS_B_*(*r_B_*^–^*m_B_*^–^) λ(DE3 [*lacI lacUV5*-*T7p07 ind1 sam7 nin5*]) [*malB*^+^]_K-12_(λ^S^)} were used for Rubisco expression. BL21pLysS or BL21(DE3)pLysS [F- *omp*T *hsdS_B_*(*r_B_*^–^*m_B_*^–^), *dcm*, *gal*, λ(DE3), pLysS, Cm^r^] and Rosetta(DE3) BL21 derivatives [F- *omp*T *hsdS_B_*(*r_B_*^–^*m_B_*^–^) *gal dcm* (DE3) pRARE, Cm^r^] were used for all other protein expression as indicated below. The LG-2 strain features a complete PepP gene (*pepP*) deletion (*30*). LG-2 is derived from parental strain BW25113 [*lacI*^+^*rrnB*_T14_ Δ*lacZ*_WJ16_ *hsdR*514 Δ*araBAD*_AH33_ Δ*rhaBAD*_LD78_ *rph-1* Δ*(araB–D)567* Δ*(rhaD–B)568* Δ*lacZ4787*(::*rrnB-3*) *hsdR514 rph-1*]. LG-2 displays both WT *lacI* and the *araC* allele and could be used as a result for coexpression of pBAD33-AtRbcLS (*28*) (CmR; p15A) under arabinose control and pTrc99A-AMPP2 under isopropyl-β-d-thiogalactopyranoside (IPTG) induction (fig. S4B).

### Nomenclature and gene identification of *Arabidopsis* MetAP proteins

MAP is already a well-established abbreviation for (i) “mitogen-activated protein,” particularly in the context of MAP kinases, which are widely studied as signaling markers, (ii) “microtubule-associated proteins” (such as tau, MAP1, and MAP2), and (iii) other protein groups including “membrane-associated proteins.” To avoid ambiguity, gene-specific identifiers in animals have logically adopted the form METAP, with the suffix distinguishing individual isoforms or genes. This convention separates the general enzyme category (MetAP) from precise genetic and protein nomenclature, thereby ensuring clarity in both the literature and databases. In this report, we apply the same nomenclature to the *A. thaliana* genes and proteins. Another source of confusion has been the conflicting nomenclature for two *A. thaliana* MetAP proteins analyzed in this study. Specifically, UniProt lists Q9FV52 as MAP1B (gene MAP1B, At1g13270) and Q9FV51 as MAP1C (gene MAP1C, At3g25740), in agreement with (*19*). However, the Arabidopsis Information Resource assigns both MAP1B and MAP1C to these two distinct genes, thereby creating ambiguity in the correct identification of the corresponding gene products. To resolve this, we here redefine At1g13270 as encoding protein MetAP1C (gene METAP1C) and At3g25740 as encoding protein MetAP1B (gene METAP1B), ensuring unambiguous nomenclature for subsequent reference.

### Plasmids and gene constructs

A list of constructs used throughout this study is available as table S2.

A list of primers is provided in tables S3 and S4.

#### AMPP2

The complete ORF clone from AMPP2 (At3g05350) cloned in pUNI51 was obtained from Arabidopsis Biological Stock Center (ABRC) (U21738). The ORF devoid of the cTP (631 amino acids, 70 kDa) in pET15a to yield pET15a-AMPP2 featuring an N-terminal His tag followed by a tobacco etch virus (TEV) cleavage site. pTrc99A-AMPP2 was obtained by cloning the short ORF between the Nco I and Xba I sites of pTrc99A.

#### GNATs

AtGNAT7 (At4g28030) full-length cDNA was obtained from ABRC (U13113/GenBank ID: AY133564). Codons 37 to 274 were cloned in both pET28 and pETM41 as a fusion with His6 tag and maltose binding protein, respectively. pET28 and pETM41-GNAT7 derivatives were used for protein expression purposes. NB codons 54 to 268 led to a nonfunctional enzyme.

*Oryza sativa* GNAT2 (OsGNAT2) expression vector was described in (*59*). The ORF sequence of OsGNAT7—the closest rice homolog to AtGNAT7 (*22*)—was synthesized de novo from EMBL ID: B1015H11.141 (UniProt ID: Q6Z612). Codons 67 to 291 corresponding to the GNAT domain devoid of TP were inserted in pET28 and pETM41 expression vectors.

#### Rubisco expression with chaperones

All plasmids used for expression and purification of active Rubisco from *E. coli* are described in Fig. 2B and table S2. Several were previously described (*28*, *29*).

#### *Rubisco activase Rca*β

Rubisco activase Rcaβ isoform from *A. thaliana* (At2g39730) was amplified from pUNI51 clone (ABRC, U13656) encoding the α subunit in a pET15 vector fused to an N-terminal 6xHis tag. We deleted codons 1 to 58 corresponding to the chloroplast targeting peptide and replaced the last 36 codons by the GKTEEKEPSK codons specific of the β isoform [P10896.2; (*60*)].

#### pACYC-Duet derivatives

pACYCDuet-1 (Novagen) was used as a compatible plasmid (p15A replication origin, Cm^R^) allowing to complement BL21 derivatives with additional overexpressed genes. Combinations of AMPP2, GNAT7, and/or MetAP1C expression units under control of the T7 promoter were cloned in pACYC-Duet as detailed in Fig. 2B for complementation experiments in *E. coli* BL21 cells. DNA inserts were from the various pETM41-GNATs, pET15a-AMPP2, and from pM1B (*19*). The NEBuilder HiFi DNA Assembly Master Mix (NEB) was used for cloning AtAMPP2, AtMetAP1C, AtGNAT2, AtGNAT3, AtGNAT4, AtGNAT6, and OsGNAT2. NcoI–Bam HI subcloning was used for AtGNAT1, AtGNAT5, AtGNAT7, AtGNAT10, and OsGNAT7.

### Plant material

#### Growth conditions

Seeds of *A. thaliana* (Columbia ecotype; *Col-0*) were surface-sterilized and sown on 0.5× Murashige and Skoog (Sigma-Aldrich) medium supplemented with 0.8% agarose and 1% sucrose in petri dishes. Seeds were imbibed for 2.5 days in the dark at 4°C for vernalization. Petri dishes were then transferred to a growth chamber (21°C, 16 hours of daylight; 60% hygrometry; light intensity of 100 μmol photon m^−2^∙s^−1^) for 5 days. *Arabidopsis* lines were then transferred on soil and grown under greenhouse conditions (24°C/18°C, 16 hours daylight) for seed propagation. For all other analysis in this report, plants were grown in short days in plant cabinets (21°C, 8 hours of daylight; 60% hygrometry; light intensity of 117 μmol photon m^−2^∙s^−1^). Plant growth kinetics were assessed as a function of weeks (Wx), where “x” corresponds to the number of weeks of incubation at 21°C after the start of growth incubation. Samples were usually taken at W3 and W7 unless otherwise stated.

#### Plant lines

All seeds were from the ABRC (https://abrc.osu.edu/). The AtMetAP1C (At1g13270; 10 exons) KO T-DNA insertion line described in this study corresponds to *metap1c-1* (SALK_108786) with insertion in intron 7. The AtMetAP1B (At3g25740; 10 exons) KO T-DNA insertion line corresponds to *metap1b-1* (SALK_004655) with insertion in intron 2. The AtMAP1D (At4g37040; nine exons) KO T-DNA insertion line corresponds to *metap1d-1* (SALK_064678) with insertion in intron 2.

The AMPP2 gene (At3g05350; 13 exons) KO T-DNA insertion lines described in this study correspond to *ampp2-1* (SALK_122528), which insertion lies in intron1, *ampp2-6* (SALK_002058) in exon 6, *ampp2-2* (SALK_050757) in exon 10, *ampp2-3* (SALK_088209C) in intron 11, and *ampp2-4* (SALK_136128) in exon 13 (fig. S5, A to C). Several primers pairs were used to identify the T-DNA insertion in each line (table S4). Transcript absence was probed by real-time polymerase chain reaction (RT-PCR) with three independent primer pairs (table S4) showing no full-length transcript accumulation *ampp2-1/2/3/4* lines (fig. S5C). Note that At3g05360 is an ORF made of a single exon lying within AMPP2 intron 1 and which is inactivated only in the *ampp2-1* background (fig. S7A). At3g05360 (RLP30/Q9MA83) is described as a receptor for microbe-associated molecular patterns that induces a BAK1-dependent basal immune response to necrotrophic fungi. Next, an insertion in line SALK_050757 *ampp2-2* was reported in a second gene (At1g56380) encoding a mitochondrial transcription termination factor family protein. Last, line *ampp2-5* (SALK_075759) lies at the extreme end of the 3′ untranslated region (3′UTR) of the mRNA (cDNA sequence GenBank Id AY079018 and BT001080) and is not an *AMPP2* KO line, as evidenced by RT-PCR (fig. S5C) and further confirmed by proteomics (Fig. 3B and data S2). The *ampp2-3* and *ampp2-4* lines were selected for further characterization of AMPP2 KO-associated phenotypes. As line *ampp2-3* displays a 2-day longer germination time than the other lines for an unknown reason, line *ampp2-4* was used in analysis relative to the AMPP2 KO background. *ampp2* corresponds to *ampp2*-4 unless otherwise stated.

GNAT7 (At4g28030) T-DNA insertion KO lines correspond to *gnat7-1* (SAIL_273_E06) lying in exon 1 as reported previously (*61*) and *gnat7-3* (WiscDsLox4B08) in intron 2. For poplar leaf preparation, we used an interspecific hybrid between *Populus tremula* × *Populus alba*, and samples were taken at W19.

### Protein sample preparation, gel electrophoresis, and immunoblotting

Protein extracts were prepared as described (*59*), and crude protein concentrations were determined with the Bradford assay using an Infinite M Nano microplate reader (Tecan). *A*_280_ (absorbance at 280 nm) measurements of purified proteins were performed on a NanoDrop 2000 spectrophotometer (Thermo Fisher Scientific), and protein concentrations were calculated using the theoretical extinction coefficients of each protein (https://web.expasy.org/protparam/). An extinction coefficient of 103,250 M^−1^·cm^−1^ was used for one Rubisco heterodimer.

Native polyacrylamide gradient mini protein gels (NuPAGE, 4 to 12%, 1.0 mm, 15-well; Thermo Fischer Scientific) were run in tris-glycine buffer at 100 V for 30 min, and then the voltage was turned to 150 V for 3 hours. The NativeMark unstained protein standard (Invitrogen) was used as the ladder. SDS-denaturing protein electrophoresis was performed on 10 or 12% polyacrylamide gels in standard tris-glycine buffer (Mini-Protean TGX, Bio-Rad) at 150 V. The PageRuler Plus prestained protein ladder was used as the size standard (Thermo Fischer Scientific). Gel staining was achieved with InstantBlue Coomassie Protein Stain (Abcam).

Immunoblotting was achieved as described (*62*). Rabbit polyclonal antibodies for actin, RbcL, and ARF1 (AgriSera, AS13 2640, AS03 037, and AS08 325) were used at dilutions of 1:5000, 1:5000, and 1:1000, respectively. Anti-rabbit antibodies coupled to horseradish peroxidase (A0545, Sigma-Aldrich) were used at a dilution of 1:10,000 for 2 hours. An enhanced chemiluminescence kit (Cytiva) was used as a detection reagent. Immunoblots were imaged on a Chemidoc Touch Imaging System (Bio-Rad).

### Protein purification

Recombinant benzonase nuclease (Sigma-Aldrich, E1014) was used for removing nucleic acids. His-tagged TEV protease was prepared as described (*63*) and used, when indicated, to remove the N-terminal His tag from recombinant proteins (1 mg/15 mg purified protein). TEV protease was subsequently removed from the protein sample by nickel-affinity chromatography, as described below.

#### AMPP2 purification

The ORF devoid of the cTP (631 amino acids, 70 kDa) in pET15a to yield pET15a-AMPP2 featuring an N-terminal 6xHig tag, followed by a TEV cleavage site. The protein was expressed in BL21Star(DE3) bacteria upon IPTG induction (0.5 mM) at an optical density at 600 nm (OD_600_) = 0.5 for 20 hours at 20°C. Cells are harvested by centrifugation and lysed by sonication (amplitude of 50%, 2-s on and 10-s off) for 3 min at 4°C after resuspension in cell (10 ml/g) in 20 mM tris-HCl (pH 8.0), 0.2 M NaCl, 5% glycerol, 5 mM 2-mercaptoethanol, and 5 mM imidazole (buffer A) supplemented with lysozyme (1 g/liter) and benzonase (5 U/ml). After centrifugation at 40,000*g*, the clarified lysate was loaded at 3 ml/min onto an immobilized nickel ion affinity chromatography column (5 ml; HisTrap Crude FF, GE Healthcare) and washed with buffer A with 25 mM imidazole. Elution was carried out at 2 ml/min with buffer A + 0.25 M imidazole with a linear gradient. The pool of purified protein was next dialyzed overnight against 20 mM tris-HCl (pH 8.0), 150 mM NaCl, 25 mM imidazole, and 5 mM 2-mercaptoethanol in the presence of TEV protease. His-tag–free recombinant protein was purified with a HisTrap Crude FF column. The flow-through was diluted five times in 20 mM tris-HCl (pH 8.0) and 1 mM dithiothreitol (DTT) (buffer B) and applied to an anion exchange chromatography column (5 ml; HiTrap Q FF, GE Healthcare). The recombinant protein was eluted with buffer B supplemented with 0.2 M NaCl. The purified samples were pooled and concentrated to 15 to 30 mg/ml with an Amicon centrifugal filtration device (Merck Millipore, 10 kDa) in 20 mM tris-HCl (pH 8.0), 0.2 M NaCl, 1 mM MnCl_2_, and 1 mM DTT. Protein samples for kinetic analysis were stored at −20°C in the same buffer supplemented with 55% glycerol.

#### Purification of GNAT7

OsGNAT7 and AtGNAT7 were purified as follows. Cell pellet (ca 25 g) that contains overexpressed proteins from BL21pLysS host bacteria was resuspended with 100 ml of lysis buffer A [20 mM tris (pH 8.0), 0.5 M NaCl, and 5 mM 2-mercaptoethanol], lysozyme (1 g/liter), and benzonase (5 U/ml). Lysis was performed with 90 s of sonication (power of 50% 10-s on/30-s off) at 4°C. Lysate was then centrifuged for 25 min at 40,000*g* at 4°C. The supernatant was applied to an Ni-IMAC (1 × 5 ml; HisTrap Crude, GE Healthcare). The cleared lysate was loaded at 3 ml/min into the HisTrap column equilibrated with buffer A. The column was washed with 23 column volumes (CVs) of buffer A until normal conductivity was reached. The protein of interest was eluted with a 20 CV gradient 0 to 100% buffer B [20 mM tris (pH 8.0), 0.2 M NaCl, 250 mM imidazole, and 5 mM 2-mercaptoethanol], followed by a 5 CV 100% step gradient. At 50% B, the gradient was stopped and replaced by a 100% B step gradient. TEV protease was added to the protein sample, incubated on ice in the cold-room for 2 hours, and then dialyzed overnight against 2 liters of 20 mM tris (pH 8.0), 0.2 M NaCl, 10 mM imidazole, and 5 mM 2-mercaptoethanol. His-tag–free GNAT7 was applied to an anion exchange chromatography column (5 ml; HiTrap Q FF, GE Healthcare), and the flow-though was collected. The sample was then centrifuged for 15 min at 40,000*g*, and the protein was stored at −80°C.

#### *Purification of Rubisco activase, Rca*β

The 260-kDa Rcaβ hexamer (subunit: 43.5 kDa) was purified as described below. The harvested overproducing cells were resuspended and incubated in buffer A [30 mM tris-HCl (pH 8.0), 0.15 M NaCl, 5 mM 2-mercaptoethanol, 5% glycerol, and 10 mM imidazole] containing lysozyme (1 g/liter) for 30 min with agitation before ultrasonication lysis for 3 min (2-s on, 10-s off model) using an Q700 sonicator (Qsonica, LLC). After centrifugation at 35,000*g* for 40 min at 4°C using an ultracentrifuge equipped with a JA-25.50 rotor (Beckman Coulter), the supernatant was passed through a 0.45-μm syringe filter and subsequently loaded onto a His trap FF 5 mL Ni-affinity column (Cytiva Life Sciences), washed with 8 CV buffer A, and eluted using a linear gradient of 10 to 250 mM imidazole in buffer B [30 mM tris-HCl (pH 8.0), 0.15 M NaCl, 5 mM 2-mercaptoethanol, 5% glycerol, and 0.25 M imidazole]. The protein of interest was pooled and then dialyzed overnight against 30 mM tris-HCl (pH 8.0), 0.15 M NaCl, and 1 mM DTT in the presence of TEV protease. His-tag–free proteins were further purified using a 5-ml His trap FF column. The pooled protein was diluted in buffer A applied to an anion-exchange chromatography column (5 ml; HiTrap Q FF, Cytiva Life Sciences) and eluted with buffer C [20 mM tris-HCl (pH 8.0), 1 mM DTT, and 0.2 M NaCl]. Protein fractions were pooled and concentrated using Amicon centrifugal filtration devices with a 30-kDa molecular weight cutoff (Merck Millipore). Hexameric Rca was further purified by gel filtration on a HiPrep 26/60 Sephacryl S-300 HR column (Cytiva) in buffer B [30 mM tris-HCl (pH 7.9), 0.15 M NaCl, 1 mM EDTA, and 1 mM DTT]. The purified Rca sample was pooled, concentrated, aliquoted in the same buffer plus 10% glycerol, flash-frozen in liquid N_2_, and stored at −80°C.

#### Purification of recombinant Rubisco

Rubisco was expressed in *E. coli* from pET28-AtRbcLSHis6, which features an RbcS3B gene version fused to a C-terminal His-tag and purified as described (*29*). Rubisco was purified in *E. coli* BL21 Star (DE3) cells (Invitrogen) transformed with plasmids pET11a-AtC60ab/C20, pDuet-AtR1/R2/RX/B2, pET28-AtRbcLSHis6, and the pET-Duet of interest expressing one, two, or three N-terminal processing enzymes (Fig. 2B, ^$^1-7). Bacteria were grown expressed in 2 liters of 2-YT medium to OD_600_ = 0.6 and with 0.5 mM IPTG for 18 hours at 23°C. The cell pellet was resuspended in extraction buffer [20 mM tris (pH 7.9), 0.2 M NaCl, 10 mM imidazole, lysozyme (1 g/liter), and 5% glycerol], disrupted by sonication, and centrifuged (30,000*g*, 30 min, 4°C; JA25.50 rotor, Beckman Coulter). The clarified lysate was then loaded on a nickel-nitrilotriacetic acid column (5 ml; HisTrap, GE Healthcare) equilibrated with 20 mM tris (pH 7.9), 0.2 M NaCl, 10 mM imidazole, and 5% glycerol and eluted with a linear imidazole gradient (10 to 250 mM). Fractions containing Rubisco were pooled and loaded on a HiPrep 16/60 Sephacryl S-200 HR column equilibrated with 20 mM tris-HCl (pH 8.0), 50 mM NaCl, and 1 mM DTT. Fractions containing Rubisco were then pooled and concentrated, and glycerol was added to a final concentration of 10%, flash-frozen in liquid nitrogen, and stored at −80°C. An example of denaturing and native electrophoretic quality control analysis of the purified complex is provided in **fig. S9** (A and B) (lane 1).

#### *Purification of Rubisco from* A. thaliana *plants*

Rubisco from *A. thaliana* WT and mutant lines was purified by adapting preexisting robust protocols (*29*, *64*). Plant seedlings were grown on soil in short days. Five grams of leaves (from two pots, each containing a single plant) were harvested at W7, briefly weighed, flash-frozen in liquid nitrogen, and stored at −80°C. All subsequent steps were performed consecutively in the cold room without interruptions. All buffers were degassed by vacuum filtration and sonication. Frozen leaves were rapidly transferred into 50 ml of Rubisco extraction buffer [50 mM tris (pH 7.9), 20 mM MgCl_2_, 20 mM NaHCO_3_, 0.2 mM EDTA, and 5% glycerol] supplemented with 1 mM phenylmethylsulfonyl fluoride, 5 mM DTT, and 2% (w/v) polyvinylpolypyrrolidone. Protease inhibitors were added either as one tablet of cOmplete protease inhibitor cocktail tablet EDTA free (Roche) per 50 ml of extract or, routinely, as 1% (v/v) of the protease inhibitor cocktail for plant tissue extracts (P9599, Sigma). Leaves were homogenized for 2 min in a precooled two-speed blender (Waring, HGB2WTG4) equipped with a stainless steel 110-ml mini blending container. Two cycles of 30-s high speed and 30-s low speed were applied. The homogenate was filtered first through four layers of cheesecloth (pore size/mesh opening, 250 μm; Vliwasoft non-woven pads) and next through two layers of miracloth (pore size of 25 μm; Merck). Each cloth was washed with 10 ml of the Rubisco extraction buffer. The filtrate was clarified by centrifugation at 30,000*g* for 30 min at 4°C. The supernatant (∼50 ml) was filtered through a 0.22-μm cellulose syringe filter before being applied to a self-cast Q-Sepharose FF column (25 ml; XK16x20 column, Cytiva) for anion exchange chromatography purification. After washing the column in buffer A with 3 CVs and then with 20% buffer B (Rubisco extraction buffer plus 5 mM DTT and 0.5 M NaCl, 3 CVs), elution was carried out with a 0.1 to 0.5 M linear NaCl gradient (20 to 100% buffer B, 8 CVs). Rubisco corresponds the major peak and eluted at ∼0.3 M NaCl as checked by SDS-PAGE. The pooled fractions (∼100 ml) were concentrated to ∼10 ml with a 100-kDa centrifugal filter (Millipore), loaded on a size exclusion chromatography column (precast HiPrep 26/60 Sephacryl S-300 HR, Cytiva), and eluted with buffer C [30 mM tris-HCl (pH 8.0), 0.15 M NaCl, 4 mM EDTA, and 1 mM DTT]. Fractions containing Rubisco (∼15 ml) were pooled and concentrated, after which glycerol was added to a final concentration of 10%. The final sample was aliquoted into Eppendorf tubes, flash-frozen in liquid nitrogen, and stored at −80°C.

### Enzyme activity assays

#### Measurements of AMPP2 activity by MALDI MS

A 200 μl of a mixture containing 50 mM tris-HCl (pH 7.4), 0.8 mM MnCl_2_, 1 mM DTT, 5 μM sodium cholate, and 250 to 500 μM synthetic peptide derived from RbcL N terminus was preincubated at 30°C. The cleavage reaction started with the addition of AMPP2 with different concentrations. Samples (10 μl) from the reaction were collected over time and further mixed with 90 μl of 10% (w/v) acetonitrile solution. The samples from previous step were then diluted five times in the matrix solution [α-cyano-4-hydroxycinnamic acid (5 mg/ml) solubilized in water/trifluoroacetic acid (TFA)/acetonitrile (50/50/0.1%)]. Each dilution (1 μl) was spotted on a metal target and dried. MS and MS/MS spectra of each sample were acquired with a 5800 MALDI-TOF-TOF (Sciex) instrument in positive ion mode. Survey scans were performed using delayed extraction (390 ns) in reflector mode for a total of 15,000 shots. MS/MS scans were operated with a collision energy of 1 kV. Peptide and fragment mass tolerances were set at 50 parts per million (ppm) and 0.8 Da, respectively. Mass spectra were analyzed with PeakView 2.2 software (Sciex, Macclesfield, UK). The default threshold in MS/MS peak labeling and finding was 5% (displayed with a black dot on the intensity axis), and the centroid height percentage was 50% as recommended by the constructor. MS/MS deviations from theoretical values were on average less than 0.03 Da. In MALDI, “adducts” refer to ions formed when the peptide analyte associates with other species, rather than just being simply protonated in positive-ion mode. The most common adducts are [M + H]^+^ (+1 Da), [M + Na]^+^ +22 Da, and [M + K]^+^ +38 Da and are visible with lesser intensities in the spectra displayed in Fig. 3B.

#### GNAT acetylation assay with Rubisco

For in vitro assays of Rubisco processing with GNAT7, 10 μM purified ΔS2_RbcL Rubisco complex purified from *E. coli* was incubated at 25°C for 60 min in the presence of 1 μM OsGNAT7, AtGNAT7, GNAT2, or AMPP2. Ac-CoA (100 μM) was added or not to the GNAT assays before MS analysis as the control.

#### Rubisco and Rca activity assays

The Rubisco coupled assay at 25°C with pyruvate kinase–lactate dehydrogenase was performed as previously described (*65*). A 2,3-bis bisphosphoglycerate–dependent phosphoglycerate mutase sample was purified as described previously (*66*), with specific activity of 220 U/ml. For activation to the “ECM” state, inactive Rubisco in the “E” state obtained following purification (5 μM) was incubated with 50 mM NaHCO_3_ and 25 mM MgCl_2_ for 15 min at 25°C. For inactivation to the “ER” state, Rubisco (5 μM active site) was incubated in ER buffer [20 mM tris-HCl (pH 8.0), 50 mM NaCl, and 4 mM EDTA] for 10 min before RuBP addition to a final concentration of 0.5 mM for 50 min at 25°C. The Rca activation assay was performed with Rubisco (0.5 μM active site) and Rca (2 μM protomer) using the PEPC-MDH coupled assay according to previously published procedures (*65*). The Rca-specific activity was determined as described previously (*67*). To assess the intrinsic ATPase activity of Rca, adenosine 5′-triphosphate (ATP) hydrolysis was enzymatically coupled to the oxidation of reduced nicotinamide adenine dinucleotide (NADH) and monitored spectrophotometrically (*68*).

### Electrospray ionization MS for peptide/protein characterization

Theoretical average molecular weights of RbcL isoforms were calculated using the ExPASy Compute pI/Mw tool (https://web.expasy.org/compute_pi/). All protein and peptide identifications were performed using the Araport11_pep_20220914 database (www.arabidopsis.org/download/overview).

#### High-resolution ESI-MS measurement of intact rubisco subunit masses

Mass measurement of intact Rubisco was performed on a Triple TOF 4600 Sciex (Macclesfield, UK) coupled to a nanoRSLC ultraperformance liquid chromatography system (Thermo Fisher Scientific) equipped with a C4-desalting column (5 μm, 300 A, 0.3 mm by 5 mm; Pepmap300, Thermo Fisher Scientific). Calibration was performed with a peptide mass standard kit (Sciex) containing a mixture of six peptides including bradykinin; angiotensin I, Glu1-fibropeptide B; and ACTH(1–17), ACTH(18–39), and ACTH(7–38) clips covering the 905.1 to 3660.2 Da mass/charge ratio (*m*/*z*) range. Prior infusion, 1 μl of the purified complex [i.e., total of 1.5 μg dissolved in 20 mM tris (pH 8.0), 50 mM NaCl, and 1 mM DTT] was desalted on a C4 column equilibrated in 0.1% formic acid (FA). After sample loading and desalting, proteins were eluted using a two-step gradient: step 1: 5 to 35% over 7 min and step 2: 35 to 95% over 2 min. Buffer B consisted of 0.1% FA in ACN, and buffer A consisted of 0.1% FA in water. A 1 μl of the protein elution was directly injected by infusion through an electrospray nebulization needle (voltage set to 3 kV and nebulizer N_2_ gas pressure ∼ 100 psi). For electrospray ionization (ESI) MS measurements, the instrument was operated in positive and radiofrequency quadrupole modes, with the time-of-flight (TOF) data being collected between *m*/*z* 400 and 2990 Da. The collision energy was set to 10 eV, and nitrogen was used as the collision gas. Analyst and Peakview software were used for acquisition and data processing, respectively. The spectral deconvolution of the protein sample (i.e., elution time of 4 to 9 min on the C4) was performed with the Bio Tool kit plug-in module of PeakView 2.2 (Sciex). Deconvolution parameters were as follows: mass ranges of 600 to 1800 Da *m*/*z* at the input and 10,000 to 60,000 Da with step mass of 1 Da at the output and 5000 or 1000 isotope resolution. The relevance of peak assignment of each species to all ions with different charges (40 to 80 in the case of RbcL) was checked, and the accurate mass was determined for the intact protein of each of the two subunits. The area of each assigned peak was extracted (see a spectrum example in fig. S3A). Peak with intensity levels lower than 50 was discarded, and the centroid percentage was 50%.

#### Proteome-wide N-terminal peptide profiling by N-terminomics

The SILProNAQ pipeline (*69*) was used for N-terminomics analysis with TPCK-trypsin (Sigma-Aldrich). The EnCOUNTER software (*70*) allowed quantification of the acetylation yield associated to each N terminus. A supplementary entry for RbcL, designated RbcL.2, was added to the protein database. This entry corresponds to an RbcL sequence lacking Ser^2^, i.e., starting with MP. This addition allowed the unusual RbcL N terminus to be considered as “likely” because EnCOUNTER negatively scores and discard the most unlikely N-terminal starts, including those at position 3, as observed in RbcL. Next, the occurrence of RbcL.2 enables the identification of previously missing PSMs starting at Pro^2^, as Met^1^ removal is treated as an N-terminal Met-loss modification. With this additional database entry, all possible N-termini resulting from RbcL processing could be identified with comparable confidence. The GAP pipeline was applied to bacterial samples as detailed in (*33*) to assess protein modifications of Rubisco in *E. coli*. To the *E. coli* proteome, we added the *A. thaliana* sequences of RbcL, RbcS3B, and all chaperone ORFs described in Fig. 2B. Analysis was focused on RbcL peptides. An LTQ Orbitrap Velos (Thermo Fisher Scientific) mass spectrometer was used to perform all N-terminomics–centered MS analysis with a MS survey scan acquired at 60,000 full width at half maximum (resolution using the lock-mass for internal calibration, for a *m*/*z* range from 400 to 2000 Da, followed by the fragmentation and MS/MS analysis of the 20 most intense precursor that are subject to collision-induced dissociation–MS with 20-s exclusion time for the acquired precursors.

#### Quantitative profiling of RbcL N-terminal peptide variants

Crude protein extracts (10 to 30 μg) from various *A. thaliana* lines and Rubisco-expressing *E. coli* variants were separated by SDS-PAGE and stained with Coomassie blue, and the RbcL bands (53 kDa; fig. S7A) were excised and sliced for shotgun MS analysis. The sample underwent cysteine block involving 10 mM DTT reduction at 56°C for 45 min followed by alkylation with 55 mM iodoacetamide in 200 μl for 1 hour in the dark at room temperature. Then, 200 μl of the freshly prepared diluted digestion enzyme was added into each tube. The tubes were kept at 4°C for about 1 hour to ensure proper absorption by the gel pieces and then placed at 37°C for about 17 hours (overnight digestion). Both TLCK-chymotrypsin and TPCK-trypsin (Sigma-Aldrich) were used for proteolysis. Chymotrypsin was generally preferred over trypsin because its initial cleavage at Phe^13^ produced an N-terminal peptide that is optimal for MS detection. Following centrifugation, peptides were recovered from the supernatant using 5% FA, 5% FA/acetonitrile mixture (40:60), and pure acetonitrile, respectively for 10 to 15 min. After vacuum drying and resolubilization in 40 μl of 2% ACN and 0.1% TFA, 3 of the 20 μl of a 10-fold dilution of the previous solution was applied to the LC-MS/MS analysis using the high-performance nanoflow liquid chromatography system (nanoElute 2, Bruker) coupled to timsTOF Pro 2 mass spectrometer (Bruker). Peptides were loaded on an Aurora Ultimate C18 UHPLC column (25 cm by 75 μm, 120-Å pore size, 1.7-μm particle size; ionopticks, Fitzroy, Australia) and eluted with a 0 to 30% gradient with solvent B for 30 min at 400 nl/min at 50°C. Solvent A was 0.1 % FA and 2% acetonitrile in water, and solvent B was 99.9% acetonitrile with 0.1% FA. MS and MS/MS spectra were recorded from *m*/*z* 100 to 1700 with a mobility scan range from 0.75 to 1.25 V·s/cm^2^. MS/MS spectra were acquired with the parallel accumulation serial fragmentation (PASEF) ion mobility–based acquisition mode using a number of PASEF MSMS scans set as 10. The “.mgf” files generated with the Data analysis software (Bruker) or “.d” files were used as input for the database search with either Mascot 2.7.0.1 (Matrix Science) or FragPipe 23.0 using MSFragger 4.2-rc18 (*71*) and IonQuant 1.11.5 (*72*). Cystein carbamidomethylation was set as fixed modification. Methionine oxidation and N-terminal protein and peptide acetylations were associated to the search as variable modifications. Lysine methylation and acetylation, lysine trimethylation, protein N-Met-loss, protein N-Met-loss plus acetylation, and N-terminal formylation were also used in some searches. Up to two missed cleavages were allowed in the searches. Peptide tolerance on parental ion was 15 ppm and 0.05 Da (20 to 40 ppm) on MS/MS (ESI-TRAP Instrument). Accepted peptide charges were set to 2+, 3+, and 4+. Automatic decoy search was selected to assess the false discovery rate associated to each search. We used semi-trypsin/P or semi-chymotrypsin as enzyme in the searches. When trypsin was used as cleaving enzyme the “Minimal Peptide Length In Search” parameter of Mascot was reduced from seven for chymotrypsin to six to take into account the PQTETK N-terminal peptide of RbcL. MSFragger was used as alternative search engine with identical parameters except the 5 to 35 residue peptide length window (99.7% of all cleavages). IonQuant was used in LFQ with MBR. Acetylation and any change affecting peptide hydrophobicity such as supplementary amino acids might cause significant impact on electrospray ionization efficiency and bias relative quantification (*73*). However, LFQ of the various N-terminal species of RbcL accumulating in crude and purified extracts tightly agreed with the data obtained with quantitative analysis of the full-length protein as accurately measured by intact MS (Fig. 1C). These data were also consistent with the SILProNAQ data assessing the acetylation yield on a given N-terminal. LFQ analysis resulting of bottom-up MS analysis was thus a suitable approach not inducing significant biases for assessing the relative accumulation of the different N-termini of RbcL in crude extracts. Raw data were processed with FragPipe. The MaxLFQ intensity (i.e., protein abundance estimate calculated from peptide-level MS data, normalized across samples) of each N-terminal isoform was used to quantify their relative abundance.

##### Proteome quantification via label-free approach

For LFQ, protein samples were digested with sequencing-grade trypsin (Promega). For W7 samples (Figs. 3C and 6C), 200 ng of a crude protein extract was applied to a timsTOF Pro 2. The samples were processed as described in the preceding section, except that a 90-min gradient was used for peptide separation. Data were processed using IonQuant 1.11.5 (*72*) as part of the FragPipe v23 package. W3 *gnat7* samples (fig. S5E) were processed as follows. Total extracts from each line (40 μg) were separated by SDS-PAGE, and the gels cut into four pieces. Each piece was hydrolyzed with trypsin, analyzed with an Orbitrap Velos as described in the previous section, and the data (“.raw” files) were processed with FragPipe. W3 *ampp2* samples (500 ng) in fig. S5D were analyzed using an EASY-nLC 1200 (Thermo Fisher Scientific) coupled to an Exploris 480 mass spectrometer (Thermo Fisher Scientific), and data were processed with MaxQuant 2.3.9 (https://maxquant.org/). Differentially abundant proteins of all samples were identified using a two-sample *t* test in Perseus v2.1.5.0 after data imputation (width = 0.3, down shift = 1.8) (*74*). At least two true values in each replicate were used to validate the data.

### Statistics and reproducibility

Each representative biochemical experiment displayed was repeated at least once with highly similar results. Most experiments were performed in tri- or quadruplicates (*N* = 3 or 4) as indicated in each figure. Mean values are displayed and error bars represent the standard error of the mean (SEM). Measurements were taken from distinct samples. Statistical analysis was performed using two-sided one-way analysis of variance (ANOVA) as figured in GraphPad Prism (v10.2.3). **** means *P* value <0.0001, *** means *P* value <0.001, ** means *P* value <0.01, and * means *P* value = 0.01.

## Supplementary Materials

### The PDF file includes:

Supplementary Text Figs. S1 to S11 Tables S1 to S4 Legends for data S1 to S3 References
