# Supplementary material for: Rubisco functional integrity requires a dedicated three-step maturation pathway
Source: Sci Adv. 2026 Sep 9;12(37):eaeh5060. doi: 10.1126/sciadv.aeh5060 (PMC13557074; doi:10.1126/sciadv.aeh5060)
Supplement: Supplementary file 1 [file sciadv.aeh5060_sm.pdf]

Science Advances
AAAS

Supplementary Materials for

Rubisco functional integrity requires a dedicated three-step maturation pathway

Dong Xie *et al.*

Corresponding author: Carmela Giglione, carmela.giglione@i2bc.paris-saclay.fr;
Thierry Meinnel, thierry.meinnel@i2bc.paris-saclay.fr

Sci. Adv. **12**, eaeh5060 (2026)
DOI: 10.1126/sciadv.aeh5060

The PDF file includes:

Supplementary Text
Figs. S1 to S11
Tables S1 to S4
Legends for data S1 to S3
References

Other Supplementary Material for this manuscript includes the following:

Data S1 to S3

Supplementary Text

AARE is active on dipeptides but not on RbcL

Using the knowledge accumulated from *A. thaliana*, we examined whether genes encoding enzymes with AARE activity could be identified. We found a single candidate (At4g14570, AARE), which encodes two predicted protein isoforms: a cytosolic form (UniProt, Q84LM4) and a second variant (GenBank, A0A2H1ZEM8) carrying an N-terminal transit peptide, suggesting plastid targeting. This open reading frame (ORF) is conserved in the plant lineage. Cleavage of the plastid-targeting sequence is predicted to lead to a polypeptide of similar size as the cytosolic isoform (84 kDa). To check whether AARE could display the *ac*-Ser_2 cleavage activity shown in Model 2 (**Fig. 1E**), we cloned the gene, expressed and purified the protein to homogeneity (**fig. S2A**). We confirmed that the purified enzyme displays AARE activity on a standard substrate (**fig. S2B**) (*76*). However, no AARE activity was observed with two peptide substrates, one mimicking RbcL (*ac*-Ser-21; *ac*-SPQTETKASVGFKAGVKEYKL) and another from F16P1 (*ac*-AVAADAAETKTAARKK), when using MALDI mass spectrometry even at high concentrations and for long incubation times (**fig. S2B**). Next, two independent *AARE* KO lines (*77*) were used to characterize the associated RbcL processing by using bottom-up MS. We did not observe change in RbcL processing in both *AARE KO* backgrounds compared to the WT line (**fig. S2C**), leading us to conclude that, unlike MetAP1C, AARE does not play a dominant role for the N-terminal maturation of RbcL.

AARE purification and assay

The full-length AARE ORF could be obtained after *de novo* total synthesis (Twist Biosciences, San Francisco) of a 2492 base fragment encompassing the 2469 bp of the longest open reading frame sequence (A0A2H1ZEM8). The DNA fragment inserted between the *Bam*HI and *Kpn*I restriction sites of pTwist vector. The human AARE (encoding UniProt entry P13798, NM_001640.3) ORF cloned in pCMV3-C-HA was obtained from Sino Biological (APEH). Both *Hs*AARE as a control and the synthetic *Arabidopsis thaliana* cDNA were inserted into pET15 or pET28 vectors for protein overexpression.

The *A. thaliana* ORF (764 amino acids, 84 kDa) and HsAARE were both inserted in pET15 to yield pET15-AtAARE and pET15-HsAARE featuring an N-terminal 6xHig tag followed by a TEV cleavage site. The protein was expressed in BL21Star(DE3) bacteria upon IPTG induction (0.5 mM) at OD_{600}=0.5 for 20 h at 20 °C. pET28- HsAARE and AtAARE were expressed in Rosetta(DE3) host. Cells are harvested by centrifugation and lysed by sonication (amplitude 50%, 2 s on and 10 s off) for 3 mins at 4 °C after resuspension in 10 mL/per g of cell in 20 mM Tris–HCl (pH 8.0), 0.2 M NaCl, 5% glycerol, 5 mM 2-mercaptoethanol, and 5 mM imidazole (buffer A) supplemented with 1 g/L lysozyme and 5 U/mL benzonase. After centrifugation at 40,000 g, the clarified lysate was loaded at 3 mL/min onto an immobilized nickel ion affinity chromatography column (HisTrap Crude FF, 5 mL, GE Healthcare) and washed with buffer A with 25 mM imidazole. Elution was carried out at 2 mL/min with buffer A plus 0.25 M imidazole with a linear gradient. The pool of purified protein was next dialyzed overnight against 20 mM Tris-HCl (pH 8.0), 150 mM NaCl, 25 mM imidazole, and 5 mM 2-mercaptoethanol in the presence of TEV protease. His-tag-free recombinant protein was purified with a HisTrap Crude FF column. The flow-through was diluted five times in 20 mM Tris-HCl (pH 8.0) and 1 mM dithiothreitol (DTT) (buffer B) and applied to an anion exchange chromatography column (HiTrap Q FF, 5 mL, GE Healthcare). The recombinant protein was eluted with buffer B supplemented with 0.2 M NaCl. The purified samples were pooled, concentrated to 15–30 mg/ mL with an Amicon centrifugal

filtration device (Merck Millipore, 10 kDa) in 20 mM Tris–HCl (pH 8.0), 0.2 M NaCl, and 1 mM DTT. Protein samples for kinetic analysis were stored at -20 °C in the same buffer supplemented with 55% glycerol. AARE activity was assessed either by MALDI-TOF MS using the same protocol as for AMPP2, or spectrophotometrically (*76*).

AMPP2 is the plastid homolog of *E. coli* PepP

Among the candidates with the highest score, we identified the mitochondrial intermediate cleaving peptidase Icp55 (At1g09300; 35% identity), an X-Pro dipeptidase-like/prolidase protein (At4g29500; 32% identity), a metalloprotease of unknown function (At3g05350, 25% identity), and MetAP1D (At4g37040; 21% identity), one of the three organellar MetAPs. All four proteins belong to the M24 metalloprotease family (*78*). The first two ranked enzymes are not plastid-localized, and MetAP1s, which preferentially cleave bulky N-terminal residues, do not fit the expected substrate specificity of an AMPP. Consistent with this, the *metap1d* mutant line did not show alteration of the RbcL N-terminal modification pattern (**Fig. 1B**). At3g05350 (AMPP2; Q8RY11) was found in the plastid proteome (*79*). The AMPP2 precursor contains a transit peptide, as assessed with TargetP 2.0 (https://services.healthtech.dtu.dk/services/TargetP-2.0/). Finally, N-terminally processed AMPP2 versions with the partially (60±3%) acetylated version starting at position 56 were observed in many of our MS surveys of leaf samples (https://n-terdb.i2bc.paris-saclay.fr/). The cleavage of AMPP2's chloroplast-targeting transit peptide (cTP), along with post-translational acetylation, provides additional evidence or its plastid localization. Furthermore, the AMPP2 sequence together with the predicted cTP is fully conserved in the plant-specific AMPP2 branch (**fig. S3B**).

RbcL is the unique substrate of AMPP2

Remarkably, aside from RbcL, only three proteins featured an N-terminal Met-X-Pro: NDHI (P56755, AtCg01090, Met_1-Leu_2-Pro_3), ATPH (P56760, AtCg00140, Met_1-Asn_2-Pro_3) and Cytochrome *f* (P56771, AtCg00540, Tyr_{35}-Pro_{36}-Ile_{37}, after removal of its N-terminal propeptide). However, our database (https://n-terdb.i2bc.paris-saclay.fr/) shows no evidence of Met_1 removal for NDHI or ATPH, consistent with general MetAP cleavage rules (*80*). Their native Met_1 therefore remains intact, rendering both proteins inaccessible to AMPP2. Cytochrome *f* could in principle be cleaved, but its luminal localization after translocation makes its neo-N-terminal Tyr_{35} inaccessible to stromal proteases-like AMPP2. Because AMPP2 also cleaves Met–Pro bonds (**Table 1**), we next considered plastid proteins starting with this motif. In the *A. thaliana* plastid proteome, three proteins display this feature: RR12 (P62126, AtCg00065, Met_1-Pro_2-Thr_3), YCF3 (P61843, AtCg00360, Met_1-Pro_2-Arg_3) and ClpPP1 (P56772, AtCg00670, Met_1-Pro_2-Ile_3. AMPP2 may be sensitive to these termini, but are also likely substrates for plastid MetAPs, suggesting overlapping rather than specific dependence on AMPP2. We also examined nuclear-encoded proteins imported into plastids. Only PsaE1 (Q9S831, At4g28750.1) contained a potential AMPP2-sensitive site (Asp_{48}-Pro_{49}). Cleavage prediction indicates a primary processing site at residues 45–46 of PsaE1 and several shorter variants extending to residue 53 (https://n-terdb.i2bc.paris-saclay.fr/), pointing to a stepwise trimming process that could involve AMPP2, although the exact mechanism remains unclear.

A

```
GREEN PLANTS
Streptophyta
 Angiosperms   Dicots        Vitis vinifera (Grape vine)         MSPQTETKASVGFKAGVKDYKLTYY
                             Arabidopsis thaliana                MSPQTETKASVGFKAGVKEYKLTYY
                             Brassica napus                      MSPQTETKASVGFKAGVKEYKLNYY
                             Opuntia quimilo (Cactus)            MSPQTETKASVGFKAGVKDYKLTYY
                             Spinacia oleracea (Spinach)         MSPQTETKASVEFKAGVKDYKLTYY
                             Lens culinaris                      MSPQTETKAKVGFQAGVKDYKLTYY
                             Pisum sativum                       MSPQTETKAKVGFKAGVKDYKLTYY
                             Nicotiana tabacum (Tobacco)         MSPQTETKASVGFKAGVKEYKLTYY
                             Solanum tuberosum (Potato)          MSPQTETKASVGFKAGVKEYKLTYY
                             Coffea arabica                      MSPQTETKASVGFKAGVKEYKLTYY
                             Populus trichocarpa                 MSPQTETKAGVGFKAGVKDYKLTYY
                             Magnolia macrophylla                MSPKTETKASVGFKAGVKEYKLTYY
                             Gossypium hirsutum (Cotton)         MSPQTETKASVGFKAGVKEYKLTYY
               Monocots      Musa acuminata (Banana)             MSPQTETKASVGFKAGVKDYKLNYY
                             Sorghum bicolor (Sorghum)           MSPQTETKASVGFKAGVKDYKLTYY
                             Saccharum officinarum (Sugarcan)    MSPQTETKASVGFKAGVKDYKLTYY
                             Zea mays (Corn)                     MSPQTETKASVGFKAGVKDYKLTYY
                             Cenchrus americanus (Pearlmill)     MSPQTETKASVGFKAGVKDYKLTYY
                             Hordeum vulgare (Barley)            MSPQTETKAGVGFQAGVKDYKLTYY
                             Triticum aestivum (Wheat)           MSPQTETKAGVGFKAGVKDYKLTYY
                             Oryza sativa (Rice)                 MSPQTETKASVGFKAGVKDYKLTYY
                             Glycine max (Soybean)               MSPQTETKASVGFKAGVKDYKLTYY
                             Manihot esculenta (Manioc)          MSPQTETKASVGFKAGVKDYKLTYY
               Magnoliids    Nymphaea colorata (Water lily)      MSPKTETKASVGFKAGVKDYRLTYY
   Ferns                     Azolla filiculoides                 MSPQTETKAGVGFKAGVRDYRLTYY
   Gymnosperms               Ginkgo biloba                       MSPQTETKASVGFKAGVKDYRLTYY
                             Pinus sylvestris                    MSPKTETKASVGFKAGVKDYRLTYY
   Bryophytes                Physcomitrium patens (Moss)         MSPRPEIKAGVGFKAGVKDYRLTYY
                             Anthoceros angustus(Hornwort)       MSPQTETKAGVGFKAGVKDYRLTYY
   Liverworts                Marchantia polymorpha               MSPQTETKAGVGFKAGVKDYRLTYY
Chlorophyta                  Chlamydomonas reinhardtii           MVPQTETKAGAGFKAGVKDYRLTYY
                             Ostreococcus tauri                  MAPQTETKTGTGFQAGVKDYRLTYY
EXCAVATA                     Euglena gracilis                    MSPQTETKTGAGFKAGVKDYRLTYY
```

B

```
             -58                      -25                        -1
Rice         aUCGAGUAGACCcUGUcGUUGUGAGAAUUCUUAAUUCAUGAGUUGUAGGGAGGGACgU AUG
Maize        aUCGAGUuGuCCcUGUgcUUGUGuGAAUUCUUAAUUCAUGAGUUGUAGGGAGGGACUU AUG
Cotton       aUCGAGUAGACCUUGUUGUUGcGAGAAUUCUUAAUUCAUGAGUUGUAGGGAGGGACUU AUG
Petunia      GUCGAGUAGACCUUGUUGUUGUGAGAAUUCUUAAUUCAUGAGUUGUAGGGAGGGAuUU AUG
Alfalfa      GUCGAGUAGACCUUGUUGUUGcuAGAAUUCUUAAUUCAUGAGUUGUAaGGgGGaAUUU AUG
Pea          GaguAGaccuugUUGUUGUUGcuAuAAUUCUUAAUUCAUGcGUUGUAGGGgGaGAuUU AUG
Spinach      GaguAGaccuugUUGcUuUguUGuaAAaauUaAAaUuugaAGUUGUAGGGAGGGACUU AUG
Barley       aUCGAGUAGACCcUGUUaUUGUGAGAAUUCUUAAUUCAaGAGUUGUAGGGAGGGACUU AUG
Tobacco      GUCGAGUAGACCUUGUUGUUGUGAGAAUUCUUAAUUCAUGAGUUGUAGGGAGGGAuUU AUG
Poplar       UUCGAGUAGACCUUGUUGCUGUGAGAAUUCUUAAUUCAUGAGUUGUAGGGAGGGGCUU AUG
Arabidopsis  GUCGAGUAGACCUUGUUGUUGcGAGAAUUCUUAAUUCAUGAGUUGUAGGGAGGGAuUU AUGUCACCACAAACAGAGACUAAAGCAAGUGU
                                                      m  s  c  r  e  g l/f M  S  P  Q  T  E  T  K  A  S  V

Consensus
Spinach      GAGUAGACCUUGUUGCUUUGUUGUAAAAAUUAAAAUUUGAAGUUGUAGGGAGGGACUU AUGUCACCACAAACAGAGACUAAAGCAAGUGU
                                                    AUG2          SD         AUG1
```

Fig. S1. Analysis of amino acid and nucleotide conservation at the initiation site of RbcL

(A) RbcL sequences from representative members of each subgroup of the plant phylum were selected. A Euglena sequence was also included, given that chloroplasts in the Excavata lineage are thought to derive from a symbiotic green alga. The sequences were aligned using Clustal X (*81*).

(B) The translation start site of RbcL is located on AUG[1]. The genome sequence around the putative starts codons from various RbcL sequences were aligned and the sequence was translated (below). AUG[1] is colored in green and AUG[2] is displayed in yellow. Stop codons are displayed in

red. SD means Shine-Dalgarno. The N-terminal Pro codon of mature plant RbcL is located at position 3 with respect with the preceding methionine codon (AUG^1). This strictly conserved methionine codon unambiguously corresponds to the translation start codon as it is preceded by an optimally-located Shine-Dalgarno (SD) sequence (consensus $GGAGG_{-6}$) that overlaps strong ribosome toeprint signals and which alteration inhibits RbcL translation (*82, 83*). Importantly, an in-frame Met codon (AUG^2) located 7-codon downstream is retrieved in most plant *RbcL* genes. Protein annotation in many plant species make it often appear as first codon leading to a short N-terminal (M_{-7}SCREGL) wrong extension with respect to the actual N-terminal Met (Met_1) (e.g., GenBank, MFQ6631301.1; UniProt, A0A811SNL5, A0A5P9NVU4). However, AUG^2 is not involved in RbcL mRNA translation as it (i) is not conserved and is modified for instance in an UGA stop codon in spinach or an AAG in barley and (ii), when occurring, is not preceded by any SD or ribosome binding toeprint features. Note that *rbcL* is the first cistron of a polycistronic transcription unit together with *accD* and *psaI*. Two transcription starts located -179 and -319 bases, respectively, downstream AUG^1 ensure downstream 30S ribosomal subunit binding around the SD (*84*, *85*)

A

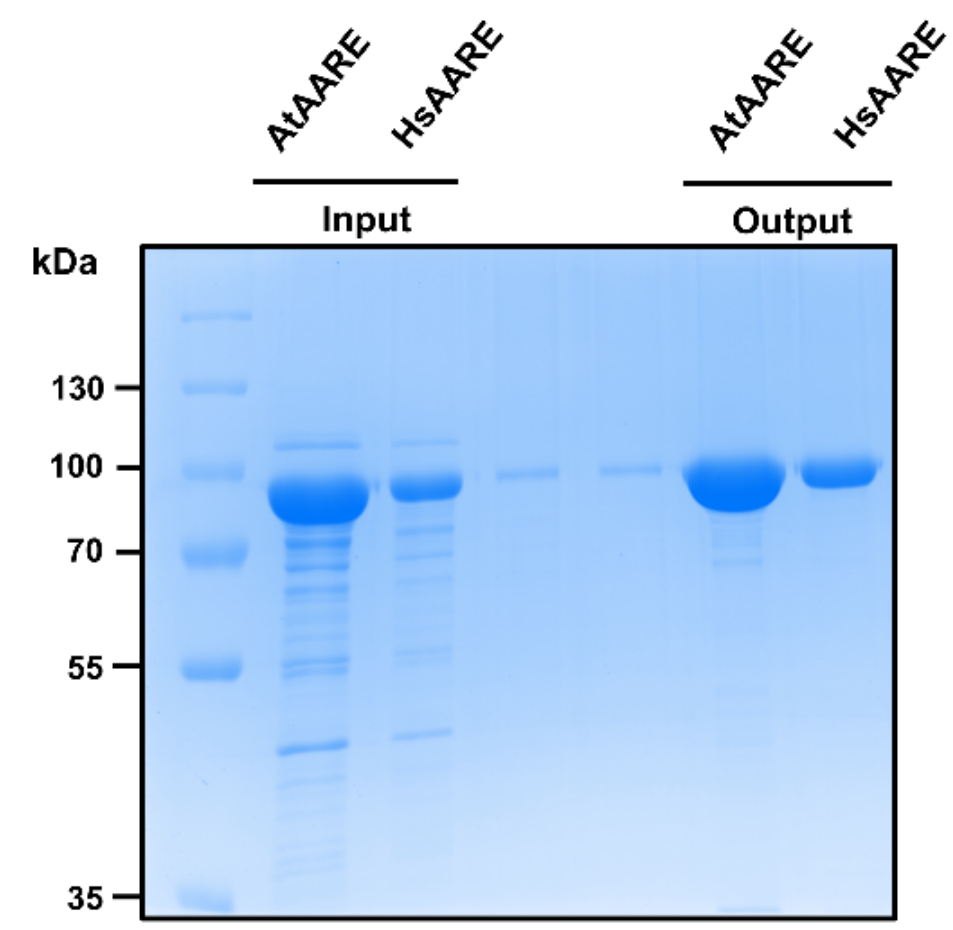

B

Substrate	k_{cat} (s^{-1})	K_m (mM)	Product detection
ac-Ala-*pNA*	16 ± 2	5 ± 2	Spectrophotometry
ac-SPQTETK	<< 0.1	<< 0.1	Mass spectrometry
ac-SPQTETKASVGFKAGVKEYKL	<< 0.1	<< 0.1	
ac-AVAADAAETKTAARKK	<< 0.1	<< 0.1	

C

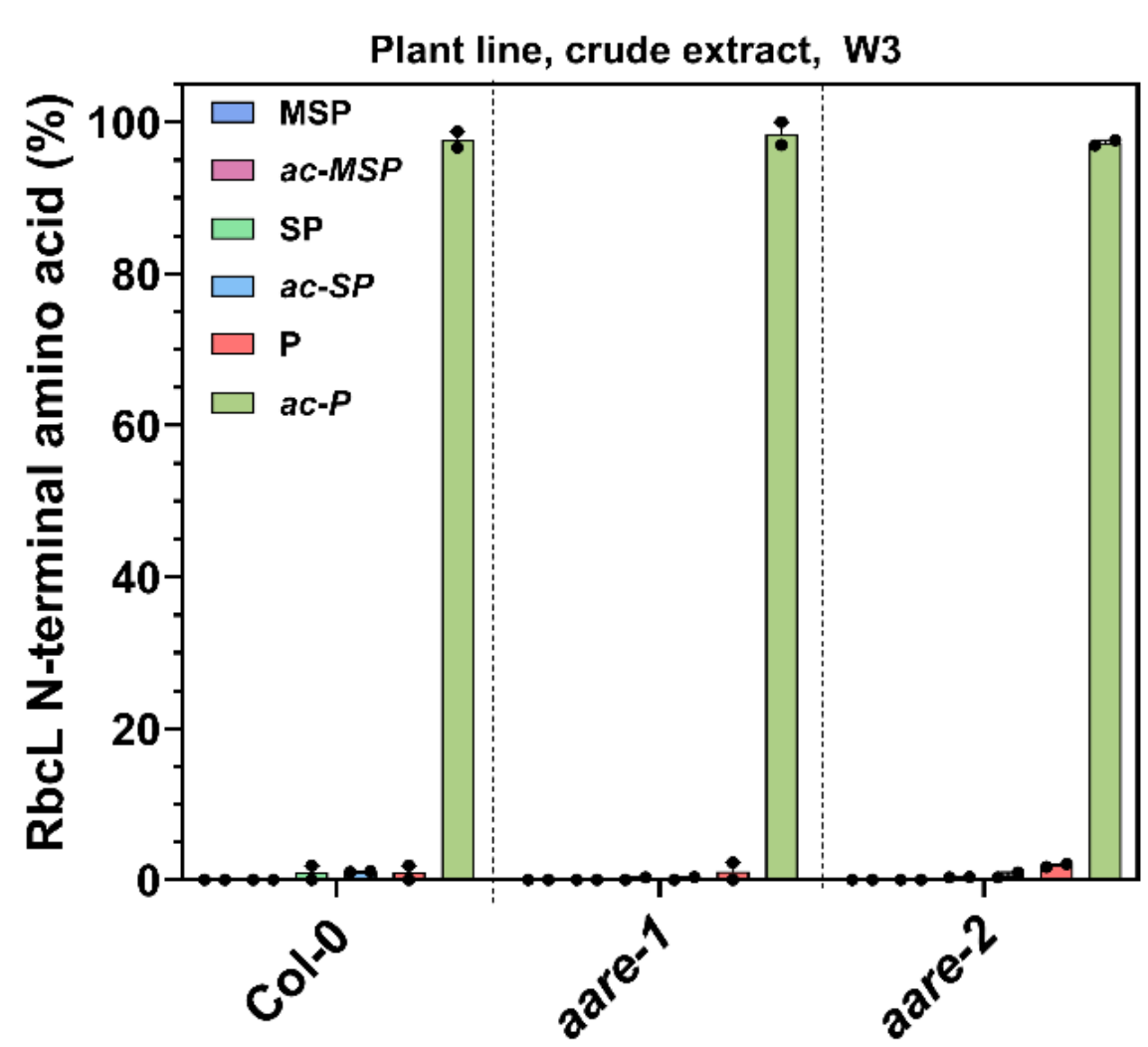

Fig. S2. AARE is active on dipeptides but not on RbcL

(A) Purified AARE from *A. thaliana* and humans. HsAARE was used as activity control. The AARE purification procedure is described in the **Supplementary Text**.

(B) Activity assay performed with a classic activity assay with modified dipeptide ac-Ala-pNA or by MALDI-Tof MS on longer peptides. pNA (para-nitroaniline) serves as a chromophore to monitor absorbance. The top peptide is derived from RbcL sequence (**Fig. 1A**). A table is displayed with the values when available.

(C) *aare* KO lines were used to derive the nature and intensities of the RbcL isoforms as in Fig. 1B.

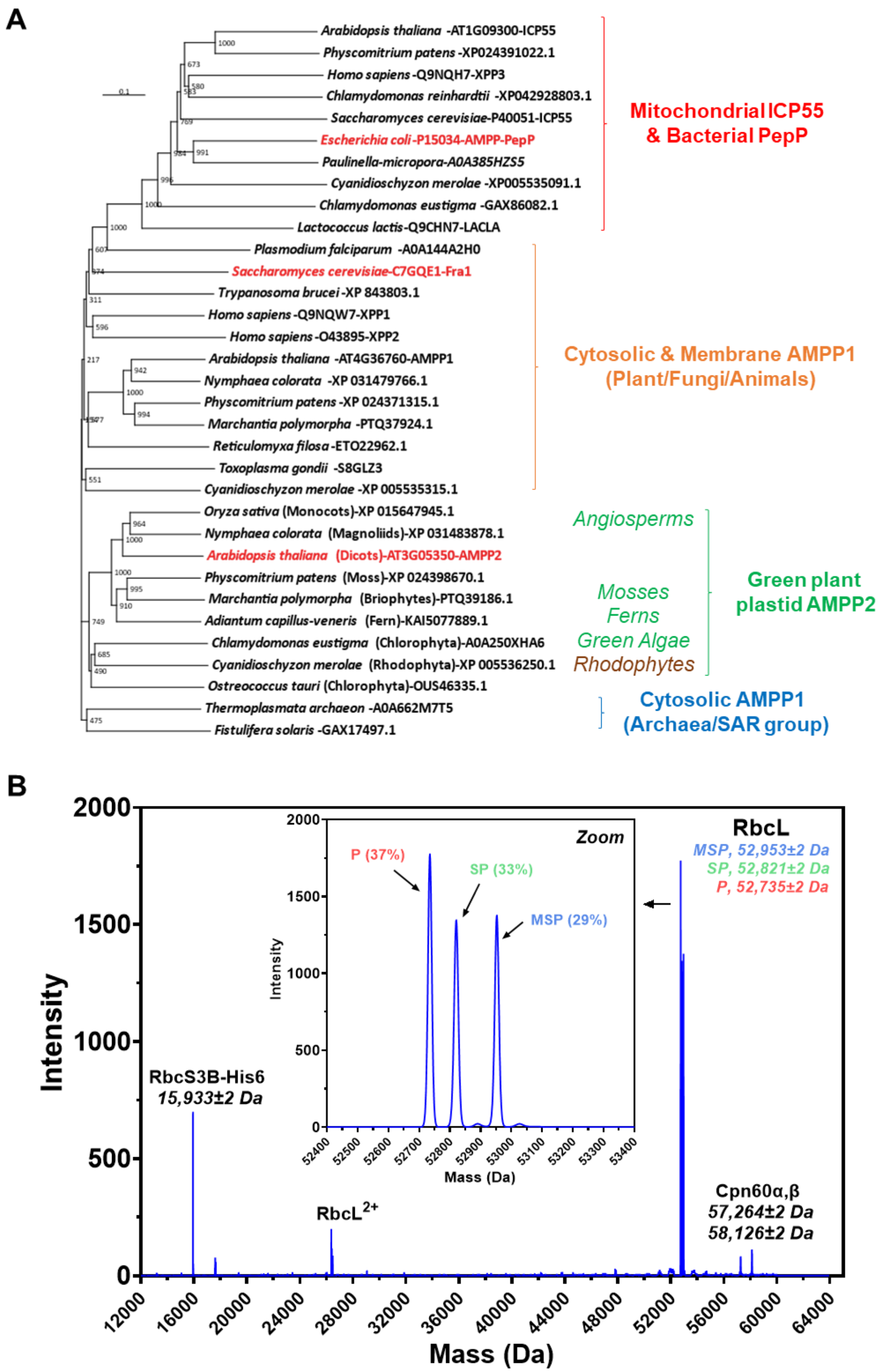
A
Arabidopsis thaliana -AT1G09300-ICP55
Physcomitrium patens -XP024391022.1
Homo sapiens -Q9NQH7-XPP3
Chlamydomonas reinhardtii -XP042928803.1
Saccharomyces cerevisiae-P40051-ICP55
Escherichia coli-P15034-AMPP-PepP
Paulinella-micropora-A0A385HZS5
Cyanidioschyzon merolae -XP005535091.1
Chlamydomonas eustigma -GAX86082.1
Lactococcus lactis-Q9CHN7-LACLA
Plasmodium falciparum -A0A144A2H0
Saccharomyces cerevisiae-C7GQE1-Fra1
Trypanosoma brucei -XP 843803.1
Homo sapiens -Q9NQW7-XPP1
Homo sapiens -O43895-XPP2
Arabidopsis thaliana -AT4G36760-AMPP1
Nymphaea colorata -XP 031479766.1
Physcomitrium patens -XP 024371315.1
Marchantia polymorpha -PTQ37924.1
Reticulomyxa filosa -ETO22962.1
Toxoplasma gondii -S8GLZ3
Cyanidioschyzon merolae -XP 005535315.1
Oryza sativa (Monocots)-XP 015647945.1
Nymphaea colorata (Magnoliids)-XP 031483878.1
Arabidopsis thaliana (Dicots)-AT3G05350-AMPP2
Physcomitrium patens (Moss)-XP 024398670.1
Marchantia polymorpha (Briophytes)-PTQ39186.1
Adiantum capillus-veneris (Fern)-KAI5077889.1
Chlamydomonas eustigma (Chlorophyta)-A0A250XHA6
Cyanidioschyzon merolae (Rhodophyta)-XP 005536250.1
Ostreococcus tauri (Chlorophyta)-OUS46335.1
Thermoplasmata archaeon -A0A662M7T5
Fistulifera solaris -GAX17497.1
Mitochondrial ICP55
& Bacterial PepP
Cytosolic & Membrane AMPP1
(Plant/Fungi/Animals)
Angiosperms
Mosses
Ferns
Green Algae
Rhodophytes
Green plant
plastid AMPP2
Cytosolic AMPP1
(Archaea/SAR group)
B
Intensity
Mass (Da)
RbcS3B-His6
15,933±2 Da
RbcL2+
RbcL
MSP, 52,953±2 Da
SP, 52,821±2 Da
P, 52,735±2 Da
Cpn60α,β
57,264±2 Da
58,126±2 Da
Zoom
P (37%)
SP (33%)
MSP (29%)

Fig. S3. AMPP2 is a distinctive branch in the AMPP family only retrieved in green plants

(A) Tree of AMPP lineage in the living organisms. A set of 33 sequences was selected to represent the breadth of AMPP sequence and organismal diversity. The sequences were aligned, and the bootstrap tree was constructed as previously described (*86*). Internal values labeled on each node record the stability of the branch over the 1,000 bootstrap replicates. Each UniProt entry, preceded by the species of origin, is shown at the end of each tree branch. The AMPP variants discussed in the text are highlighted in red.

(B) *E. coli*-purified Rubisco analysis by intact MS. The complete deconvoluted spectrum is displayed showing all major peaks including some minor contaminants originating from Cpn60 at higher masses. AMPP-catalyzed cleavage of the SP isoform (Ser_2) by *Ec*PepP promotes the *in cellulo* formation of the P isoform (Pro_3).

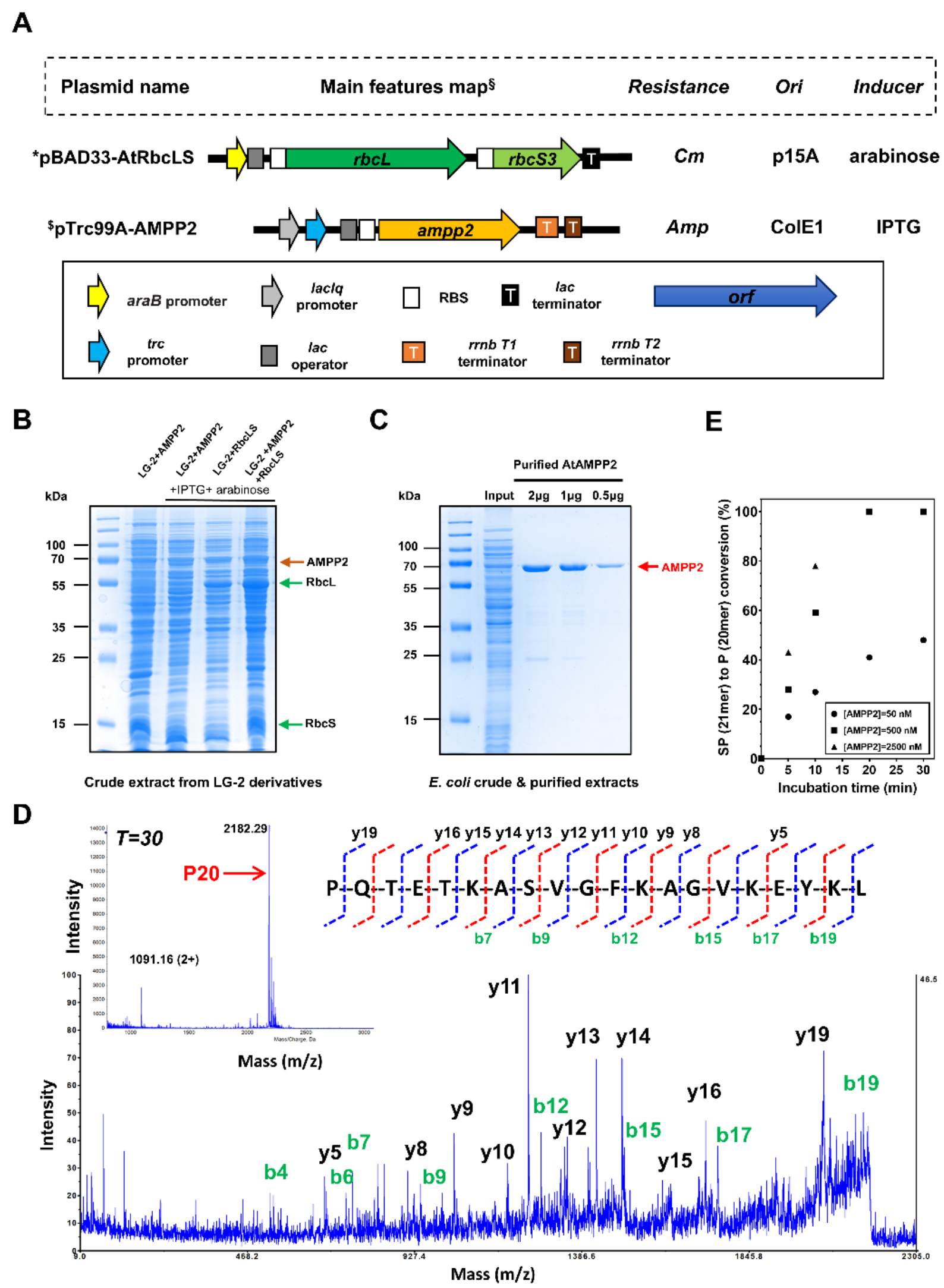

Fig. S4. AMPP2 cleaves Ser2 from RbcL in *E. coli* and from short peptides in vitro

(A) Rubisco transgenic expression system in *pepP* LG-2 *E. coli*. Plasmids map used for expression of AMPP2 and both Rubisco subunits in strain LG-2. pBAD33RbcsL is from (*28*). pTrc99A-AMPP2 is from this work. AMPP2 and RbcS3B ORFs are devoid of their plastid targeting signal. RbcL has no such sequence because the gene is plastid-encoded. The main features associated to each plasmid are indicated.

(B) Crude extracts (10 µg) expressing the different constructs corresponding to the protein samples were analyzed by SDS-PAGE.

(C) SDS-PAGE analysis of 0.5-2 µg of purified AMPP2 from *E. coli*. A Coomassie-staining is displayed. Input is the soluble fraction before loading into the column for purification purposes.

(D) MS2 spectrum the product of AMPP2 cleavage from the 21-mer SP-starting peptide derived from RbcL shown in **Fig. 3B** (T=20).

(E) Quantitative analysis of the kinetics of cleavage of the above RbcL-derived peptide upon incubation with various AMPP2 amounts. The relative intensity of the MALDI peaks (20-mer/21-mer) at each time and enzyme concentration was used to calculate the conversion percentage displayed in the figure.

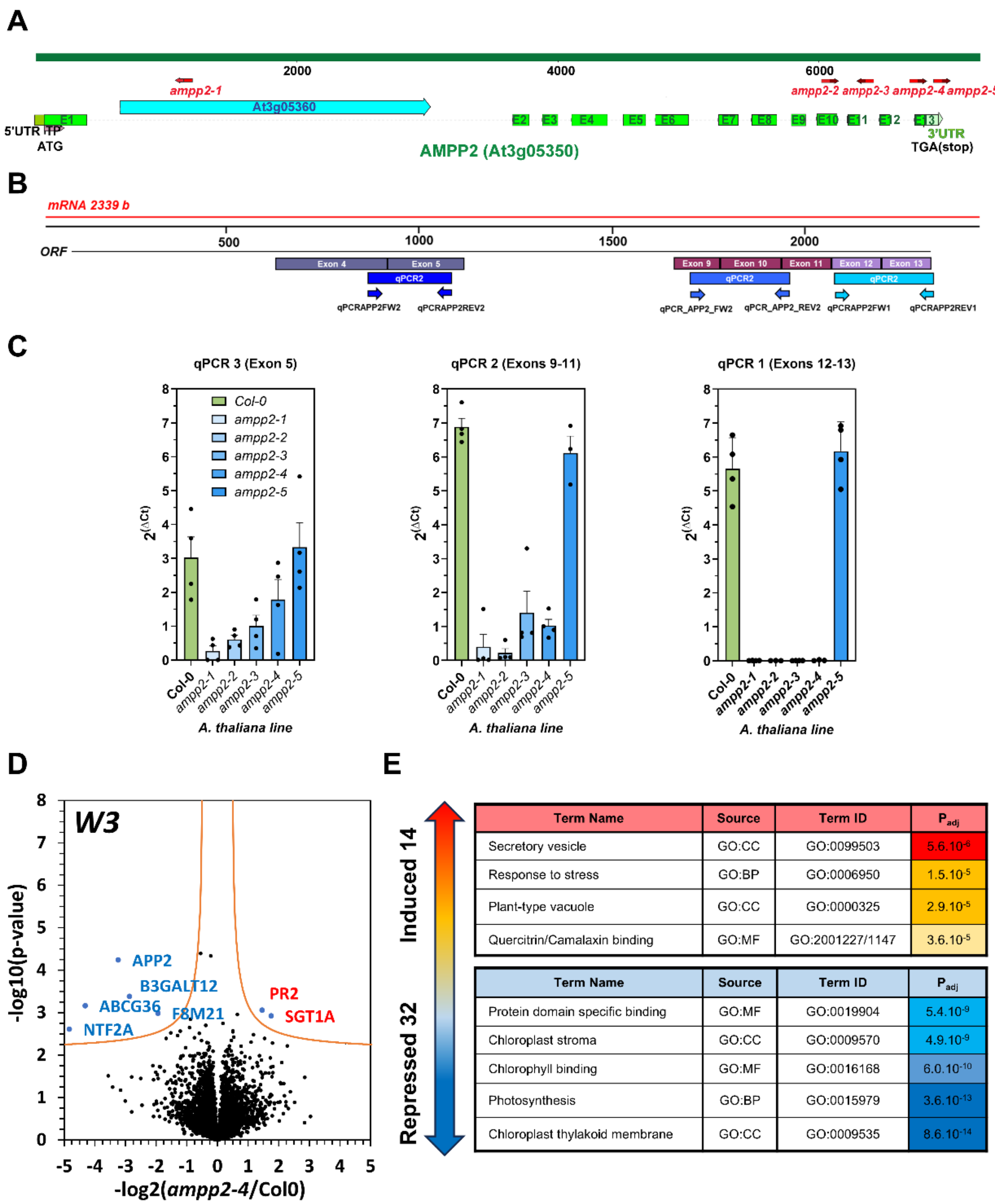

Fig. S5. AMPP2 T-DNA insertion lines and impact of AMPP2 and GNAT7 inactivation at W3

(A) We amplified the construct with the left primer for AMPP2 and the LB primer for the SALK T-DNA. The amplicon was next sequenced with the LB primer. LB insertions were identified counter-clockwise at positions G1136 (*ampp2-1*), T4865 (*ampp2-6*), T6358 (*ampp2-3*), and clockwise at positions C6068 (*ampp2-2*), A6764 (*ampp2-4*) and T6947 (*ampp2-5*) in the map shown in panel A. The left border is indicated in deep purple and its orientation is indicated with an arrow for each line. The red box corresponds to the AMPP2 gene border which was identified. TP is for targeting peptide.

(B) The pair of primers used for qPCR and their localization on the mRNA are indicated.

(C) qPCR analysis of the relative expression of the mRNA transcript of the above ampp2 T-DNA insertion lines and of the parental WT lines by comparing mRNA expression with to two different housekeeping genes (PEX and SAND). The PCR amplification products for qPCR-R1/2/3 pairs is displayed. The 2(Δct) values associated to qPCR of ampp2:1-5 insertion lines were compared to those of the WT and displayed on each of the corresponding panels (n=4).
(D) Label Free Quantification proteomics analysis of line *ampp2-4* was performed on 3-week-old (W3) leaves. Samples were analyzed using an EASY-nLC 1200 coupled to an Exploris 480 mass spectrometer. Data were processed with MaxLFQ and Perseus 2.0.3.0. The significance curve set is displayed in orange. The down- and up-regulated proteins are highlighted in red and green, respectively.
(E) Gene Ontology (GO) analysis (*87*) of proteins regulated upon AMPP2 inactivation at W7. GO analysis was performed at https://biit.cs.ut.ee/gprofiler/gost with the whole set of up- (top, green squared, 14 entries) or down-regulated (bottom, red squared, 32 entries) proteins identified in the *ampp2-4* background compared to WT seedlings (Data S2). The protein categories are classified according to their probability of occurrence. The p-adj threshold values were $<10^{-5}$ for induced proteins, 10^{-8} for repressed ones**.** A color gradient from light to dark represents GO functions, with darker colors indicating lower p-adj values and thus higher significance

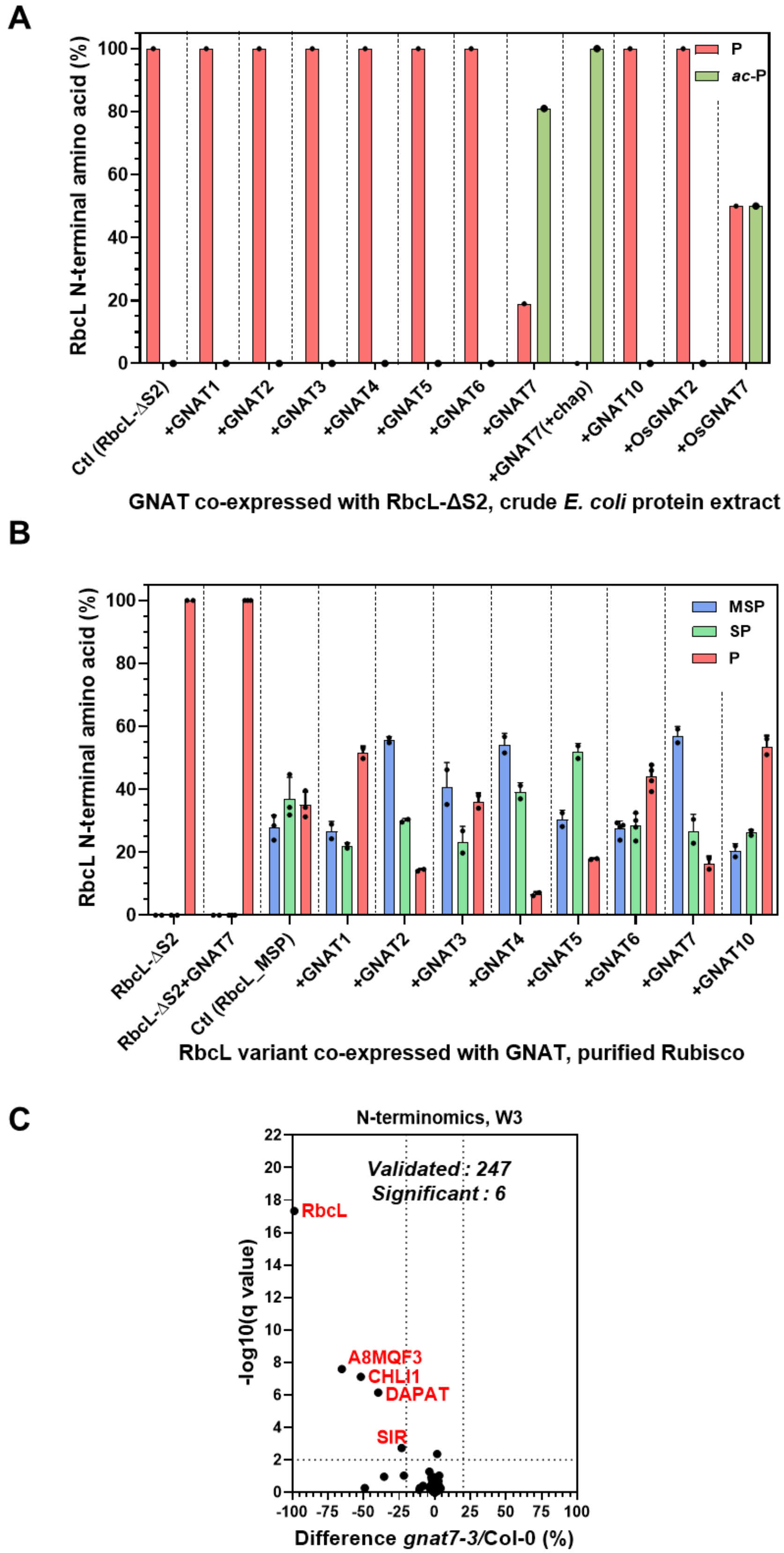
A
RbcL N-terminal amino acid (%)
P
ac-P
Ctl (RbcL-ΔS2)
+GNAT1
+GNAT2
+GNAT3
+GNAT4
+GNAT5
+GNAT6
+GNAT7
+GNAT7(+chap)
+GNAT10
+OsGNAT2
+OsGNAT7
GNAT co-expressed with RbcL-ΔS2, crude E. coli protein extract
B
RbcL N-terminal amino acid (%)
MSP
SP
P
RbcL-ΔS2
RbcL-ΔS2+GNAT7
Ctl (RbcL_MSP)
+GNAT1
+GNAT2
+GNAT3
+GNAT4
+GNAT5
+GNAT6
+GNAT7
+GNAT10
RbcL variant co-expressed with GNAT, purified Rubisco
C
N-terminomics, W3
Validated : 247
Significant : 6
RbcL
A8MQF3
CHLI1
DAPAT
SIR
-log10(q value)
Difference gnat7-3/Col-0 (%)

Fig. S6. Impact of GNAT on RbcL processing.

(A) Screening of GNATs involved in RbcL N-terminal Pro acetylation; the ΔS2 variant was expressed in *E. coli* BL 21 cells without chaperones (except when indicated) to promote, unlike the MSP WT variant, an N-terminal Pro as unique N-terminus and minimize further biased effects due to the exposure of an intermediate isoform as described later on in the manuscript. Each of the eight plastid GNATs from *A. thaliana* (AtGNAT1-10) and the rice homolog of GNAT2 and GNAT7 (OsGNAT2/7) were expressed from their respective pACYC-Duet derivative (**Fig. 2B**). The RbcL band of the crude extract (soluble and insoluble fractions) was cut and digested with chymotrypsin. Relative abundances are displayed as in **Fig. 1B**.
(B) Same as **Fig. 4B** but focusing on the amino acids accumulating at the N-terminus independently of their acetylation status each isoform for which a value of 100 was given (for relative quantification of each N-terminus see panel B). Rubisco was purified from the extract arising from the bacterial Rubisco complete expression system described in **Fig. 2B**. An example of native and denaturing electrophoretic quality control analysis of the purified complex is provided in **fig. S7A,D** (lane 1). Mass spectrometry of the intact protein was performed and quantified (see a spectrum example in **Fig. 1C** and **fig. S3A**); the relative amount of each deduced isoform is displayed. This panel focused on the acetylation yield N-terminal amino acid.
(C) SILProNAQ analysis of *gnat7-3*; the analysis is identical to that for *gnat7-1* displayed in **Fig. 4C**.

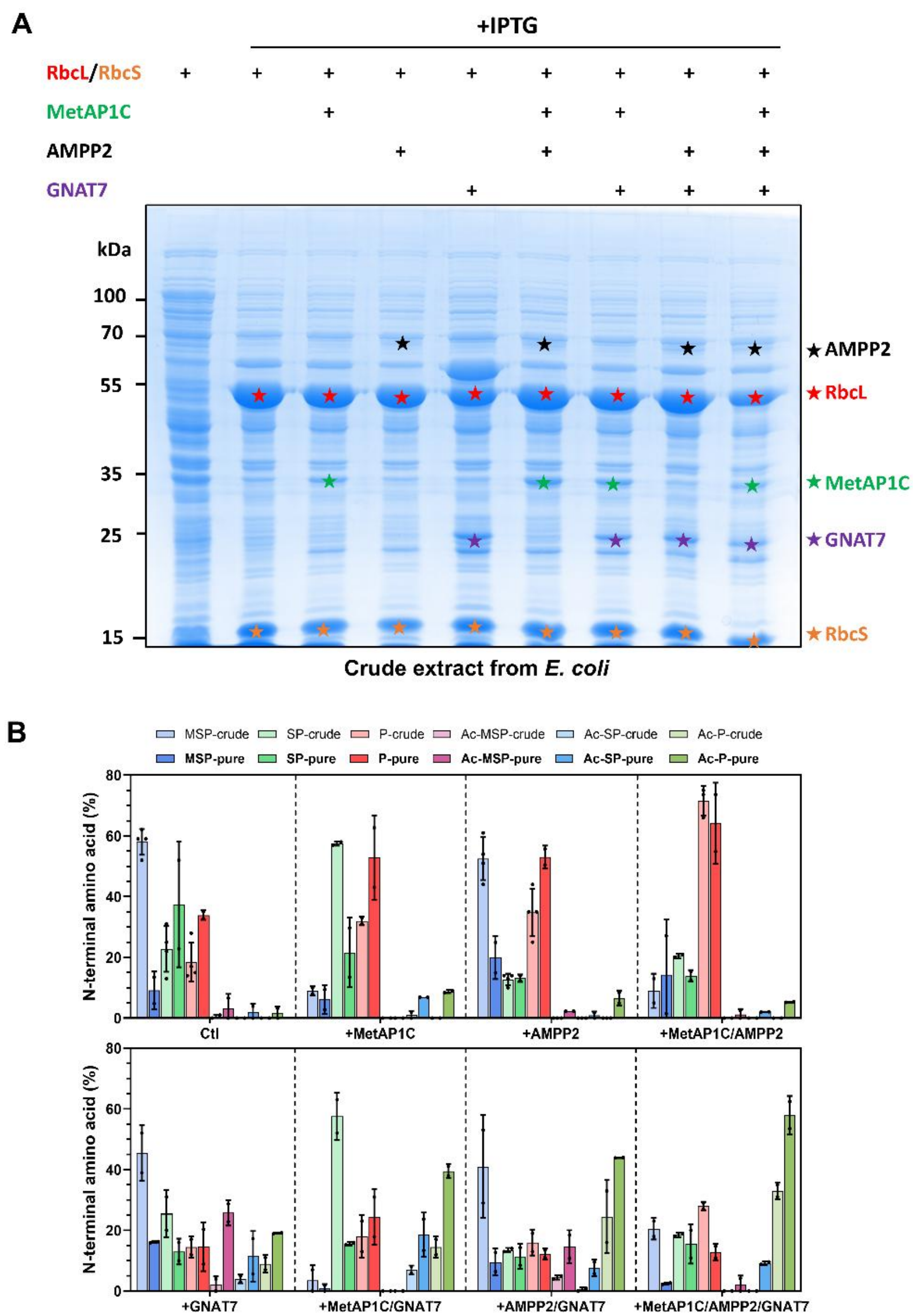

Fig. S7. Preferred accumulation of properly processed RbcL in the holoenzyme

(A) The different modifiers were expressed from BL21Star cells expressing the relevant chaperone and Rubisco plasmids together a combination of one, two or three of the AMPP2, MetAP1C or GNAT7 modifiers (**Fig. 2B**). Crude bacterial protein extracts (10 µg) were checked for each overexpressing enzyme using the proteins purified to homogeneity as a control. A Coomassie-blue staining is displayed to distinguish the various overexpressed enzymes (see colored label on the right).

(B) Quantitative comparison of the nature of the N-termini accumulating in RbcL in both crude extract and purified protein samples. Quantification was derived identically after chymotrypsin cleavage of the RbcL band in either sample as in **Fig. 5E,F**. The impact of processing obtained upon co-expression of the three modifiers is compared under with the six combinations displayed.

A

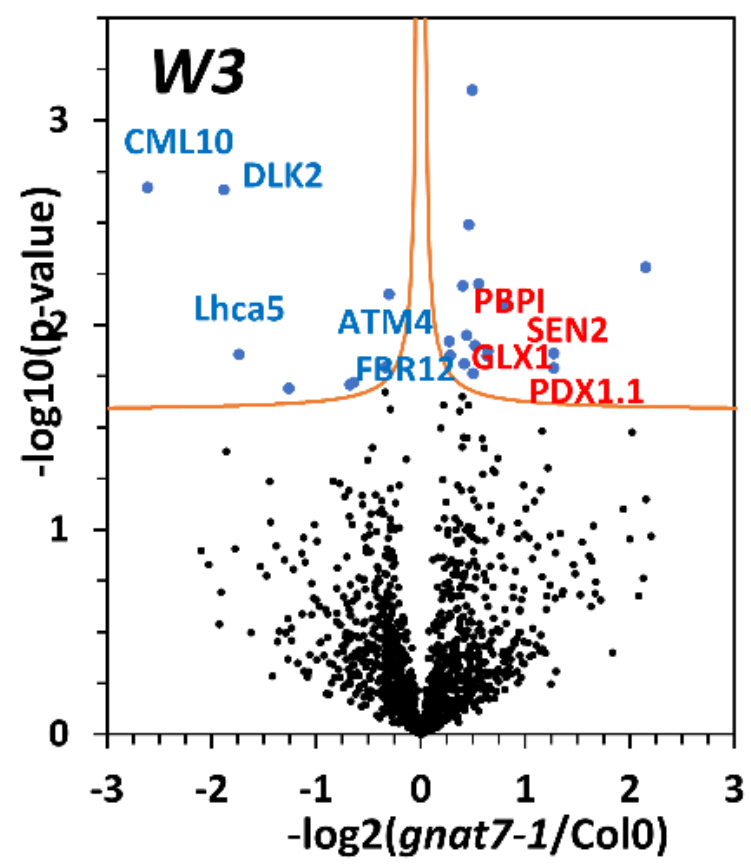

B

Induced 86

Term Name	Source	Term ID	P_{adj}
Cytosol	GO:CC	GO:0005829	$2.6.10^{-20}$
Response to stress	GO:BP	GO:0006950	$3.0.10^{-14}$
Plant-type vacuole	GO:CC	GO:0000325	$8.5.10^{-11}$
Protein folding	GO:BP	GO:0006457	$2.0.10^{-10}$
Cell-cell junction	GO:CC	GO:0005911	$7.9.10^{-9}$
Protein folding chaperone	GO:MF	GO:0044183	$2.3.10^{-8}$

Repressed 129

Term Name	Source	Term ID	P_{adj}
Tetrapyrrole biosynthesis process	GO:BP	GO:0033014	$3.2.10^{-9}$
Apoplast	GO:CC	GO:0048046	$2.9.10^{-9}$
mRNA binding	GO:MF	GO:0003729	$1.0.10^{-22}$
Protein domain specific binding	GO:MF	GO:0019904	$1.5.10^{-24}$
Chlorophyll binding	GO:MF	GO:0016168	$2.0.10^{-28}$
Photosynthesis	GO:BP	GO:0015979	$9.1.10^{-59}$
Chloroplast stroma	GO:CC	GO:0009570	$2.4.10^{-61}$
Plastid envelope	GO:CC	GO:0009526	$7.6.10^{-90}$

Fig. S8. Impact of GNAT7 inactivation in W3 and W7 *Arabidopsis* leaves

(A) Label Free Quantification proteomics analysis of line *gnat7-1* was performed on 3-week-old (W3) leaves. Samples were analyzed using an EASY-nLC 1200 coupled to an Exploris 480 mass spectrometer. Data were processed with MaxLFQ and Perseus 2.0.3.0. The significance curve set is displayed in orange. The down- and up-regulated proteins are highlighted in red and green, respectively. MS analysis performed on an Orbitrap-Velos and processed with FragPipe and Perseus.

(B) Gene Ontology (GO) analysis (*87*) of proteins regulated upon GNAT7 inactivation at W7. GO analysis was performed at https://biit.cs.ut.ee/gprofiler/gost with the whole set of up- or down-regulated proteins identified in the *gnat7-1* background compared to WT seedlings (panel D, Data S2). The protein categories are classified according to their probability of occurrence. The threshold values was p-adj<10^{-8}. A color gradient from light to dark represents GO functions, with darker colors indicating lower p-adj values and thus higher significance.

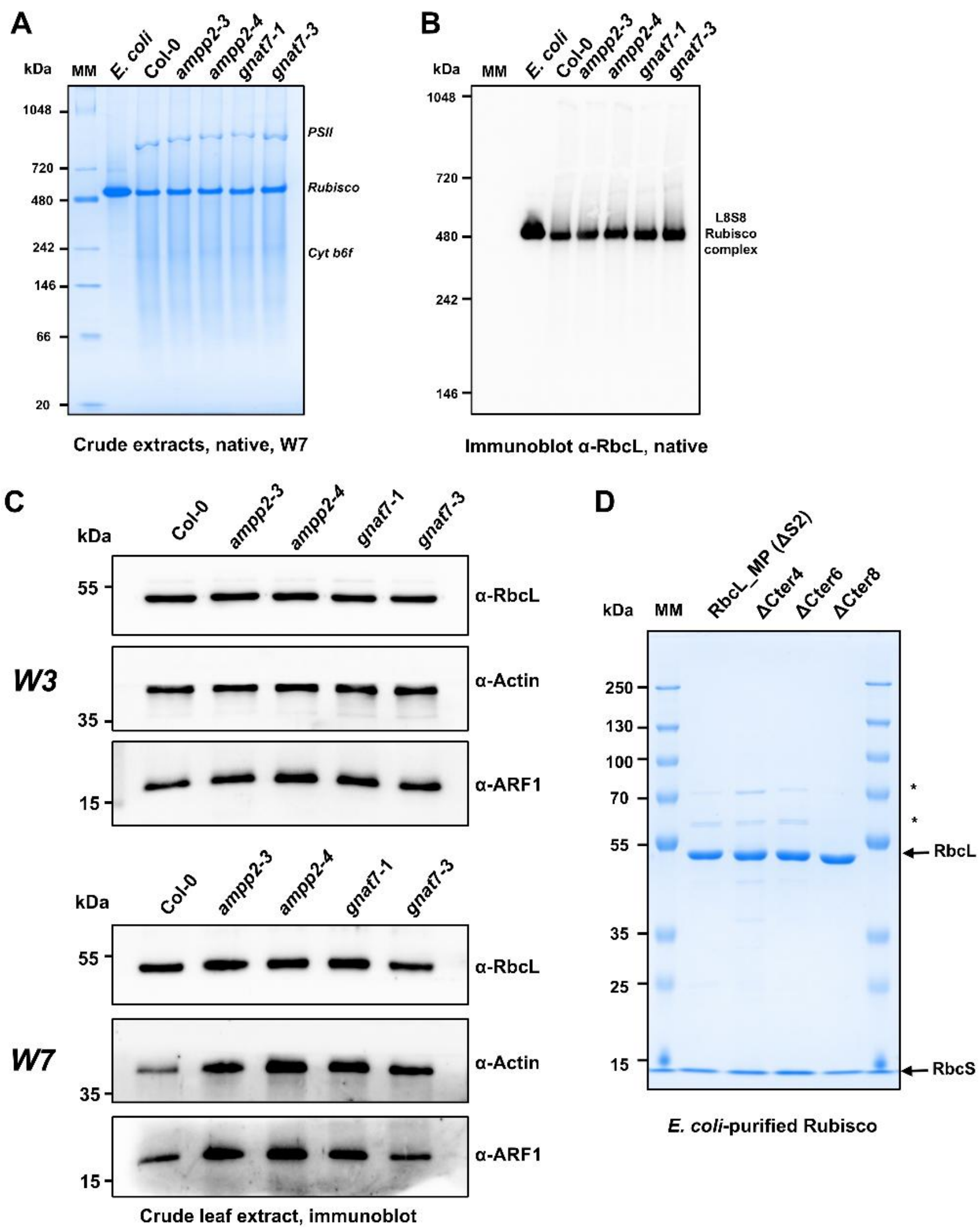

Fig. S9. Analysis of RbcL upon altercations of its N- and C-terminus

Assembly and N-terminal processing analysis of Rubisco from various genetic backgrounds.

(A) Native gel analysis from various plant lines stained with bromophenol blue. A crude plant extract was loaded for each line. Purified Rubisco from *E. coli* cells was used as a control. 2 µg was loaded on each lane. The identity major complexes which can be distinguished are displayed on the right. The gel shows that Rubisco is by far the major plant complex. MM, molecular weight marker.

(B) Immunoblot of the gel in panel A with RbcL antibodies to evidence smaller unassembled complexes.

(C) Immunoblotting of crude plants extracts at W3 (Top) and W7 (Bottom) of various KO mutant lines of *GNAT7* and *AMPP2* compared to WT. The complete time-course quantification picture with replicates of RbcL accumulation normalized with α-Arf1 or α-actin antibodies is displayed in **Fig. 7A**.

(D) PAGE analysis of 1 µg of Rubisco from RbcL C-terminal truncated versions (MP start context, ΔCter4/6/8) produced in *E. coli*. A Coomassie-Blue staining is displayed. The vertical arrow indicates RbcL; the star symbol indicates minor chaperone contaminants.

A

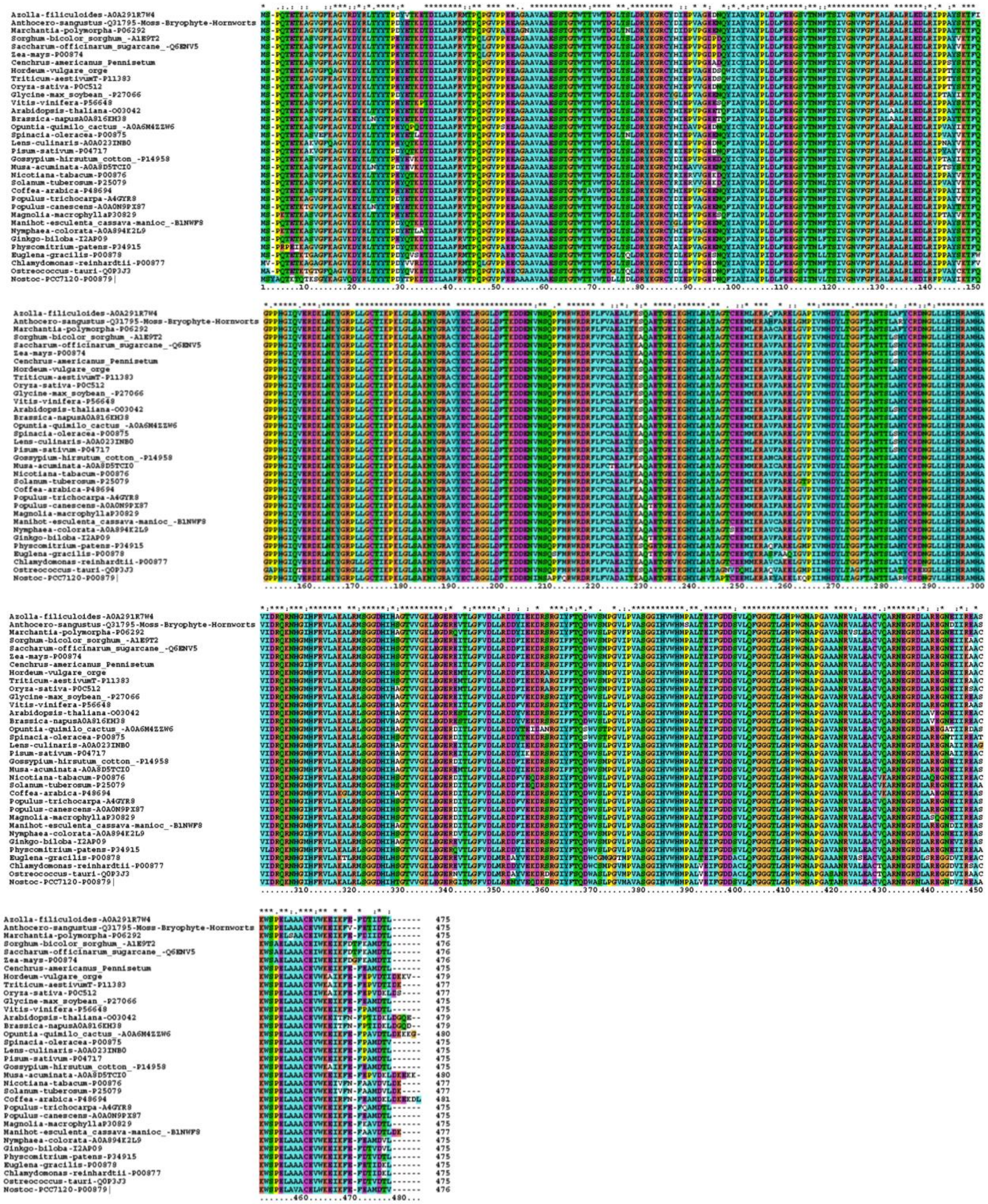
Azolla-filiculoides-A0A291R7W4
Anthocero-sangustus-Q31795-Moss-Bryophyte-Hornworts
Marchantia-polymorpha-P06292
Sorghum-bicolor_sorghum_-A1E9T2
Saccharum-officinarum_sugarcane_-Q6ENV5
Zea-mays-P00874
Cenchrus-americanus_Pennisetum
Hordeum-vulgare_orge
Triticum-aestivumT-P11383
Oryza-sativa-P0C512
Glycine-max_soybean_-P27066
Vitis-vinifera-P56648
Arabidopsis-thaliana-O03042
Brassica-napusA0A816KH38
Opuntia-quimilo_cactus_-A0A6M4ZZW6
Spinacia-oleracea-P00875
Lens-culinaris-A0A023INB0
Pisum-sativum-P04717
Gossypium-hirsutum_cotton_-P14958
Musa-acuminata-A0A8D5TCI0
Nicotiana-tabacum-P00876
Solanum-tuberosum-P25079
Coffea-arabica-P48694
Populus-trichocarpa-A4GYR8
Populus-canescens-A0A0N9PX87
Magnolia-macrophyllaP30829
Manihot-esculenta_cassava-manioc_-B1NWF8
Nymphaea-colorata-A0A894E2L9
Ginkgo-biloba-I2AP09
Physcomitrium-patens-P34915
Euglena-gracilis-P00878
Chlamydomonas-reinhardtii-P00877
Ostreococcus-tauri-Q0P3J3
Nostoc-PCC7120-P00879

B

Length	PDB Id	3D structure	Species	Sequence (XXX=missing in 3D)	
477	4RUB	A9-D473	Tobacco	NtRbcL-P00876-Nicotiana-tabacu	WSPELAAACEVWKEIVFN-FAAVDVLDK
477			Potato	StRbcL-P25079-Solanum-tuberosum	WSPELAAACEVWKEIVFN-FAAMDVLDK
481			Coffee	CaRbcL-P48694-Coffea-arabica	WSPELAAACEVWKEIRFN-FEAMDKLDKEKDL
475			Bigleaf ficus	MmRbcL-P30829-Magnolia-macroph	WSPELAAACEVWKEIKFE-FKAVDTL
475			White Poplar	PtRbcL-Q14FE9-Populus-alba	WSPELAAACEVWKEIKFE-FQAMDTL
475			Eucalyptus	Eg RbcL-Q49KZ0-Eucalyptus-globulus	WSPELAAACEVWKEIKFE-FEAMDTL
479	5IU0	F13-L475	Arabidopsis	AtRbcL-O03042-Arabidopsis-thaliana	WSPELAAACEVWKEITFN-FPTIDKLDGQE
475	4HHH	G12-F469	Pea	PsRbcL-P04717-Pisum-sativum	WSPELAAACEVWKEIKFE-FPAMDTL
475			Grape vine	Vv-P56648-Vitis-vinifera	WSPELAAACEVWKEIKFE-FPAMDTL
475	1IR1	E12-V475	Spinach	SoRbcL-P00875-Spinacia-olerace	WSPELAAACEVWKEIKFE-FPAMDTV
	1UPP	A9-V475		SoRbcL-P00875-Spinacia-olerace	WSPELAAACEVWKEIKFE-FPAMDTV
475			Water lily	NcRbcL-A0A894K2L9-Nymphaea-col	WSPELAAACEVWKEIKFE-FEAMDVL
480*			Gentian	A0A8F2XPN3-Gentiana-leucomelaena	WSPELAAACEVWKEIRFN-FKAVDTLDPLKS
480*			Banana	MaRbcL-A0A8D5TCI0-Musa-acumina	WSPELAAACEVWKEIKFE-FEPVDKLDKEKK
477			Rice	OsRbcL-P0C512-Oryza-sativa-	WSPELAAACEIWKAIKFE-FEPVDKLDS
477			Wheat	TaRbcL-P11383-Triticum-aestivum	WSPELAAACEVWKAIKFE-FEPVDTIDK
476			Maize	ZmRbcL-P00874-Zea-mays	WSAELAAACEIWKEIKFDGFKAMDTI
476			Nostoc	NsRBcL-P00879-Nostoc	WSPELAVACELWKEIKFE-FEAMDTV

* wrong N-terminal annotation (7 N-terminal residues more, not taken into account)

Fig. S10. The last residues of the C-terminus of RbcL can be extended up to 6 residues in some isoforms of the plant phylum.

(A) RbcL sequences from representative members of each subgroup of the plant phylum were aligned with Clustal X (*81*). Conserved residues were colored.

(B) The C-terminal residues of some RbcL sequence are depicted (last column). The length of the RbcL amino acid sequence is reported. When available, crystal structure PDB IDs of some representative members are indicated in the second column. The coordinates of the structured regions in the density (third column), with starting and last residues.

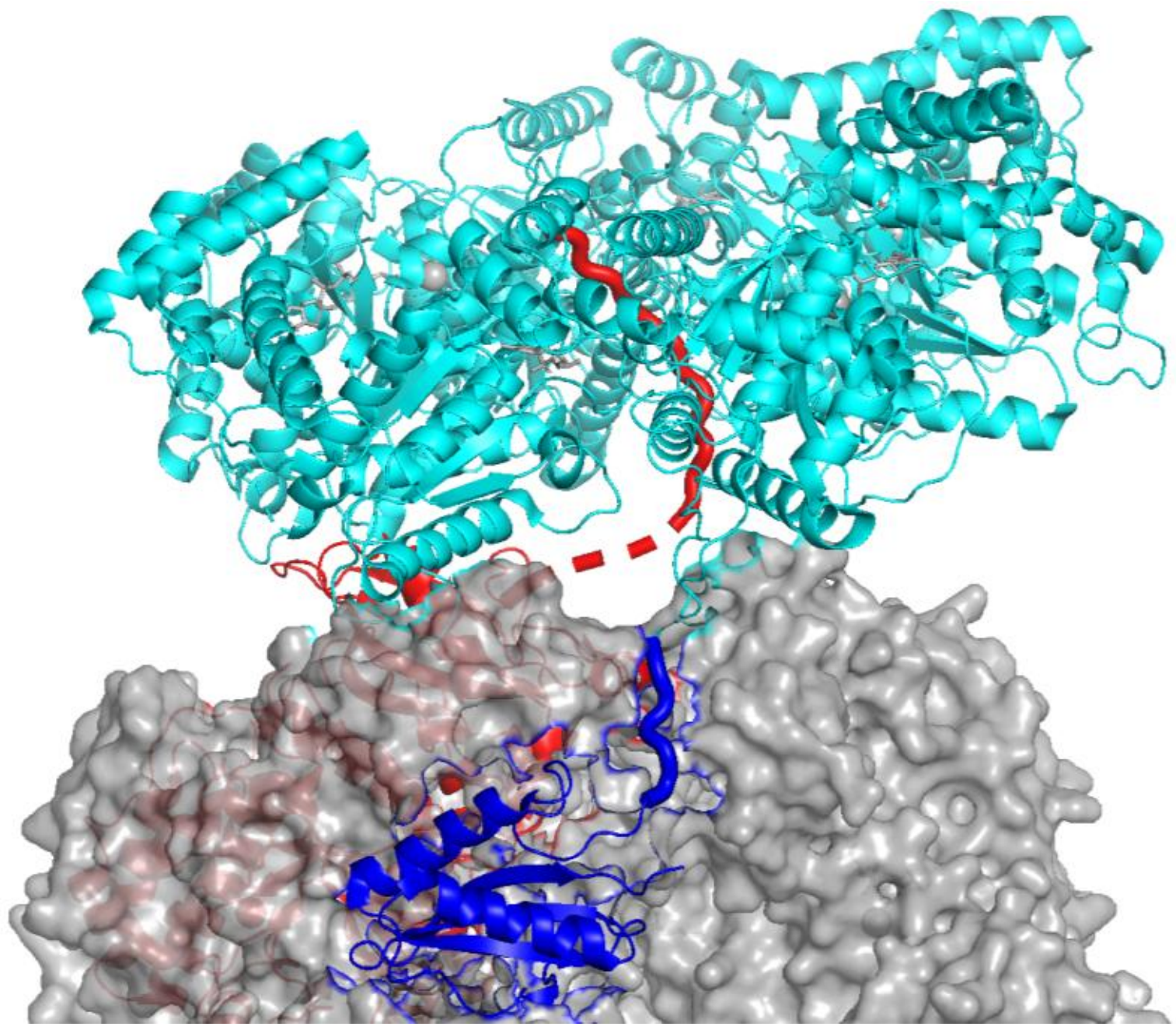

Fig. S11. The N-terminus of RbcL makes major interaction regions with Rca.

Location of the N-terminus of RbcL at the interface between Rca subunits and Rubisco complexes. Rca is displayed in turquoise, RbcL in grey and red and RbcS in dark blue. The N-terminus of RbcL L-D subunit is displayed in red as a ribbon.

**Origins**	**Background**	**Sequence features**	k_{cat} (s^{-1})	$K_m^{(RuBP)}$ (µM)[a]	$k_{cat}/K_m^{(RuBP)}$ $(M^{-1}.s^{-1})$	**Relative** $k_{cat}/K_m^{(RuBP)}$
A. thaliana	WT	*ac*-Pro3	4.21±0.06	35.4±1.7	118,859±6047	100.0
	gnat7-1	Pro3	4.32±0.05	36.1±1.5	119,833±5047	100.8
	ampp2-4	*ac*-Ser2	4.21±0.06	74.5±4.6	56,502±3580	47.5
	ampp2-gnat7	Ser2	4.34±0.07	70.5±4.2	61,552±3824	51.8
E. coli	ΔS2	Pro3	3.53±0.06	35.4±2.2	99,746±6347	83.9
	ΔS2+GNAT7	*ac*-Pro3	3.55±0.06	36.6±2.1	96,942±5694	81.6
	ΔS2+ΔC4	Pro3+ΔC4	3.31±0.09	41.5±3.9	79,759±7747	67.1
	ΔS2+ΔC6	Pro3+ΔC6	3.43±0.07	43.6±3.0	78,742±5691	66.2
	ΔS2+ C8	Pro3+ΔC8	3.27±0.09	37.3±3.7	87,715±8989	73.8

[a] The K_m value for RuBP (K_m^{RuBP}) was measured at saturating $MgCl_2$ and $NaHCO_3$ concentrations

Table S1. Catalytic parameters of purified Rubisco from various origins

Designation	Source/Reference
pUNI-AMPP2	ABRC
pET15a-His6-TEV-AMPP2_73-710	This study
pETM30-AMPP2	This study
pTrc99a-AMPP2	This study
pCMV3-HsAARE-C-HA	Sino Biological Inc.
pTwist-AtAARE	Twist Bioscience
pET15a-HsAARE	This study
pET15a-AtAARE	This study
pBAD33-AtRbcLS (RbcL+Rbc3B)	Gift
pET28b-AtRbcLS-His6	Gift
pET11a-AtCpn60α/β-AtCpn20	Gift
pETDuet-AtRaf1/Raf2/RbcX/BSD2	Gift
pTH24-TEV protease	Lab stock
pACYCDuet-AtGNAT1	This study
pACYCDuet-AtGNAT2	This study
pACYCDuet-AtGNAT3	This study
pACYCDuet-AtGNAT4	This study
pACYCDuet-AtGNAT5	This study
pACYCDuet-AtGNAT6	This study
pACYCDuet-AtGNAT7	This study
pACYCDuet-AtGNAT10	This study
pETM41-MBP-AtGNAT7_54-267	Lab stock
pETM41-MBP-AtGNAT7_37-274	Lab stock
pETM41-MBP-OsGNAT7_67-291	This study
pET28-AtGNAT7_37-274	This study
pET28-OsGNAT7_67-291	This study
pACYCDuet-AtMetAP1C	This study
pACYCDuet-AMPP2	This study
pACYCDuet-AtMetAP1C-AMPP2	This study
pACYCDuet-AtMetAP1C-AtGNAT7	This study
pACYCDuet-AMPP2-AtGNAT7	This study
pACYCDuet-AtMetAP1C-AMPP2-AtGNAT7	This study
pET28b-AtRbcL_ΔS2-S (MPQ-RbcL)	This study
pET28b-AtRbcL_S2A-S (MAPQ-RbcL)	This study
pET28b-AtRbcL_P3A-S (MSAQ-RbcL)	This study
pET28b-AtRbcL_ΔS2_P3A-S (MAQ-RbcL)	This study
pET28b-AtRbcL_ΔS2_ΔC4-S (RbcL lack 4 aa at C-ter)	This study
pET28b-AtRbcL_ΔS2_ΔC6-S (RbcL lack 6 aa at C-ter)	This study
pET28b-AtRbcL_ΔS2_ΔC8-S (RbcL lack 8 aa at C-ter)	This study
pUNI51-AtRcaα	ABRC
pET15a-His6-AtRcaα_59-474	This study
pET15a-His6-AtRcaβ	This study
pET15b-Strep-dPGM	Gift

Table S2. Plasmids and constructs

Name	Sequence 5’ to 3’
LG-2-test-pepPKO-F	CGGCGAAACCGGTGAAGCTATCGA
LG-2-test-pepPKO-R	GTGGTGATGGCAGTTGCGCAATCC
AMPP2topET15-F	CAGAGAAAACTTGTATTTCCAGGGCCATATGGCTCAAACCAAAGTAGTTGTCG
AtAMPPtopET15-R	GGCTTTGTTAGCAGCCGGATCCTTATGGTTTGGCTAAGGGTCTTG
pTrc99A_F	AAGCTTGGCTGTTTTGGCGG
pTrc99A_R	CATGGTCTGTTTCCTGTGTGAAATTGTTATC
AMPP2toTrc99A_F	CACACAGGAAACAGACCATGGCTCAAACCAAAGTAGTTGTC
AMPP2toTrc99A_R	CCGCCAAAACAGCCAAGCTTTTATGGTTTGGCTAAGGGTCTTGT
AtAAREtopET15-F	TGTATTTCCAGGGCCATATGGATTCTTCTGGAACTGATTCG
AtAAREtopET15-R	CTTTGTTAGCAGCCGGATCCTTACAGCTTGCAGTACTTGTTGAA
AtAAREtopET28-F	TAAGAAGGAGATATACCATGGATTCTTCTGGAACTGATT
AtAAREtopET28-R	CAGTGGTGGTGGTGGTGGTGCAGCTTGCAGTACTTGTTGA
pET15-F	AGGATCCGGCTGCTAACAAAG
pET15-R	CATATGGCCCTGGAAATACAAGTTTTC
pet-28-F	CACCACCACCACCACCACTGA
pet-28-R	CATGGTATATCTCCTTCTTAAAGTTAAACAAAATTATTTCTAGAGG
pet-s-F	TACGACTCACTATAGGGGAATTGT
pet-s-R	TTCAGCAAAAAACCCCTCAAG
AtGNAT2-F	GCGCCGAGCTCGAATTCGGATCCCTACTTTGGGTACCAAAACATGCCTTTG
AtGNAT2-R	AACTTTAATAAGGAGATATACCATGGCGAGTAGAAAGTTGAAGACTTTGC
AtGNAT3-Nter-R	GTCTTTCTTCAACGTTGCCA
AtGNAT3toDuet1-F2	CTTTAATAAGGAGATATACCATGGCAAGTGATGTTATTGTATCTG
AtGNAT3-rev-F	AGTGATGTTATTGTATCTGTGTTTT
AtGNAT3-rev-R	GCCATGGTATATCTCCTTATTAAAG
AtGNAT4-F	AACTTTAATAAGGAGATATACCATGGACGCAAGTCAAATAGTTGATC
AtGNAT4-R	GCCGAGCTCGAATTCGGATCCTTACCGAAACTGTTCAAGA
AtGNAT5-F	AACTTTAATAAGGAGATATACCATGGACACAGCTACAGAAACTGGAG
AtGNAT5-R	GCGCCGAGCTCGAATTCGGATCCTTACACATTTGCAGAGGAGGTCAGC
AtGNAT6-F	ATATACCATGGCGAAGAAGGAAGAACTCTCTGTAC
AtGNAT6-R	AGCTCGAATTCTTAGCTTGTGTACTGGAGC
AtGNAT7-F	GAGAATCTTTATTTTCAGGGCGCCATGGCGTTCCGTCCCGTCGCTGCTT
AtGNAT7-R	CGACGGAGCTCGAATTCGGATCCTTACATGGACATGATTGGGGAGGTA
OsGNAT2-F	GTTTAACTTTAATAAGGAGATATACCATGGCGCCGATCGAGGAAGAGGAACCGCTG
OsGNAT2-R	CCGAGCTCGAATTCGGATCCTTAAAAACGCGGATACCAGAACATGCCC
OsGNAT7-F	GAGAATCTTTATTTTCAGGGCGCCATGGCGCTGGTGCTGGAGGGTA
OsGNAT7-R	CGACGGAGCTCGAATTCGGATCCTTACAGCGCGTCTTTCTTCA
MetAP1BtoDuet2-F	GTATAAGAAGGAGATATACATATGGGGTTAGAGGAAGCCATTAGAATC
MetAP1BtoDuet2-R	GCGGTTTCTTTACCAGACTCGAGTTAGCATTTGGTAAGAATCTCTGAAC
Duet2-F	CTCGAGTCTGGTAAAGAAACCGCT
Duet2-R	CATATGTATATCTCCTTCTTATACTTAACTAATATACTAAG
pACYC-Duet-site1-F	GGATCCGAATTCGAGCTCG
pACYC-Duet-site1-R	CATGGTATATCTCCTTATTAAAGTTAAAC
AMPP2toDuet1-F	TAATAAGGAGATATACCATGGCTCAAACCAAAGTAGTTGTCG
AMPP2toDuet1-R	TCTGTTCGACTTAAGCATTATGGTTTGGCTAAGGGTCTTGTG
Duet_Site1-F	TAATGCTTAAGTCGAACAGAAAGTA

Duet_Site1-R	CATGGTATATCTCCTTATTAAAGTTAAACAA
Duet_Site3_F	AATTAACCTAGGCTGCTGCCAC
Duet_Site3_R	ATGTGCTGGCGTTCAAATTTCG
GNAT7toDuet3_F	AATTTGAACGCCAGCACATGCCTGCATTAGGAAATTAATACGACTC
GNAT7toDuet3_R	GTGGCAGCAGCCTAGGTTAACTTTCTGTTCGACTTAAGCATTATG
GNAT7toDuet2_F	AAGAAGGAGATATACATATGGCGTTCCGTCCCGT
GNAT7toDuet2_R	GTTTCTTTACCAGACTCGAGTTACATGGACATGATTGGGGAGG
**RbcL**	
RbcL-ΔS2-F	GATATACCATGCCACAAACAGAGACTAAAGC
RbcL-ΔS2-R	CTGTTTGTGGCATGGTATATCTCCTTCTTAA
RbcL-S2A-F	GAAGGAGATATACCATGGCACCACAAACAGAGAC
RbcL-S2A-R	GTCTCTGTTTGTGGTGCCATGGTATATCTCCTTC
RbcL-P3KO-F	GTCACAAACAGAGACTAAAGCA
RbcL-P3KO-R	GTCTCTGTTTGTGACATATGTA
RbcL-P3A_F	CATGTCAGCTCAAACAGAGACTAAAGCA
RbcL-P3A_R	CTGTTTGAGCTGACATGGTATATCTCCTTCTTAAA
RbcL-S2K.O_P3A_F	TACCATGGCTCAAACAGAGACTAAAGCA
RbcL-S2K.O_P3A_R	CTGTTTGAGCCATGGTATATCTCCTTCTTAAAGTTA
RbcL_ΔCter8-F	TAAGCTAGAAATAATTTTGTTTAACTTTAAGA
RbcL_ΔCter8-R	GGTTGGGAAGTTAAATGTGATC
RbcL_I472stop-F	CTTCCCAACCTAAGATAAATTAGATG
RbcL_I472stop-R	TATCTTAGGTTGGGAAGTTAAATGT
RbcL_D476stop-F	AAATTATAAGGCCAAGAGTAAGCTAGAAATAATTTTGT
RbcL_D476stop-R	GCTTACTCTTGGCCTTATAATTTATCGATGG
RbcL_K474stop-F	CGATTAATTAGATGGCCAAGAGTAAGCT
RbcL_K474stop-R	GGCCATCTAATTAATCGATGGTTGGG
**Rca**	
RCAtopET15-F	TGTATTTCCAGGGCCATATGGCTGTGAAAGAAGACAAACAAACC
RCAtopET15-R	CTTTGTTAGCAGCCGGATCCTTAAAAGTTGTAGACACAGGTTCCATC
RCAβ_F	AGAGGAAAAGGAGCCCAGCAAGTAAGGATCCGGCTGCTAACAA
RCAβ_R	CTGGGCTCCTTTTCCTCTGTTTTTCCGTAGAAAGTTCCGCG
RCAβ_GKtopET15-R	TGTTAGCAGCCGGATCCTTATTTTCCGTAGAAAGTTCCGCG

Table S3. Primers used for cloning.

**Primer name**	**Sequence 5' to 3'**	**Purpose**
SALK_088209C_LP	GTTTCACTCGAGTCTTGCAGG	Sequence *ampp2-3 and ampp2-4*
SALK_088209C_RP	TTGATGATGGTTTGGCTAAG	Sequence *ampp2-3* and *ampp2-4*
SALK_136128.24.75.RP	ATTGAGGTGCGACTTTGTCAC	Sequence *ampp2-3* and *ampp2-4*
SALK_002058.48.00_RP	AAAACCATCTTGCATTGAACG	Sequence *ampp2-2*
SALK_002058.48.00_LP	GATGCTGCTGAGGAATTGAAC	Sequence *ampp2-2*
SALK_122528.49.75_RP	CAAGGTTTAGATCCCAAAGCC	Sequence *ampp2-1*
SALK_122528.49.75_LP	TCAATTATTGGTCCAAGTGGG	Sequence *ampp2-1*
SALK_075759.4960_RP	ACAGCTGCAGTTGTTTGAACC	Sequence *ampp2-5*
SALK_075759.4960_RP	GAGCTTTTTGTTTTCACAGCG	Sequence *ampp2-5*
SALK_LB1.3	ATTTTGCCGATTTCGGAAC	T-DNA left border
qPCRAPP2FW1	TGGTTGATGTTTCTTTGCTGTC	qPCR1 (Exons 12-13)
qPCRAPP2REV1	TTGATCATGGTTTGGCTAAGGG	qPCR1 (Exons 12-13)
qPCR_APP_FW2	GTGTTCCCAGAAGGTACTCC	qPCR2 (Exons 9-11)
qPCR_APP_REV2	TGAAGGAGATTCTCAATTCGGA	qPCR2 (Exons 9-11)
qPCRAPP2FW2	CATGGGTGCTTAATCTGAGAGG	qPCR3 (Exons 4-5)
qPCRAPP2REV2	CGTAAGGTCTAAGTTCGATCCC	qPCR3 (Exons 4-5)

Table S4. Primers used for AMPP2 gene KO characterization in *A. thaliana* lines

DATASETS

Data S1: N-terminomics analysis of five independents putative AMPP2 KO lines show altered RbcL Ser_2 processing in four of them
See attached .xlxs document (legend plus four sheets).

Data S2: Significantly altered proteins observed in *amppp2-4* and *gnat7-1* lines compared to WT
See attached .xlxs document (legend plus two sheets).

Data S3. N-terminomics analysis of two independents putative GNAT7 KO lines show altered RbcL Pro acetylation
See attached .xlxs document (legend plus 16 sheets).

REFERENCES

1. M. Calvin, A. A. Benson, The path of carbon in photosynthesis. *Science* **107**, 476–480 (1948).

2. R. J. Ellis, The most abundant protein in the world. *Trends Biochem. Sci.* **4**, 241–244 (1979).

3. J. A. Raven, Rubisco: Still the most abundant protein of Earth? *New Phytol.* **198**, 1–3 (2013).

4. Y. M. Bar-On, R. Milo, The global mass and average rate of rubisco. *Proc. Natl. Acad. Sci. U.S.A.* **116**, 4738–4743 (2019).

5. T. Mae, A. Makino, K. Ohira, Changes in the amounts of ribulose bisphosphate carboxylase synthesized and degraded during the life span of rice leaf (*Oryza sativa* L.). *Plant Cell Physiol.* **24**, 1079–1086 (1983).

6. E. Carmo-Silva, J. C. Scales, P. J. Madgwick, M. A. Parry, Optimizing Rubisco and its regulation for greater resource use efficiency. *Plant Cell Environ.* **38**, 1817–1832 (2015).

7. L. Avila-Ospina, M. Moison, K. Yoshimoto, C. Masclaux-Daubresse, Autophagy, plant senescence, and nutrient recycling. *J. Exp. Bot.* **65**, 3799–3811 (2014).

8. F. Domínguez, F. J. Cejudo, Chloroplast dismantling in leaf senescence. *J. Exp. Bot.* **72**, 5905–5918 (2021).

9. M. Hayer-Hartl, F. U. Hartl, Chaperone machineries of Rubisco–The most abundant enzyme. *Trends Biochem. Sci.* **45**, 748–763 (2020).

10. U. Feller, I. Anders, T. Mae, Rubiscolytics: Fate of Rubisco after its enzymatic function in a cell is terminated. *J. Exp. Bot.* **59**, 1615–1624 (2008).

11. R. L. Houtz, A. R. Portis Jr., The life of ribulose 1,5-bisphosphate carboxylase/oxygenase—Posttranslational facts and mysteries. *Arch. Biochem. Biophys.* **414**, 150–158 (2003).

12. H. Ishida, M. Izumi, S. Wada, A. Makino, Roles of autophagy in chloroplast recycling. *Biochim. Biophys. Acta* **1837**, 512–521 (2014).

13. J. Amaral, A. K. M. Lobo, E. Carmo-Silva, Regulation of Rubisco activity in crops. *New Phytol.* **241**, 35–51 (2024).

14. R. L. Houtz, L. Poneleit, S. B. Jones, M. Royer, J. T. Stults, Posttranslational modifications in the amino-terminal region of the large subunit of Ribulose- 1,5-bisphosphate carboxylase/oxygenase from several plant epecies. *Plant Physiol.* **98**, 1170–1174 (1992).

15. H. Aksnes, R. Ree, T. Arnesen, Co-translational, post-translational, and non-catalytic roles of N-terminal acetyltransferases. *Mol. Cell* **73** 1097–1114 (2019).

16. C. Giglione, S. Fieulaine, T. Meinnel, N-terminal protein modifications: Bringing back into play the ribosome. *Biochimie* **114**, 134–146 (2015).

17. A. Varshavsky, N-degron and C-degron pathways of protein degradation. *Proc. Natl. Acad. Sci. U.S.A.* **116**, 358–366 (2019).

18. T. Meinnel, C. Giglione, N-terminal modifications, the associated processing machinery, and their evolution in plastid-containing organisms. *J. Exp. Bot.* **73**, 6013–6033 (2022).

19. C. Giglione, A. Serero, M. Pierre, B. Boisson, T. Meinnel, Identification of eukaryotic peptide deformylases reveals universality of N-terminal protein processing mechanisms. *EMBO J.* **19**, 5916–5929 (2000).

20. L. M. Dirk, M. A. Williams, R. L. Houtz, Eukaryotic peptide deformylases. Nuclear-encoded and chloroplast- targeted enzymes in *Arabidopsis*. *Plant Physiol.* **127**, 97–107 (2001).

21. C. Giglione, O. Vallon, T. Meinnel, Control of protein life-span by N-terminal methionine excision. *EMBO J.* **22**, 13–23 (2003).

22. C. Giglione, T. Meinnel, Evolution-driven versatility of N terminal acetylation in photoautotrophs. *Trends Plant Sci.* **26**, 375–391 (2021).

23. W. V. Bienvenut, A. Brünje, J.-B. Boyer, J. S. Mühlenbeck, G. Bernal, I. Lassowskat, C. Dian, E. Linster, T. V. Dinh, M. M. Koskela, V. Jung, J. Seidel, L. K. Schyrba, A. Ivanauskaite, J.

Eirich, R. Hell, D. Schwarzer, P. Mulo, M. Wirtz, T. Meinnel, C. Giglione, I. Finkemeier, Dual lysine and N-terminal acetyltransferases reveal the complexity underpinning protein acetylation. *Mol. Syst. Biol.* **16**, e9464 (2020).

24. W. V. Bienvenut, D. Sumpton, A. Martinez, S. Lilla, C. Espagne, T. Meinnel, C. Giglione, Comparative large scale characterization of plant versus mammal proteins reveals similar and idiosyncratic N-α-acetylation features. *Mol. Cell. Proteomics* **11**, M111.015131 (2012).

25. N. McTiernan, I. Kjosås, T. Arnesen, Illuminating the impact of N-terminal acetylation: From protein to physiology. *Nat. Commun.* **16**, 703 (2025).

26. M. M. Koskela, A. Brünje, A. Ivanauskaite, M. Grabsztunowicz, I. Lassowskat, U. Neumann, T. V. Dinh, J. Sindlinger, D. Schwarzer, M. Wirtz, E. Tyystjärvi, I. Finkemeier, P. Mulo, Chloroplast acetyltransferase NSI is required for state transitions in *Arabidopsis thaliana*. *Plant Cell* **30**, 1695–1709 (2018).

27. A. Serero, C. Giglione, T. Meinnel, Distinctive features of the two classes of eukaryotic peptide deformylases. *J. Mol. Biol.* **314**, 695–708 (2001).

28. H. Aigner, R. H. Wilson, A. Bracher, L. Calisse, J. Y. Bhat, F. U. Hartl, M. Hayer-Hartl, Plant RuBisCo assembly in *E. coli* with five chloroplast chaperones including BSD2. *Science* **358**, 1272–1278 (2017).

29. R. H. Wilson, G. Thieulin-Pardo, F. U. Hartl, M. Hayer-Hartl, Improved recombinant expression and purification of functional plant Rubisco. *FEBS Lett.* **593**, 611–621 (2019).

30. O. Király, L. Guan, E. Szepessy, M. Tóth, Z. Kukor, M. Sahin-Tóth, Expression of human cationic trypsinogen with an authentic N terminus using intein-mediated splicing in aminopeptidase P deficient *Escherichia coli*. *Protein Expr. Purif.* **48**, 104–111 (2006).

31. T. Yoshimoto, N. Murayama, T. Honda, H. Tone, D. Tsuru, Cloning and expression of aminopeptidase P gene from *Escherichia coli* HB101 and characterization of expressed enzyme. *J. Biochem.* **104**, 93–97 (1988).

32. F. Frottin, A. Martinez, P. Peynot, S. Mitra, R. C. Holz, C. Giglione, T. Meinnel, The proteomics of N-terminal methionine cleavage. *Mol. Cell. Proteomics* **5**, 2336–2349 (2006).

33. T. Meinnel, J. B. Boyer, C. Giglione, The global acetylation profiling pipeline for quick assessment of protein N-Acetyltransferase specificity in cellulo. *Methods Mol. Biol.* **2718**, 137–150 (2023).

34. B. Belenghi, F. Acconcia, M. Trovato, M. Perazzolli, A. Bocedi, F. Polticelli, P. Ascenzi, M. Delledonne, AtCYS1, a cystatin from Arabidopsis thaliana, suppresses hypersensitive cell death. *Eur. J. Biochem.* **270**, 2593–2604 (2003).

35. A. Porodko, A. Cirnski, D. Petrov, T. Raab, M. Paireder, B. Mayer, D. Maresch, L. Nika, M. L. Biniossek, P. Gallois, O. Schilling, C. Oostenbrink, M. Novinec, L. Mach, The two cathepsin B-like proteases of *Arabidopsis thaliana* are closely related enzymes with discrete endopeptidase and carboxydipeptidase activities. *Biol. Chem.* **399**, 1223–1235 (2018).

36. M. Coppola, L. Mach, P. Gallois, Plant cathepsin B, a versatile protease. *Front. Plant Sci.* **15**, 1305855 (2024).

37. K. Valegård, D. Hasse, I. Andersson, L. H. Gunn, Structure of Rubisco from *Arabidopsis thaliana* in complex with 2-carboxyarabinitol-1,5-bisphosphate. *Acta Crystallogr. D. Struct. Biol.* **74**, 1–9 (2018).

38. M. A. Parry, A. J. Keys, P. J. Madgwick, A. E. Carmo-Silva, P. J. Andralojc, Rubisco regulation: A role for inhibitors. *J. Exp. Bot.* **59**, 1569–1580 (2008).

39. K. Baerenfaller, C. Massonnet, S. Walsh, S. Baginsky, P. Bühlmann, L. Hennig, M. Hirsch-Hoffmann, K. A. Howell, S. Kahlau, A. Radziejwoski, D. Russenberger, D. Rutishauser, I. Small, D. Stekhoven, R. Sulpice, J. Svozil, N. Wuyts, M. Stitt, P. Hilson, C. Granier, W. Gruissem, Systems-based analysis of Arabidopsis leaf growth reveals adaptation to water deficit. *Mol. Syst. Biol.* **8**, 606 (2012).

40. M. Flecken, H. Wang, L. Popilka, F. U. Hartl, A. Bracher, M. Hayer-Hartl, Dual functions of a Rubisco activase in metabolic repair and recruitment to carboxysomes. *Cell* **183**, 457–473.e20 (2020).

41. Y. Lan, K. A. Mott, Determination of apparent K(m) values for Ribulose 1,5-bisphosphate carboxylase/oxygenase (Rubisco) activase using the spectrophotometric assay of Rubisco activity. *Plant Physiol.* **95**, 604–609 (1991).

42. J. Ng, Z. Guo, O. Mueller-Cajar, Rubisco activase requires residues in the large subunit N terminus to remodel inhibited plant Rubisco. *J. Biol. Chem.* **295**, 16427–16435 (2020).

43. S. J. Chen, X. Wu, B. Wadas, J. H. Oh, A. Varshavsky, An N-end rule pathway that recognizes proline and destroys gluconeogenic enzymes. *Science* **355**, 366 (2017).

44. S. J. Chen, L. Kim, H. K. Song, A. Varshavsky, Aminopeptidases trim Xaa-Pro proteins, initiating their degradation by the Pro/N-degron pathway. *Proc. Natl. Acad. Sci. U.S.A.* **118**, e2115430118 (2021).

45. E. Linster, F. L. Forero Ruiz, P. Miklankova, T. Ruppert, J. Mueller, L. Armbruster, X. Gong, G. Serino, M. Mann, R. Hell, M. Wirtz, Cotranslational N-degron masking by acetylation promotes proteome stability in plants. *Nat. Commun.* **13**, 810 (2022).

46. R. D. Etherington, M. Bailey, J. B. Boyer, L. Armbruster, X. Cao, J. C. Coates, T. Meinnel, M. Wirtz, C. Giglione, D. J. Gibbs, Nt-acetylation-independent turnover of SQUALENE EPOXIDASE 1 by Arabidopsis DOA10-like E3 ligases. *Plant Physiol.* **193**, 2086–2104 (2023).

47. P. C. Loewen, A. L. Didychuk, J. Switala, R. Perez-Luque, I. Fita, M. C. Loewen, Structure of Pisum sativum Rubisco with bound ribulose 1,5-bisphosphate. *Acta Crystallogr. Sect. F Struct. Biol. Cryst. Commun.* **69**, 10–14 (2013).

48. Y. Sun, J. Li, L. Zhang, R. Lin, Regulation of chloroplast protein degradation. *J. Genet. Genomics* **50**, 375–384 (2023).

49. M. S. Otegui, Vacuolar degradation of chloroplast components: Autophagy and beyond. *J. Exp. Bot.* **69**, 741–750 (2018).

50. M. Havé, A. Marmagne, F. Chardon, C. Masclaux-Daubresse, Nitrogen remobilization during leaf senescence: Lessons from Arabidopsis to crops. *J. Exp. Bot.* **68**, 2513–2529 (2017).

51. C. A. Carrión, M. L. Costa, D. E. Martínez, C. Mohr, K. Humbeck, J. J. Guiamet, In vivo inhibition of cysteine proteases provides evidence for the involvement of 'senescence-associated vacuoles' in chloroplast protein degradation during dark-induced senescence of tobacco leaves. *J. Exp. Bot.* **64**, 4967–4980 (2013).

52. A. Guiboileau, L. Avila-Ospina, K. Yoshimoto, F. Soulay, M. Azzopardi, A. Marmagne, J. Lothier, C. Masclaux-Daubresse, Physiological and metabolic consequences of autophagy deficiency for the management of nitrogen and protein resources in Arabidopsis leaves depending on nitrate availability. *New Phytol.* **199**, 683–694 (2013).

53. M. Kozak, Recognition of AUG and alternative initiator codons is augmented by G in position +4 but is not generally affected by the nucleotides in positions +5 and +6. *EMBO J.* **16**, 2482–2492 (1997).

54. M. W. Webster, Initiation of translation in bacteria and chloroplasts. *J. Mol. Biol.* **437**, 169137 (2025).

55. A. I. Flamholz, N. Prywes, U. Moran, D. Davidi, Y. M. Bar-On, L. M. Oltrogge, R. Alves, D. Savage, R. Milo, Revisiting trade-offs between Rubisco kinetic parameters. *Biochemistry* **58**, 3365–3376 (2019).

56. T. D. Pollard, Actin and actin-binding proteins. *Cold Spring Harb. Perspect. Biol.* **8**, a018226 (2016).

57. P. Haahr, R. A. Galli, L. G. van den Hengel, O. B. Bleijerveld, J. Kazokaitė-Adomaitienė, J. Y. Song, L. J. Kroese, P. Krimpenfort, M. P. Baltissen, M. Vermeulen, C. A. C. Ottenheijm, T. R. Brummelkamp, Actin maturation requires the ACTMAP/C19orf54 protease. *Science* **377**, 1533–1537 (2022).

58. T. Arnesen, H. Aksnes, Actin finally matures: Uncovering machinery and impact. *Trends Biochem. Sci.* **48**, 414–416 (2023).

59. T. Asensio, C. Dian, J. B. Boyer, F. Rivière, T. Meinnel, C. Giglione, A continuous assay set to screen and characterize novel protein N-acetyltransferases unveils rice general control non-repressible 5-related N-acetyltransferase 2 activity. *Front. Plant Sci.* **13**, 832144 (2022).

60. J. M. Werneke, J. M. Chatfield, W. L. Ogren, Alternative mRNA splicing generates the two ribulosebisphosphate carboxylase/oxygenase activase polypeptides in spinach and Arabidopsis. *Plant Cell* **1**, 815–825 (1989).

61. A. Ivanauskaite, M. Rantala, L. Laihonen, M. M. Konert, N. Schwenner, J. S. Mühlenbeck, I. Finkemeier, P. Mulo, Loss of chloroplast GNAT acetyltransferases results in distinct metabolic phenotypes in Arabidopsis. *Plant Cell Physiol.* **64**, 549–563 (2023).

62. Z. Adam, F. Frottin, C. Espagne, T. Meinnel, C. Giglione, Interplay between N-terminal methionine excision and FtsH protease is essential for normal chloroplast development and function in Arabidopsis. *Plant Cell* **23**, 3745–3760 (2011).

63. P. Monassa, F. Rivière, C. Dian, F. Frottin, C. Giglione, T. Meinnel, Biochemical and structural analysis of N-myristoyltransferase mediated protein tagging. *Methods Enzymol.* **684**, 135–166 (2023).

64. A. E. Carmo-Silva, C. Barta, M. E. Salvucci, Isolation of ribulose-1,5-bisphosphate carboxylase/oxygenase from leaves. *Methods Mol. Biol.* **684**, 339–347 (2011).

65. J. C. Scales, M. A. Parry, M. E. Salvucci, A non-radioactive method for measuring Rubisco activase activity in the presence of variable ATP: ADP ratios, including modifications for measuring the activity and activation state of Rubisco. *Photosynth. Res.* **119**, 355–365 (2014).

66. H. I. Fraser, M. Kvaratskhelia, M. F. White, The two analogous phosphoglycerate mutases of *Escherichia coli*. *FEBS Lett.* **455**, 344–348 (1999).

67. N. Loganathan, Y. C. Tsai, O. Mueller-Cajar, Characterization of the heterooligomeric red-type rubisco activase from red algae. *Proc. Natl. Acad. Sci. U.S.A.* **113**, 14019–14024 (2016).

68. C. Barta, A. E. Carmo-Silva, M. E. Salvucci, Rubisco activase activity assays. *Methods Mol. Biol.* **684**, 375–382 (2011).

69. W. V. Bienvenut, C. Giglione, T. Meinnel, SILProNAQ: A convenient approach for proteome-wide analysis of protein N-termini and N-terminal acetylation quantitation. *Methods Mol. Biol.* **1574**, 17–34 (2017).

70. W. V. Bienvenut, J. P. Scarpelli, J. Dumestier, T. Meinnel, C. Giglione, EnCOUNTer: A parsing tool to uncover the mature N-terminus of organelle-targeted proteins in complex samples. *BMC Bioinformatics* **18**, 182 (2017).

71. A. T. Kong, F. V. Leprevost, D. M. Avtonomov, D. Mellacheruvu, A. I. Nesvizhskii, MSFragger: Ultrafast and comprehensive peptide identification in mass spectrometry-based proteomics. *Nat. Methods* **14**, 513–520 (2017).

72. F. Yu, S. E. Haynes, A. I. Nesvizhskii, IonQuant enables accurate and sensitive label-free quantification with FDR-controlled match-between-runs. *Mol. Cell. Proteomics* **20**, 100077 (2021).

73. K. C. Cho, J. W. Kang, Y. Choi, T. W. Kim, K. P. Kim, Effects of peptide acetylation and dimethylation on electrospray ionization efficiency. *J. Mass Spectrom.* **51**, 105–110 (2016).

74. S. Tyanova, T. Temu, P. Sinitcyn, A. Carlson, M. Y. Hein, T. Geiger, M. Mann, J. Cox, The Perseus computational platform for comprehensive analysis of (prote)omics data. *Nat. Methods* **13**, 731–740 (2016).

75. N. Colaert, K. Helsens, L. Martens, J. Vandekerckhove, K. Gevaert, Improved visualization of protein consensus sequences by iceLogo. *Nat. Methods* **6**, 786–787 (2009).

76. Y. Yamauchi, Y. Ejiri, Y. Toyoda, K. Tanaka, Identification and biochemical characterization of plant acylamino acid-releasing enzyme. *J. Biochem.* **134**, 251–257 (2003).

77. S. N. W. Hoernstein, B. Özdemir, N. van Gessel, A. A. Miniera, B. Rogalla von Bieberstein, L. Nilges, J. Schweikert Farinha, R. Komoll, S. Glauz, T. Weckerle, F. Scherzinger, M. Rodriguez-Franco, S. J. Müller-Schüssele, R. Reski, A deeply conserved protease, acylamino acid-releasing enzyme (AARE), acts in ageing in Physcomitrella and Arabidopsis. *Commun. Biol.* **6**, 61 (2023).

78. C. Giglione, A. Boularot, T. Meinnel, Protein N-terminal methionine excision. *Cell. Mol. Life Sci.* **61**, 1455–1474 (2004).

79. M. Ferro, S. Brugière, D. Salvi, D. Seigneurin-Berny, M. Court, L. Moyet, C. Ramus, S. Miras, M. Mellal, S. Le Gall, S. Kieffer-Jaquinod, C. Bruley, J. Garin, J. Joyard, C. Masselon, N. Rolland, AT_CHLORO, a comprehensive chloroplast proteome database with subplastidial localization and curated information on envelope proteins. *Mol. Cell. Proteomics* **9**, 1063–1084 (2010).

80. A. Martinez, J. A. Traverso, B. Valot, M. Ferro, C. Espagne, G. Ephritikhine, M. Zivy, C. Giglione, T. Meinnel, Extent of N-terminal modifications in cytosolic proteins from eukaryotes. *Proteomics* **8**, 2809–2831 (2008).

81. F. Jeanmougin, J. D. Thompson, M. Gouy, D. G. Higgins, T. J. Gibson, Multiple sequence alignment with Clustal X. *Trends Biochem. Sci.* **23**, 403–405 (1998).

82. J. Kim, J. E. Mullet, Ribosome-binding sites on chloroplast rbcL and psbA mRNAs and light-induced initiation of D1 translation. *Plant Mol. Biol.* **25**, 437–448 (1994).

83. T. Hirose, M. Sugiura, Functional Shine-Dalgarno-like sequences for translational initiation of chloroplast mRNAs. *Plant Cell Physiol.* **45**, 114–117 (2004).

84. M. Hanaoka, K. Kanamaru, H. Takahashi, K. Tanaka, Molecular genetic analysis of chloroplast gene promoters dependent on SIG2, a nucleus-encoded sigma factor for the plastid-encoded RNA polymerase, in *Arabidopsis thaliana*. *Nucleic Acids Res.* **31**, 7090–7098 (2003).

85. W. Chi, B. He, N. Manavski, J. Mao, D. Ji, C. Lu, J. D. Rochaix, J. Meurer, L. Zhang, RHON1 mediates a Rho-like activity for transcription termination in plastids of *Arabidopsis thaliana*. *Plant Cell* **26**, 4918–4932 (2014).

86. R. Grzela, J. Nusbaum, S. Fieulaine, F. Lavecchia, W. V. Bienvenut, C. Dian, T. Meinnel, C. Giglione, The C-terminal residue of phage Vp16 PDF, the smallest peptide deformylase, acts as an offset element locking the active conformation. *Sci. Rep.* **7**, 11041 (2017).

87. L. Kolberg, U. Raudvere, I. Kuzmin, P. Adler, J. Vilo, H. Peterson, g:Profiler-interoperable web service for functional enrichment analysis and gene identifier mapping (2023 update). *Nucleic Acids Res.* **51**, W207–W212 (2023).
